# Selenoxide Elimination Triggers Enamine Hydrolysis to Primary and Secondary Amines: A Combined Experimental and Theoretical Investigation

**DOI:** 10.3390/molecules26092770

**Published:** 2021-05-08

**Authors:** Giovanni Ribaudo, Marco Bortoli, Erika Oselladore, Alberto Ongaro, Alessandra Gianoncelli, Giuseppe Zagotto, Laura Orian

**Affiliations:** 1 Dipartimento di Medicina Molecolare e Traslazionale, Università degli Studi di Brescia, Viale Europa 11, 25123 Brescia, Italy; giovanni.ribaudo@unibs.it (G.R.); e.oselladore@unibs.it (E.O.); a.ongaro005@unibs.it (A.O.); alessandra.gianoncelli@unibs.it (A.G.); 2 Dipartimento di Scienze Chimiche, Università degli Studi di Padova, Via Marzolo 1, 35131 Padova, Italy; marco.bortoli@unipd.it; 3 Departament de Química, Institut de Química Computacional i Catàlisi, Universitat de Girona, C/M.A. Capmany 69, 17003 Girona, Spain; 4 Dipartimento di Scienze del Farmaco, Università degli Studi di Padova, Via Marzolo 5, 35131 Padova, Italy; giuseppe.zagotto@unipd.it

**Keywords:** selenoxide elimination, one-pot, imine-enamine, reaction mechanism, DFT calculations, selenium

## Abstract

We discuss a novel selenium-based reaction mechanism consisting in a selenoxide elimination-triggered enamine hydrolysis. This one-pot model reaction was studied for a set of substrates. Under oxidative conditions, we observed and characterized the formation of primary and secondary amines as elimination products of such compounds, paving the way for a novel strategy to selectively release bioactive molecules. The underlying mechanism was investigated using NMR, mass spectrometry and density functional theory (DFT).

## 1. Introduction

In the past few decades, the selenoxide elimination reaction has been used to obtain alkenes and has been largely applied in the synthesis of small molecules such as natural products and bioactive compounds [1,2,3,4]. Generally, the organoselenides required for such a reaction can be straightforwardly synthesized from electrophilic phenylselenyl chloride or using diphenyl diselenides as precursors for the nucleophilic selenate. In the presence of oxidants, such as peroxides or other reactive oxygen species (ROS), organoselenium compounds are readily oxidized to selenoxides [5,6,7]. Then, an intramolecular *syn* elimination occurs in opportune substrates, involving the hydrogen atom in vicinal position with respect to the selenium nucleus. This leads to the formation of the corresponding *trans*-olefine [8,9,10].

**Scheme 1:**
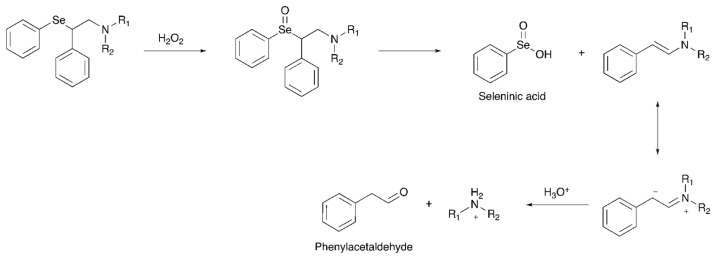
Proposed mechanism for selenoxide elimination-triggered enamine hydrolysis.

In the context of our studies on selenium-based derivatives of compounds of pharmaceutical interest, we observed a peculiar behavior of amino organoselenides [10]. In particular, under oxidative conditions, we previously detected the formation of primary and secondary amines from mono- or disubstituted 2-phenyl-2-(phenylselanyl)ethan-1-amines (Scheme 1). 

Among the functional groups of biological relevance, the amino moiety is one of the most frequently occurring. Nevertheless, drugs containing primary or secondary amines, despite being very common, are endowed with some pharmacokinetic limitations such as poor diffusion through membranes or blood–brain barrier under particular physiologic conditions. Moreover, instability affects such compounds, as amines physiologically undergo metabolic phase 1 transformations such as oxidations or dealkylations or conjugations in phase 2. These limitations can be partially overcome by derivatization to generate prodrugs that can be “unmasked” under certain conditions that can be pH-, redox- or enzyme-dependent [11]. Recent reports showed that selenoxide elimination can be employed by drug-like derivatives responsive to reactive oxygen species (ROS) in innovative targeted therapeutic strategies [12]. Moreover, we previously showed that compounds endowed with bioactivity can be generated after release from such substrates. Molecules selectively providing elimination products in response to oxidative stress represent attractive tools, in particular in the context of central nervous system (CNS) drugs [10].

Taking the step from these considerations, we investigated and characterized a one-pot model reaction consisting in a selenoxide elimination-triggered enamine hydrolysis that allows the formation of primary and secondary amines under oxidative conditions.

Additionally, this combined process is endowed with synthetic value, as it represents an innovative approach for obtaining primary and secondary amines as elimination products under these specific conditions. Although selenium-catalyzed organic reactions, and particularly selenoxide elimination, have been extensively studied [13,14,15], no examples of selenium-based mechanisms for obtaining such amines were previously reported in the literature.

## 2. Results and Discussion

**Scheme 2:**
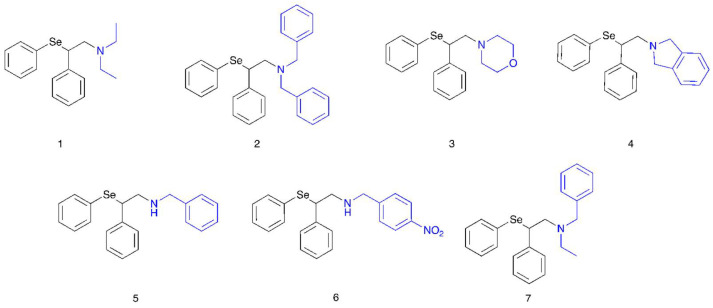
Chemical structures of the studied compounds. The portion of the molecules providing the resulting amine is highlighted in blue.

From the point of view of the molecular mechanism, the considered oxidation-triggered elimination is distinctive of organoselenium compounds having protons in the β-position with respect to the chalcogen atom. This process occurs through a *syn* mechanism and promotes the formation of olefins with high *trans* selectivity [8]. The reaction is initiated by the oxidation of the selenium atom to the corresponding selenoxide, a step which can be induced by different agents such as hydrogen peroxide, meta-chloroperoxybenzoic acid (mCPBA) and ozone [7,16,17]. Then, an intramolecular *syn* elimination takes place: the Se-C bond breaks producing the *trans*-olefin and selenenic acid, that is readily oxidized to seleninic acid [10]. Interestingly, in the case of the studied compounds (Scheme 2), an enamine is produced after the oxidation–elimination step. This can be protonated on both the nitrogen atom and the β-carbon atom in acidic conditions. The latter event is favored and the molecule, rearranging to an iminium ion and undergoing hydrolysis, subsequently produces the corresponding amine (Scheme 1) [18,19].

In the field of synthetic organic chemistry, the preparation of primary aliphatic amines can be achieved by *Gabriel* synthesis [20], by azidation–reduction [21], by *Leuckart* reaction [22] or its variation involving the use of benzylamine and hydrogenolysis [23]. In analogy, secondary aliphatic amines can be obtained through different processes: direct alkylation [24], reductive amination [25] and the *Fukuyama* amine synthesis [26]. In the current study, we aimed at investigating the formation of primary and secondary amines based on the combination of known selenoxide elimination and enamine hydrolysis reactions. In particular, this reaction was studied in aqueous solution and under oxidative conditions. Thus, mono- and disubstituted symmetric, asymmetric and cyclic derivatives reported in Scheme 2 were synthesized and reacted with hydrogen peroxide in the presence of water. The overall reaction was studied in detail by NMR spectroscopy, mass spectrometry and DFT calculations.

**Scheme 3:**
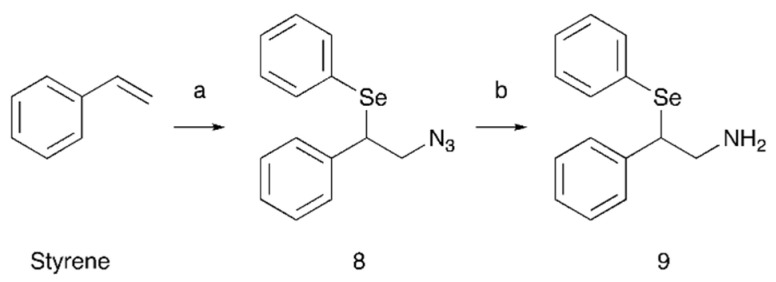
(**a**) (diacetoxyiodo)benzene/diphenyl diselenide/NaN3/DCM; (**b**) LiAlH4/THF.

Compound **8** was synthesized by styrene azido-phenylselenenylation with the hypervalent iodine reagent (diacetoxyiodo)benzene, sodium azide and diphenyl diselenide in dichloromethane (Scheme 3). The reaction involves the formation of an azido radical that, after the addition to the double bond of the styrene, generates a carbon radical which is then trapped by diphenyldiselenide to provide the product [27]. Racemic compound **9** was obtained by reduction of the azido group of compound **8** with lithium aluminum hydride in THF (Scheme 3).

Then, **9** was subjected to different alkylation reactions to obtain compounds **1**–**7** as hydrochlorides (Scheme 2; see Figures S1–S27 in Supplementary Materials for NMR and mass spectra). In particular, compounds **1**–**4** were obtained through direct alkylation using the opportune halide, such as iodoethane, benzyl bromide, bis(2-bromoethyl) ether and α,α′-dibromo-*o*-xylene. Compounds **5** and **6** were obtained by reductive amination of compound **9** with benzaldehyde and *p*-nitrobenzaldehyde, respectively. By reacting compound **5** with iodoethane, the corresponding tertiary amine (compound **7**) was prepared.

First, the combined reaction was studied in detail for compound **3** in aqueous solution by ^1^H-NMR. This substrate was chosen as a model also because the corresponding elimination product, morpholine, represents an outstanding example of pharmacologically relevant moiety. Interestingly, it can be found in drugs acting on CNS (e.g., in the antidepressants reboxetine and viloxazine and in the withdrawn stimulant phenmetrazine), and which are used to treat pathologies where oxidative stress has been demonstrated to play a role on disease onset and progression [28,29]. More in detail, compound **3** was dissolved in deuterated water and reacted with 1.2 equivalents of hydrogen peroxide at room temperature.

**Figure 1:**
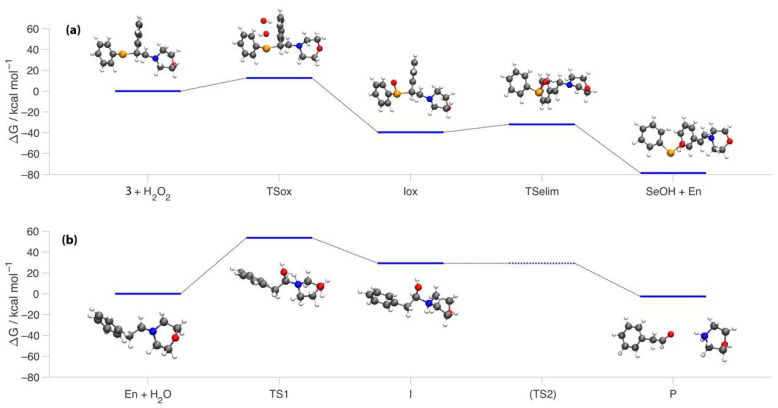
Gibbs free energies of the relevant intermediates and transition states characterized in water for the oxidation and subsequent elimination of compound **3**: (**a**) corresponds to the top reaction of Scheme 1 whereas (**b**) to the bottom one. Water and hydrogen peroxide molecules are not shown in the minimum energy structures for clarity. Level of theory: COSMO- ZORA-OLYP/TZ2P.

**Figure 2:**
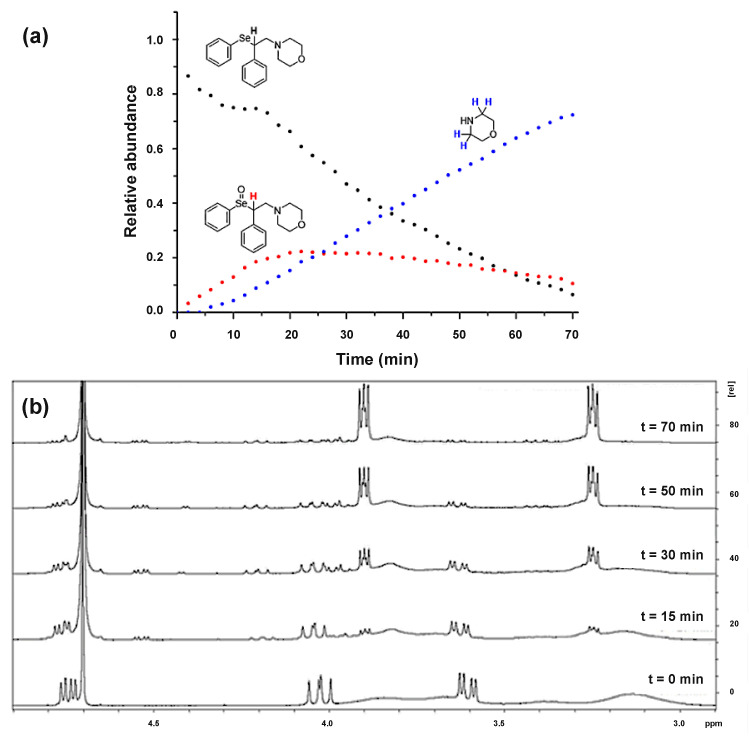
Reaction profiles for compound **3**, intermediate and reaction products. The signals considered for relative quantification are depicted as in the following: • starting material, • intermediate, • secondary amine. Integrals were measured using acetonitrile as internal standard (**a**). Detailed views of representative NMR spectra showing the considered signals (**b**).

The analysis of this reaction showed the progressive disappearance of the α-hydrogen signal, paralleled by the appearance of the signals corresponding to the two selenoxide diastereoisomers (Figure S28 in Supplementary Materials). Calculations at COSMO-ZORA-OLYP/TZ2P level of theory [30,31] showed that in water, this reaction displays an energy barrier of 14.5 kcal mol^−1^ which separates the reactants from the selenoxide form (**Iox**, see Figure 1a) lying at −39.5 kcal mol^−1^. As the reaction further proceeded, the signals of such intermediates disappeared and the subsequent formation of the two particular signals corresponding to morpholine hydrochloride (3.25 and 3.90 ppm) was observed. After 70 min, 72% relative abundance of secondary amine was observed in the NMR spectrum. At the same time point, the relative abundance of the starting material was < 10% (Figure 2). The mechanism for the last part of the reaction was computationally modelled in three elementary steps. First, starting from the selenoxide, an intramolecular elimination reaction requiring 7.6 kcal mol^−1^ takes place (**TSelim**, Figure 1a) that results in the cleavage of one C-Se bond and the formation of selenenic acid (the further oxidation of selenenic acid to seleninic acid detected experimentally was not computationally investigated) [9] and the corresponding enamine (**SeOH** + **En**, Figure 1a) with a ∆G of −76.7 kcal mol^−1^ relative to the starting reactants. Finally, the process that was involved in the cleavage of the C-N bond to give the final morpholine molecule consist in two steps. The first requires the addition of an H_3_O^+^ ion to the enamine with an activation energy of 51.8 kcal mol^−1^ (**TS1**, Figure 1b) and leads to an intermediate which is destabilized by 25.5 kcal mol^−1^ (**I**, Figure 1b). The final reaction of the C-N bond cleavage leads to the formation of morpholine and phenylacetaldehyde (**P**, Figure 1b) which are found to be 6.4 kcal mol^−1^ more stable than the starting enamine. The reaction involves a proton transfer from the OH moiety of I to the N atom. This step requires a somewhat extended network of explicit water molecules to be successfully modelled [32]. The inclusion of explicit water molecules in mechanisms that involve proton transfers usually helps to recover more favorable energetics [32,33,34,35]. Nevertheless, mechanistic features are not modified by this inclusion and conformational studies on the model hydrogen bond network to obtain the most stable configuration are quite tricky. As the detailed mechanistic analysis of this reaction is out of the scope of this work, the effect of explicit water molecules was not addressed in this context. All the solvation contributions were incorporated in the polarizing effect of the dielectric continuum employed in the calculations. On the same basis, the reaction was investigated by ^1^H-NMR spectroscopy for compounds **1**–**7** (see Figures S31, S33 and S36 in Supplementary Materials for representative NMR and mass spectra), demonstrating the disappearance of starting material and the formation of the corresponding products (43–93% relative abundance). Electrospray mass spectrometry (ESI-MS) studies, which were performed in parallel, confirmed the identity of involved intermediates, including enamines, and products. More in detail, primary and secondary amines generated after the oxidation of compound **1**–**7** were characterized using positive ionization ESI-MS on the same samples, while negative ionization ESI-MS analysis confirmed the presence of seleninic acid and benzoic acid as final oxidation products. The latter very likely generates from phenylacetaldehyde oxidation (see Figures S29, S30, S32, S34, S35 and S37 in Supplementary Materials for mass spectra) [36].

Following this NMR-scale mechanistic investigation and to gain further insight on the underlying processes, the reactions involving compounds **1**–**7** were scaled up and performed on the same substrates, in order to isolate the elimination products, verify the identity and calculate the yield. Compounds were subjected to an overnight oxidation using H_2_O_2_ and the corresponding amines were isolated with an average yield of 68%, with a best isolated yield of 90%. ^1^H-NMR, ^13^C-NMR and ESI-MS analysis confirmed the identity of the products, thus demonstrating the formation of primary and secondary amines in the laboratory scale (see Supplementary Materials for experimental procedures, yields, computational details and Figures S38–S58 for spectra).

## 3. Materials and Methods

### 3.1. Chemistry

Commercially available chemicals were purchased from Sigma-Aldrich and used without any further purification if not specified elsewhere. NMR experiments were performed on an Avance III 400 (Bruker, Billerica, MA, USA) spectrometer (frequencies: 400.13, 100.62 MHz for ^1^H and ^13^C nuclei, respectively) equipped with a multinuclear inverse z-field gradient probe head (5 mm). For data processing, TopSpin 4.0.8 software was used, and the spectra were calibrated using solvent signal (^1^H-NMR, δ_H_ = 7.26 ppm for CDCl_3_, δ_H_ = 3.31 ppm for CD_3_OD, δ_H_ = 2.50 ppm for DMSO-*d6*; ^13^C-NMR, δ_C_ = 77.16 ppm for CDCl_3_, 39.52 ppm for DMSO-*d6*, δ_C_ = 49.00 for CD_3_OD). Multiplicities are reported as follows: s, singlet; d, doublet; t, triplet; q, quartet; m, multiplet; b, broad; dd, doublet of doublets. Mass spectra were recorded by direct infusion ESI on a LCQ Fleet ion trap (Thermo Fisher Scientific, Waltham, MA, USA) mass spectrometer. For data processing, Qual Browser Thermo Xcalibur 4.0.27.13 software was used. ESI parameters for samples acquired in positive ionization mode: spray voltage 3.2 kV, capillary temperature 160 °C, capillary voltage 43 V. ESI parameters for samples acquired in negative ionization mode: spray voltage 5.0 kV, capillary temperature 160 °C, capillary voltage −8 V.

### 3.2. Synthesis of Compound ***1***

Compound **9** (100 mg, 0.36 mmol) was dissolved in iodoethane (2 mL) and DIPEA (190 µL, 1.08 mmol) was added to the solution that was then deaerated, purged with nitrogen and refluxed under stirring at 90 °C for 2 h. After TLC showed the disappearance of the starting material (DCM/MeOH/NH_3_, 97.5/2.0/0.5), the mixture was cooled to r.t. and the solvent was evaporated under reduced pressure. DCM (30 mL) was added, and the solution was washed with 1 M KOH (3 × 10 mL), dried over magnesium sulphate and evaporated at reduced pressure obtaining a viscous brownish liquid which was purified by chromatographic column (silica gel, DCM/MeOH/NH_3_, 97.5/2.0/0.5). The hydrochloride salt was then prepared, dissolving the product in anhydrous diethyl ether and adding a 2 M solution of HCl in diethyl ether dropwise.

White solid, 119 mg, 0.32 mmol, 89%. ^1^H-NMR (CD_3_OD, 400 MHz): 7.63–7.61 (m, 2H), 7.46–7.34 (m, 8H), 4.82 (dd, *J* = 10.7, 4.5 Hz, 1H), 4.06 (dd, *J* = 14.0, 10.7 Hz, 1H), 3.52 (dd, *J* = 14.0, 4.5 Hz, 1H), 3.20–3.06 (m, 4H), 1.19–1.10 (m, 6H); ^13^C{^1^H} NMR (CD_3_OD, 101 MHz): 138.8, 137.2, 130.6, 130.4, 130.3, 129.7, 129.2, 129.0, 56.3, 41.8, 9.0, 8.5; (ESI+) *m*/*z* calcd for C_18_H_24_NSe^+^ [M + H]^+^: 334.11, found: 333.95.

### 3.3. Synthesis of Compound ***2***

Compound **9** (100 mg, 0.36 mmol) was dissolved in EtOH (5 mL). Benzyl bromide (87 µL, 124 mg, 0.73 mmol) and DIPEA (190 µL, 1.08 mmol) were added to the solution that was deaerated, purged with nitrogen and refluxed under stirring at 90 °C for 2 h. After TLC showed the disappearance of the starting material (hexane/diethyl ether, 6/1), the mixture was cooled to r.t. and the solvent was evaporated under reduced pressure. DCM (30 mL) was added, and the obtained solution was washed with 1 M KOH (3 × 10 mL), dried over magnesium sulphate and evaporated under reduced pressure. The crude product was purified by column chromatography (silica gel, hexane/DCM, 9/1) obtaining a viscous liquid. The hydrochloride salt was then prepared dissolving the product in anhydrous diethyl ether and adding a 2 M solution of HCl in diethyl ether dropwise.

White solid, 93 mg, 0.19, 52%. ^1^H-NMR (CD_3_OD, 400 MHz): δ_H_ (ppm) 7.57–7.20 (m, 18H), 6.80–6.78 (m, 2H), 4.56–4.43 (m, 3H), 4.31 (d, *J* = 12.7 Hz, 1H), 4.17 (d, *J* = 12.6 Hz, 1H), 3.87 (dd, *J* = 13.9, 10.9 Hz, 1H), 3.61 (dd, *J* = 13.9, 5.0 Hz, 1H); ^13^C{^1^H} NMR (CD_3_OD, 101 MHz): 138.0, 137.8, 132.9, 132.6, 131.6, 130.7, 130.5, 130.5, 130.3, 129.6, 128.7, 127.9, 60.4, 55.5, 41.6; (ESI+) *m*/*z* calcd for C_28_H_28_NSe^+^ [M + H]^+^: 458.14, found: 458.05.

### 3.4. Synthesis of Compound ***3***

Compound **9** (100 mg, 0.36 mmol) was dissolved in EtOH (5 mL). Bis(2-bromoethyl) ether (84 mg, 0.36 mmol) and DIPEA (190 µL, 1.08 mmol) were added to the solution that was deaerated, purged with nitrogen and refluxed under stirring at 90 °C for 2 h. After TLC showed disappearance of starting material (DCM/MeOH/TEA, 95/4.5/0.5), the mixture was cooled to r.t. and the solvent was evaporated under reduced pressure, DCM (30 mL) was added and the so obtained solution was washed with 1 M KOH (3 × 10 mL), dried over magnesium sulphate and evaporated at reduced pressure. The crude product was purified by column chromatography (silica gel, DCM/MeOH/TEA, 95/4.5/0.5), obtaining a viscous liquid. The hydrochloride salt was then prepared, dissolving the product in anhydrous diethyl ether and adding a 2 M solution of HCl in diethyl ether dropwise. 

White solid, 70 mg, 0.18 mmol, 50%. ^1^H-NMR (CD_3_OD, 400 MHz): 7.52–7.50 (m, 2H), 7.38–7.28 (m, 8H), 4.92 (dd, *J* = 10.4, 4.7 Hz, 1H), 4.13–4.07 (m, 1H), 4.00–3.69 (m, 4H), 3.64 (dd, *J* = 13.6, 4.7 Hz, 1H), 3.24–3.20 (m, 4H); ^13^C{^1^H} NMR (CD_3_OD, 101 MHz): 139.2, 136.9, 130.5, 130.2, 130.0, 129.5, 129.1, 129.1, 64.6, 62.1, 53.7, 41.4; (ESI+) *m*/*z* calcd for C_18_H_22_NOSe^+^ [M + H]^+^: 347.95, found: 347.98.

### 3.5. Synthesis of Compound ***4***

Compound **9** (150 mg, 0.54 mmol) was dissolved in EtOH (6 mL). α,α′-dibromo-o-xylene (154 mg, 0.54 mmol) and K_2_CO_3_ (225 mg, 1.62 mmol) were added to the solution that was deareated, purged with nitrogen and refluxed under stirring at 90 °C for 3 h. After TLC showed the disappearance of the starting material (hexane/diethyl ether, 6/1), the mixture was cooled to r.t. and the solvent was evaporated under reduced pressure, DCM (30 mL) was added and the obtained solution was washed with 1 M KOH (3 × 10 mL), dried over magnesium sulphate and evaporated under reduced pressure. The crude product was purified by column chromatography (silica gel, hexane/diethyl ether, 89.75/9.75/0.5) obtaining a viscous liquid. The hydrochloride salt was then prepared dissolving the product in anhydrous diethyl ether and adding a 2 M solution of HCl in diethyl ether dropwise. 

Green solid, 45 mg, 0.11 mmol, 20 %. ^1^H NMR (CD_3_OD, 400 MHz): 7.53–7.51 (m, 2H), 7.40–7.30 (m,12H), 4.82–4.79 (m, 1H), 4.67–4.58 (m, 4H), 4.29 (dd, *J* = 13.2, 10.2 Hz, 1H) 3.96 (dd, *J* = 13.2, 5.7 Hz, 2H); ^13^C{^1^H} NMR (CD_3_OD, 101 MHz): 138.9, 137.1, 134.8, 130.5, 130.2, 130.1, 130.1, 129.6, 129.1, 128.7, 123.8, 60.4, 60.0, 43.2; (ESI+) *m*/*z* calcd for C_22_H_21_NSe^+^ [M + H]^+^: 379.08, found: 380.00.

### 3.6. Synthesis of Compound ***5***

Compound **9** (100 mg, 0.36 mmol) was dissolved in dry MeOH (5 mL). Benzaldehyde (37 µL, 0.36 mmol) was added to the solution that was deaerated, purged with nitrogen and stirred at r.t. for 2 h. After TLC showed disappearance of starting materials (hexane/diethyl ether 6/1), the mixture was cooled in an ice bath and NaBH_4_ (16 mg, 0.43 mmol) was added in portions. The ice bath was removed, and the mixture was stirred at r.t. for other 2 h. After TLC showed the disappearance of the starting material (hexane/diethyl ether 6/1), the solution was cooled in an ice bath and first acidified to pH = 2 by the addition of 1 M HCl and the basified to pH = 14 by the addition of 8 M KOH. The solvent was evaporated under reduced pressure. DCM (30 mL) was added, and the solution was washed with 1 M KOH (3 × 10 mL), dried over magnesium sulphate and evaporated under reduced pressure, obtaining a viscous liquid. The crude product was purified by column chromatography (silica gel hexane/EtOAc, 9/1 with 0.5% TEA). The hydrochloride salt was then prepared, dissolving the product in anhydrous diethyl ether and adding a 2 M solution of HCl in diethyl ether dropwise.

White solid, 125 mg, 0.31 mmol, 86%. ^1^H-NMR (CDCl_3_, 400 MHz): 7.42–7.19 (m, 15H), 4.49 (dd, *J* = 7.9, 7.0 Hz, 1H), 3.0 (d, *J* = 3.4 Hz, 2H), 3.26 (dd, *J* = 12.6, 8.2 Hz, 1H), 3.16 (dd, *J* = 12.6, 7.0 Hz, 1H); ^13^C{^1^H} NMR (CDCl_3_, 101 MHz): 140.7, 140.0, 135.4, 128.8, 128.6, 128.5, 128.4, 128.1, 127.9, 127.8, 127.2, 127.0, 53.4, 53.3, 48.2; (ESI+) *m*/*z* calcd for C_21_H_22_NSe^+^ [M + H]^+^: 368.08, found: 367.93.

### 3.7. Synthesis of Compound ***6***

Compound **9** (150 mg, 0.36 mmol) was dissolved in dry MeOH (6 mL). p-Nitrobenzaldehyde (82 mg µL, 0.36 mmol) was added to the solution that was deareated, purged with nitrogen and stirred overnight at r.t. After TLC showed the disappearance of the starting material (6/1 hexane/diethyl ether), the mixture was cooled in an ice bath and NaBH_4_ (16 mg, 0.43 mmol) was added portion wise. The ice bath was removed, and the mixture was stirred at r.t. for other 2 h. After TLC showed the disappearance of the starting material (6/1 hexane/diethyl ether), the solution was cooled in an ice bath and first acidified to pH = 2 by the addition of 1 M HCl and the basified to pH = 14 by the addition of 8 M KOH. The solvent was evaporated under reduced pressure. DCM (30 mL) was added, and the solution was washed with 1 M KOH (3 × 10 mL), dried over magnesium sulphate and evaporated under reduced pressure, obtaining a viscous liquid. The crude product was purified by column chromatography (silica gel hexane/EtOAc, 9/1 with 0.5% TEA). The hydrochloride salt was then prepared, dissolving the product in anhydrous diethyl ether and adding a 2 M solution of HCl in diethyl ether dropwise.

White solid, 135 mg, 0.31 mmol, 87%. ^1^H-NMR (DMSO-*d*_6_, 400 MHz): δ_H_ (ppm) 9.56 (br, 1H), 9.25 (br, 1H), 8.24 (d, *J* = 8.9 Hz, 2H), 7.75 (d, *J* = 8.9 Hz, 2H), 7.44–7.42 (m, 2H), 7.36–7.25 (m, 8H), 4.88 (dd, *J* = 9.7, 5.6 Hz, 1H), 4.26 (s, 2H), 3.74–3.72 (m, 2H); ^13^C{^1^H} NMR (DMSO-*d*_6_, 101 MHz): 148.2, 139.5, 138.2, 134.8, 134.0, 131.9, 129.8, 129.2, 128.7, 128.5, 128.4, 124.0, 50.5, 49.8, 42.2; (ESI+) *m*/*z* calcd for C_21_H_21_N_2_O_2_Se^+^ [M + H]^+^: 413.08, found: 412.89.

### 3.8. Synthesis of Compound ***7***

Compound **5** (100 mg, 0.27 mmol) was dissolved in iodoethane (5 mL). The solution was deaerated, purged with nitrogen and refluxed under stirring at 90 °C for 2 h. After TLC showed the disappearance of the starting material (hexane/diethyl ether, 6/1), the mixture was cooled to r.t. and the solvent was evaporated under reduced pressure. DCM (30 mL) was added, and the solution was washed with 1 M KOH (3 × 10 mL), dried over magnesium sulphate and evaporated under reduced pressure obtaining a viscous colorless liquid. The crude product was purified by column chromatography (silica gel, hexane/EtOAc/TEA, 97/2.5/0.5) obtaining a viscous liquid. The hydrochloride salt was then prepared dissolving the product in anhydrous diethyl ether and adding a 2 M solution of HCl in diethyl ether dropwise.

White solid, 69 mg, 0.15 mmol, 57 %. ^1^H-NMR (CD_3_OD, 400 MHz): 7.51–7.06 (m, 15H), 4.77 (dd, *J* = 10.8, 4.7 Hz, 0.5H), 4.60 (dd, *J* = 10.5, 4.6 Hz, 0.5H), 4.39–4.22 (m, 2H), 4.06 (dd, *J* = 13.9, 10.6 Hz, 0.5H), 3.88 (dd, *J* = 13.9, 10.7 Hz, 0.5H), 3.63–3.48 (m, 1H), 3.30–3.13 (m, 2H), 1.27–1.22 (m, 3H); ^13^C{^1^H} NMR (CD_3_OD, 101 MHz): 138.8, 138.3, 137.4, 137.3, 132.3, 132.2, 131.4, 130.6, 130.5, 130.5, 130.4, 130.3, 130.3, 130.1, 129.7, 129.6, 129.0, 128.9, 128.8, 128.6, 58.8, 56.3, 56.1, 50.9, 50.2, 42.1, 41.4, 28.0, 8.9, 8.5; (ESI+) *m*/*z* calcd for C_23_H_26_NSe^+^ [M + H]^+^: 396.12, found: 395.94.

### 3.9. Synthesis of Compound ***8***

Diphenyldiselenide (414 mg, 1.33 mmol), (diacetoxyiodo)benzene (1.000 g, 3.10 mmol) and sodium azide (346 mg, 5.32 mmol) were added to a solution of styrene (231 mg, 2.22 mmol) in DCM (20 mL). The mixture was deaerated and purged with nitrogen. After overnight stirring at r.t., a saturated solution of NaHCO_3_ (10 mL) was added, the organic phase was separated, and the aqueous phase was extracted with DCM (2 × 20 mL). The organic phases were reunited and evaporated at reduced pressure. The obtained yellow liquid was purified by column chromatography (silica gel, pure hexane then 90:10 hexane/diethylether), obtaining a colorless liquid.

Colorless liquid, 257 mg, 0.85 mmol, 38%. ^1^H NMR (CDCl_3_, 400 MHz,): δ_H_ 7.54–7.51 (m, 2H), 7.37–7.27 (m, 8H), 4.44 (dd, *J* = 9.7, 5.7 Hz, 1H), 3.91 (dd, *J* = 12.5, 9.8 Hz, 1H), 3.75 (dd, *J* = 12.5, 5.8 Hz, 1H); ^13^C{^1^H} NMR (CDCl_3_, 101 MHz): 139.0, 135.7, 129.3, 128.9, 128.6, 128.5, 128.0, 127.9, 55.6, 46.5; (ESI+) *m*/*z* calcd for C_14_H_14_N_3_Se^+^ [M + H]^+^: 304.19, found: 304.15.

### 3.10. Synthesis of Compound ***9***

Compound **8** (1.010 g, 3.33 mmol) was dissolved in anhydrous THF (20 mL) and the solution was deaerated and purged with nitrogen. The system was equipped with a reflux apparatus and a solution of 1 M LiAlH_4_ (5 mL, 5.01 mmol) was added portion wise to the mixture; after the addition, the mixture was stirred for 2 h. After TLC showed disappearance of starting material (DCM/MeOH/NH_3_, 97.5/2.0/0.5), wet diethyl ether was slowly added to the solution and distilled water was then added. The resulting bi-phasic mixture was filtered, and the aqueous phase was separated and extracted with diethyl ether (2 × 20 mL). The organic phase was dried over magnesium sulphate and evaporated at reduced pressure obtaining a viscous colorless liquid that was purified by column chromatography (silica gel, DCM/MeOH/NH_3_, 97.5/2.0/0.5).

Colorless liquid, 503 mg, 1.82 mmol, 55%. ^1^H NMR (CDCl_3_, 400 MHz): 7.53–7.51 (m, 2H), 7.37–7.27 (m, 8H), 4.43 (dd, *J* = 9.6, 5.7 Hz, 1H), 3.91 (dd, *J* = 12.6, 9.6 Hz, 1H), 3.74 (dd, *J* = 12.6, 5.7 Hz, 1H); ^13^C{^1^H} NMR (CDCl_3_, 101 MHz): 140.2, 135.1, 128.8, 128.7, 128.3, 127.8, 127.6, 127.0, 51.8, 46.8; (ESI+) *m*/*z* calcd for C_14_H_16_NSe^+^ [M + H]^+^: 278.04, found: 277.80.

### 3.11. Laboratory Scale Oxidation Procedures

#### 3.11.1. Oxidation of Compound **3**

Compound **3** (1.50 g, 3.92 mmol, 1 eq) was dissolved in 120 ml of distilled H_2_O in a round-bottom flask and H_2_O_2_ (530 μL, 4.70 mmol, 1.2 eq) was then added to the mixture. The solution was stirred overnight and checked by TLC. Further 1.2 eq of H_2_O_2_ were then added in order to consume the remained reactant. After TLC showed the disappearance of the starting material, the mixture was cooled in an ice bath, filtered and the filtrate was evaporated under reduced pressure, adding ethanol to facilitate solvent removal. The resulting yellowish precipitate was washed with small aliquots of acetone, obtaining the desired product as a white solid.

*Morpholine∙HCl*: white solid, 435 mg, 3.52 mmol, 90%; ^1^H-NMR (CD_3_OD, 400 MHz): 3.91–3.88 (m, 4H), 3.25–3.22 (m, 4H); ^13^C{^1^H} NMR (CD_3_OD, 101 MHz): 64.9, 44.6; (ESI+) *m*/*z* calcd for C_4_H_10_NO^+^ [M + H]^+^: 88.08, found: 87.93.

#### 3.11.2. Oxidation of Compound **1**, **4**, **5** and **7**

The selenoamine (1 eq) was dissolved in a MeOH/H_2_O mixture (85/15) in a round-bottom flask. 1.2 eq of H_2_O_2_ were then added to the mixture and the solution was stirred overnight. The solution was stirred overnight and checked by TLC. Further 1.2 eq of H_2_O_2_ were then added in order to consume the remained reactant. After TLC showed the disappearance of the starting material, the solvent was evaporated under reduced pressure. Water was added to the reaction flask in order to dissolve the desired product and eliminate most of the insoluble byproducts. The final compounds were isolated following the procedure described above. 

*Diethylamine∙HCl*: white solid; 55 mg, 0.50 mmol, 82%; ^1^H-NMR (CD_3_OD, 400 MHz): 3.06 (q, *J* = 7.3 Hz, 4H), 1.32 (t, *J* = 7.3 Hz, 6H); ^13^C{^1^H} NMR (CD_3_OD, 101 MHz): 43.5, 11.6; (ESI+) *m*/*z* calcd for C_4_H_12_N^+^ [M + H]^+^: 74.10, found: 73.9.

*Isoindoline∙HCl*: brown solid, 69 mg, 0.44 mmol, 69%; ^1^H NMR (DMSO-*d*_6_, 400 MHz): 10.21 (br, 2H), 7.52–7.36 (m, 4H), 4.48 (s, 4H); ^13^C{^1^H} NMR (DMSO-*d*_6_, 101 MHz): 134.4, 127.6, 122.4, 49.2; (ESI+) *m*/*z* calcd for C_8_H_10_N^+^ [M + H]^+^: 120.08, found: 119.98.

*Benzylamine∙HCl*: white solid, 183 mg, 1.27 mmol, 85%; ^1^H NMR (CD_3_OD, 400 MHz): 7.49–7.40 (m, 5H), 4.12 (s, 2H); ^13^C{^1^H} NMR (CD_3_OD, 101 MHz): 134.4, 130.2, 130.0, 129.9, 44.4; (ESI+) *m*/*z* calcd for C_7_H_10_N^+^ [M + H]^+^: 108.08, found: 107.88.

*N-ethylbenzylamine∙HCl*: white solid, 114 mg, 0.66 mmol, 75%; ^1^H-NMR (CD_3_OD, 400 MHz): 7.52–7.44 (m, 5H), 4.19 (s, 2H), 3.12 (q, *J* = 7.3 Hz, 2H), 1.34 (t, *J* = 7.3 Hz, 3H); ^13^C{^1^H} NMR (CD_3_OD, 101 MHz): 132.7, 130.9, 130.6, 130.3, 52.0, 43.8, 11.5; (ESI+) *m*/*z* calcd for C_9_H_14_N^+^ [M + H]^+^: 136.11, found: 135.97.

#### 3.11.3. Oxidation of Compound **2** and **6**

The compounds were obtained following the procedure reported above. Although, after the evaporation of the MeOH/H_2_O mixture and the addition of water, the mixture was acidified with 0.2 M HCl to pH 2–3 and heated for a couple of hours at 40 °C in order to facilitate the hydrolysis of the enamine. 

*Dibenzylamine∙HCl*: white solid, 47 mg, 0.22 mmol, 33%; ^1^H NMR (CD_3_OD, 400 MHz): 7.52–7.45 (m, 10H), 4.25 (s, 4H); ^13^C{^1^H} NMR (CD_3_OD, 101 MHz): 131.8, 130.1, 128.8, 128.5, 49.5; (ESI+) *m*/*z* calcd for C_14_H_16_N^+^ [M + H]^+^: 198.13, found: 198.06.

*p-Nitrobenzylamine*: white solid, 15 mg, 0.08 mmol, 45%; ^1^H NMR (DMSO-*d*_6_, 400 MHz): 8.78 (br, 3H), 8.26 (d, *J* = 8.2 Hz, 2H), 7.80 (d, *J* = 8.2 Hz, 2H), 4.17 (s, 2H); ^13^C{^1^H} NMR (DMSO-*d*_6_, 101 MHz): 147.4, 141.7, 130.2, 123.5, 41.3; (ESI+) *m*/*z* calcd for C_7_H_9_N_2_O_2_^+^ [M + H]^+^: 153.07, found: 152.93.

### 3.12. Computational Details

The oxidation–elimination–hydrolysis mechanism of compound **3** was modeled with an in silico approach based on DFT. The oxidation and elimination initial reactions (Scheme 1, top line) were modelled on the basis of a recent in-depth study on analogous reactions by some of the authors of this work [10]. The only difference with Scheme 1 is that the in the computational model, the direct product of the elimination process is the selenenic acid analogue of the seleninic acid. The oxidation from selenenic to seleninic acid was not investigated in silico. The oxidation reaction keeps the stereochemistry of the C carbon bonded to Se fixed and can take two pathways, leading to diastereomeric geometries of oxidized products (*R–R* and *R–S*). In light of the conclusions drawn in a recent work [10], only the pathway leading to the *R–R* diastereomer was investigated due to its more favorable reaction energies. The quantum chemistry calculations for the oxidation mechanism were performed using the Amsterdam Density Functional (ADF) [37,38,39]. The energy profiles were obtained from geometries and energies computed by using the OLYP functional [40,41], which is known to perform well for reactivity studies on organic compounds, and it has been recently benchmarked [30] and applied [31] to organic dichalcogenides and amines [42]. OLYP was combined with the TZ2P basis set for all the atoms [43]. The TZ2P basis set is of triple- quality and has been augmented with two sets of polarization functions. Core shells of the atoms (1s for C, F, N and O and up to 3p for Se) were treated by using the frozen-core approximation. Scalar relativistic effects were accounted for using the Zeroth Order Regular Approximation (ZORA) [44,45,46]. The numerical integration was performed by using the fuzzy-cell integration scheme developed by Becke [47,48]. Energy minima and transition states have been verified through vibrational analysis. All minima were found to have zero imaginary frequencies and all transition states have one that correspond to the mode of the reaction under consideration. For single point calculations in water, the conductor-like screening model was employed (COSMO), as implemented in ADF [49,50,51]. Water was parameterized using the default values in ADF, i.e., a dielectric constant of 78.39 and a solvent radius of 1.93 Å. The empirical parameter in the scaling function in the COSMO equation was set to 0.0. The radii of the atoms were taken to be MM3 radii divided by 1.2, giving 1.350 Å for H, 1.700 Å for C, 1.608 for N, 1.517 for O and 1.908 for Se [49,52]. This level of theory is referred to as (COSMO)-ZORA-OLYP/TZ2P. Geometry optimizations were conducted in the gas phase and frequency calculations were employed to establish the nature of the stationary points found (all real frequencies for minima and one imaginary frequency for transition states). Thereafter, single point calculations in COSMO water were used to obtain the ∆G values reported in the main text. Relative free Gibbs energies are shown for the gas phase calculations and for the water single points in Table S1.

## 4. Conclusions

Amines are present in a vast portion of biologically active compounds, and their relevance in medicinal chemistry is crucial. More specifically, such moieties are often present in CNS-targeting molecules. In several drugs or drug-like compounds, variously substituted amines represent a part of the pharmacophore or are employed to enhance water solubility. We here reported the experimental and in silico characterization of an innovative combined reaction consisting in a selenoxide elimination-triggered hydrolysis for the preparation and/or selective release under oxidative conditions of several model primary and secondary amines. This may allow an effective in situ release of high-value pharmaceutical compounds that overcomes multiple limitations to which amines are often subjected to in the organism. Moreover, the ability of this reaction to be triggered in an oxidizing environment is very promising for the development of amino-containing molecules that can be selectively activated only in case of excessive oxidative stress levels.

## Data Availability

The data presented in this study are available in the supplementary materials.

## Supplementary Materials

The following are available online, Figures S1–S27: NMR and ESI-MS characterization of compounds **1**–**9**; Figures S28–S37: representative ^1^H-NMR and ESI-MS spectra of the oxidation study with H_2_O_2_; Figures S38–S58: ^1^H-NMR, ^13^C-NMR and ESI-MS spectra of the isolated compounds obtained by oxidation with H_2_O_2_; Table S1: Gibbs free energies relative to free reactants for the selenoxide-triggered amine formation; Table S2: geometries of the optimized structures of the selenoxide-triggered amine formation reaction.

## Sample Availability

Not available.
