# Supplementary material for: Selenoxide Elimination Triggers Enamine Hydrolysis to Primary and Secondary Amines: A Combined Experimental and Theoretical Investigation
Source: Molecules. 2021 May 8;26(9):2770. doi: 10.3390/molecules26092770 (PMC8125833; doi:10.3390/molecules26092770)
Supplement: Supplementary file 1 [file molecules-26-02770-s001.zip › molecules-1204897-supplementary.pdf]

Supplementary Materials

Insights on Enamine Hydrolysis Triggered by Selenoxide Elimination Producing Primary and Secondary Amines

Giovanni Ribaudo[1], Marco Bortoli[2,3], Erika Oselladore[1], Alberto Ongaro[1], Alessandra Gianoncelli[1], Giuseppe Zagotto[4] and Laura Orian[2*]

[1] Dipartimento di Medicina Molecolare e Traslazionale, Università degli Studi di Brescia, Viale Europa 11, 25123 Brescia - Italy

[2] Dipartimento di Scienze Chimiche, Università degli Studi di Padova, Via Marzolo 1, 35131 Padova - Italy

[3] Institut de Química Computacional i Catàlisi and Departament de Química, Universitat de Girona, C/M.A. Capmany 69, 17003 Girona, Spain

[4] Dipartimento di Scienze del Farmaco, Università degli Studi di Padova, Via Marzolo 5, 35131 Padova - Italy

*Corresponding author: Prof. Laura Orian, Dipartimento di Scienze Chimiche, Università degli Studi di Padova, Via Marzolo 1, 35131 Padova - Italy. E-mail: laura.orian@unipd.it.

Table of contents

NMR and ESI-MS characterization of compounds 1-9 **S1**

Compound 1 *S1*

Compound 2 *S2*

Compound 3 *S4*

Compound 4 *S5*

Compound 5 *S7*

Compound 6 *S8*

Compound 7 *S10*

Compound 8 *S11*

Compound 9 *S13*

Representative ^{1}H-NMR and ESI-MS spectra of the oxidation study with H_2O_2 **S14**

Compound 3 *S14*

Compound 1 *S16*

Compound 5 *S17*

Compound 7 *S18*

^{1}H-NMR, ^{13}C-NMR and ESI-MS spectra of the isolated compounds obtained by oxidation with H_2O_2 **S19**

Diethylamine•HCl *S19*

Dibenzylamine•HCl *S20*

Morpholine•HCl *S23*

Isoindoline•HCl *S24*

Benzylamine•HCl *S26*

p-nitrobenzylamine•HCl *S27*

N-ethylbenzylamine•HCl *S29*

Table S1. Gibbs free energies relative to free reactants for the selenoxide-triggered amine formation **S31**

Table S2 Geometries of the optimized structures of the selenoxide-triggered amine formation reaction. **S31**

NMR and ESI-MS characterization of compounds 1-9

Compound 1

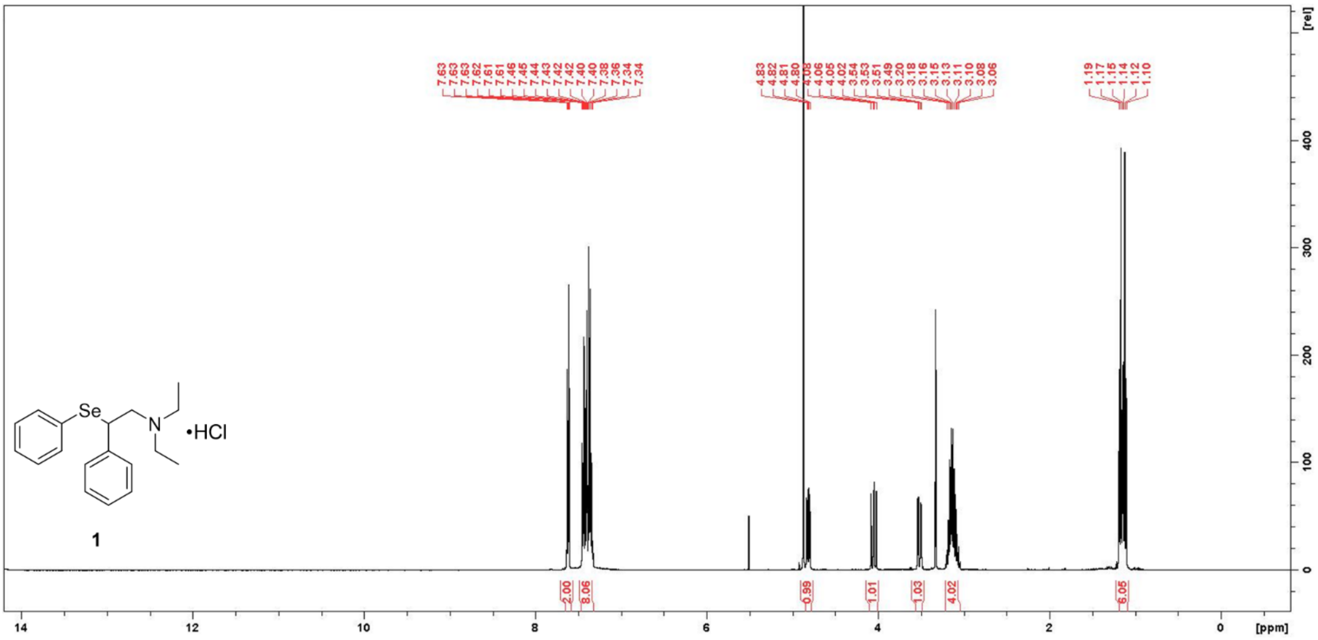

Figure S1. ^{1}H-NMR spectrum of compound **1**.

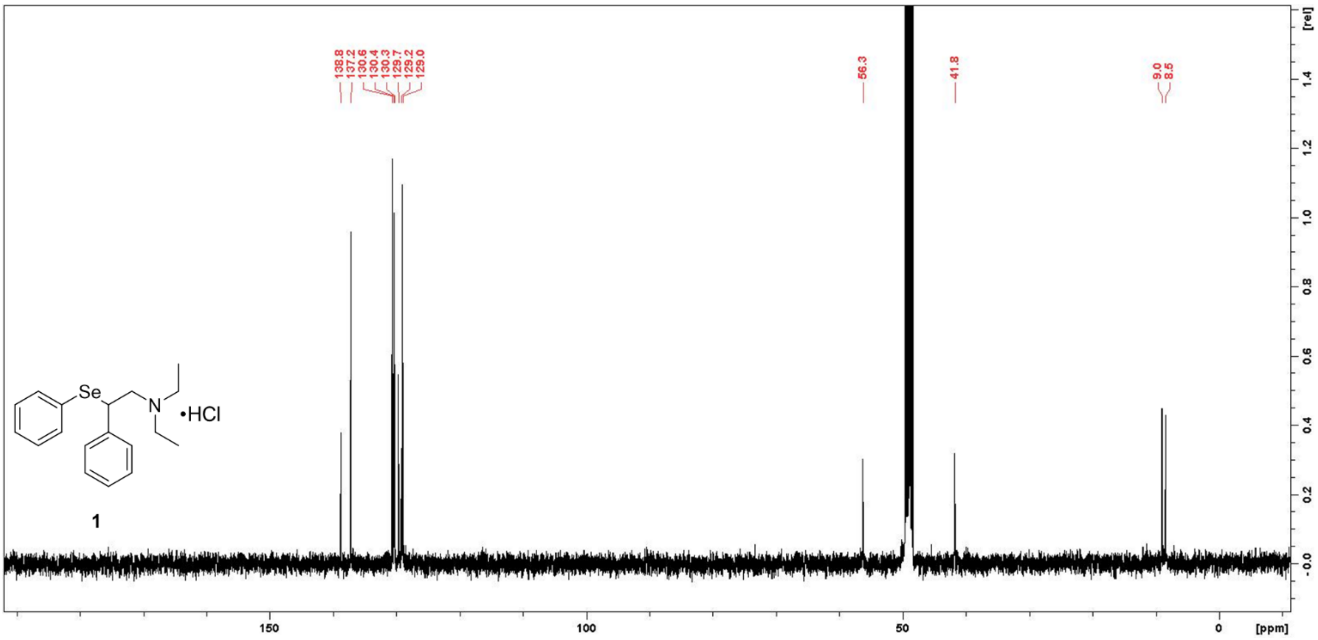

Figure S2. ^{13}C-NMR spectrum of compound **1**.

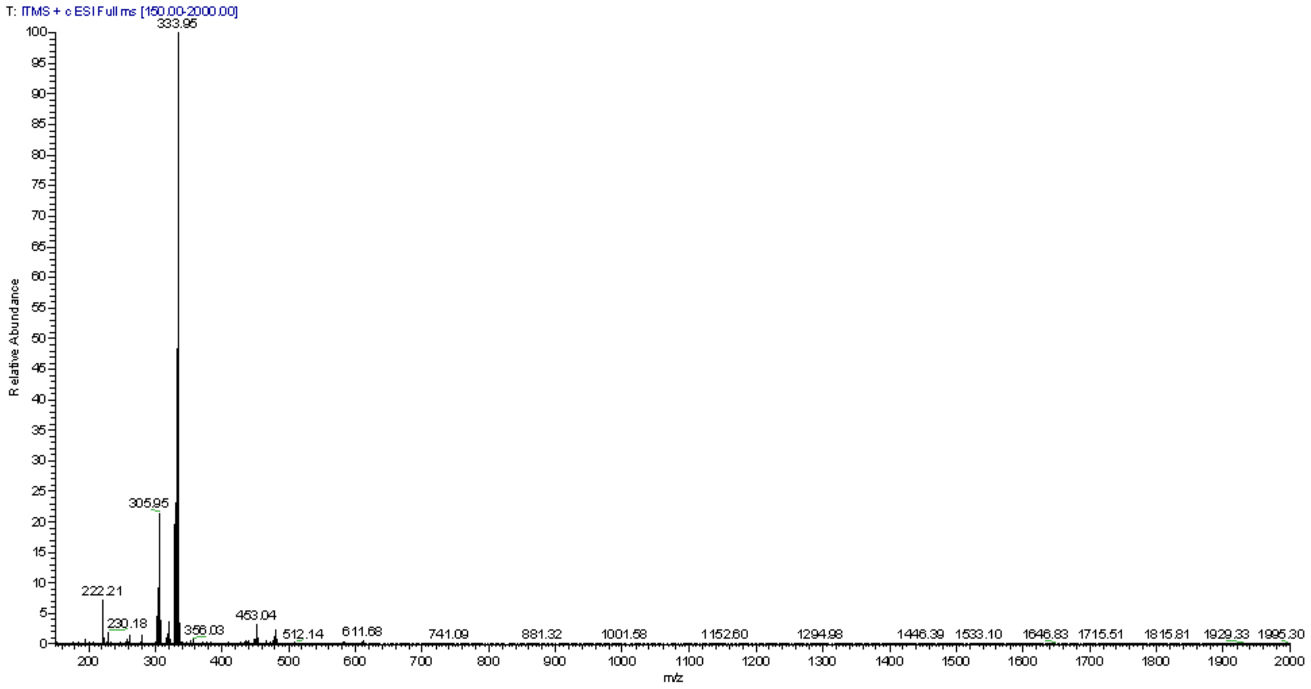

Figure S3. ESI-MS spectrum of compound **1**.

Compound 2

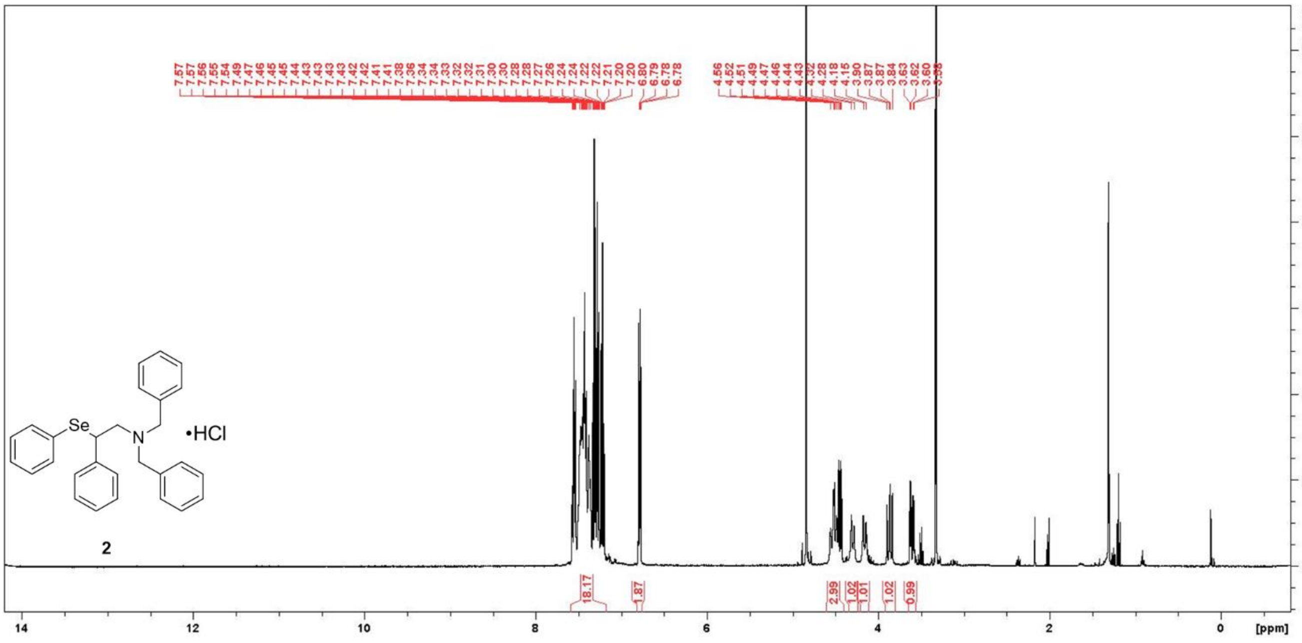

Figure S4. ^{1}H-NMR spectrum of compound **2** (δ_H 1.33 ppm = traces of silicon grease).

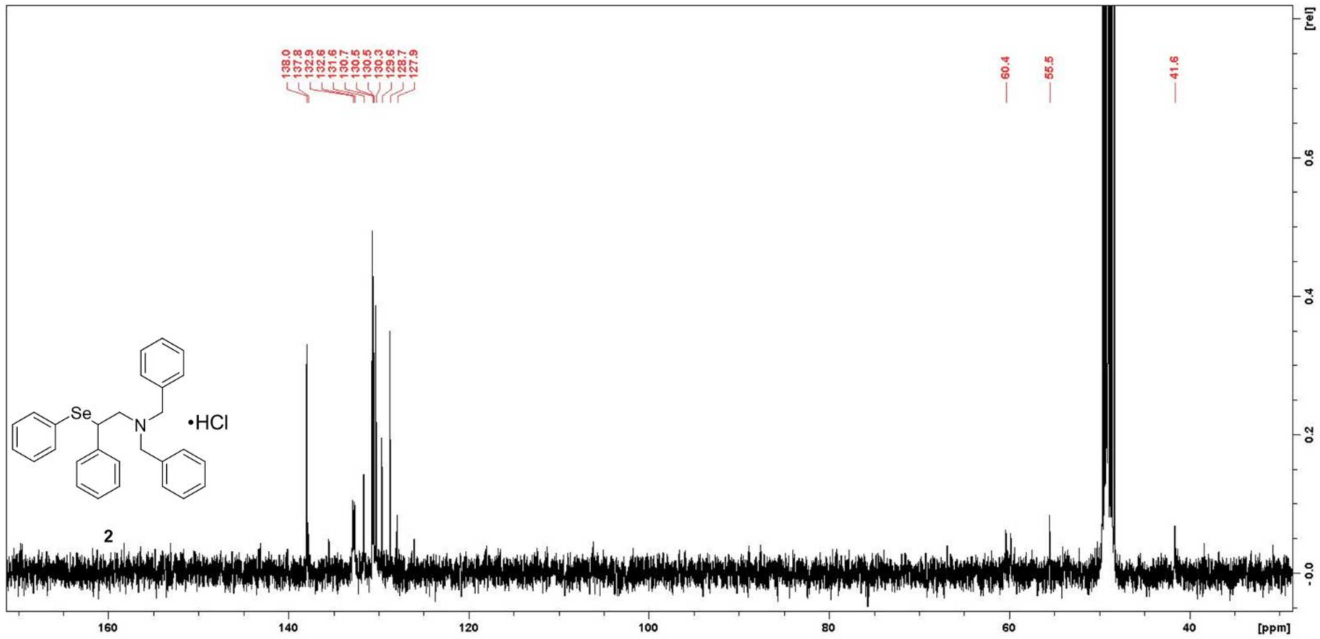

Figure S5. ^{13}C-NMR spectrum of compound **2**.

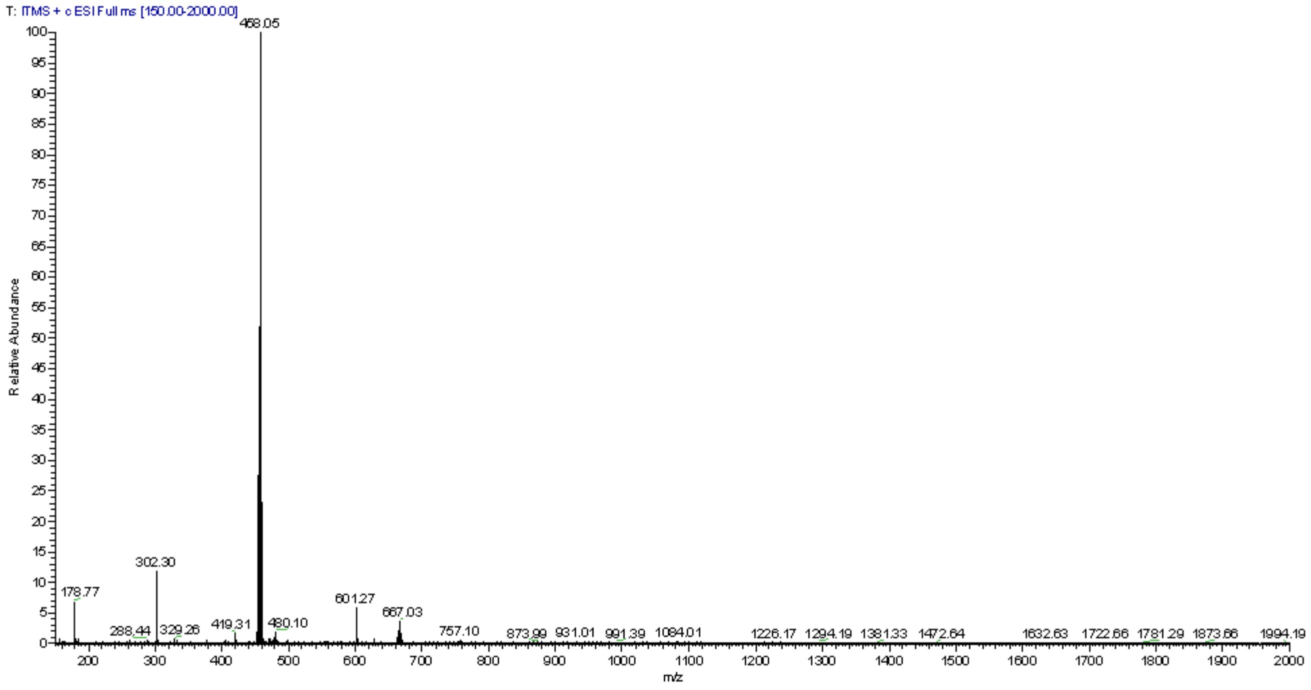

Figure S6. ESI-MS spectrum of compound **2**.

Compound 3

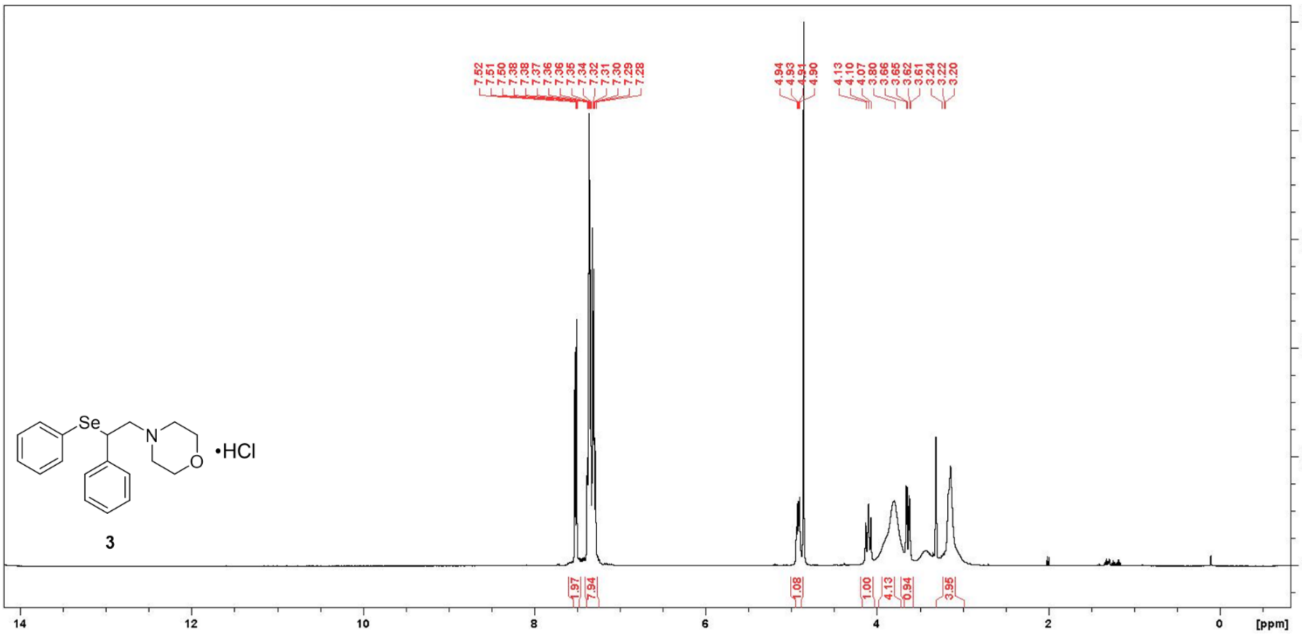

Figure S7. ^{1}H-NMR spectrum of compound **3**.

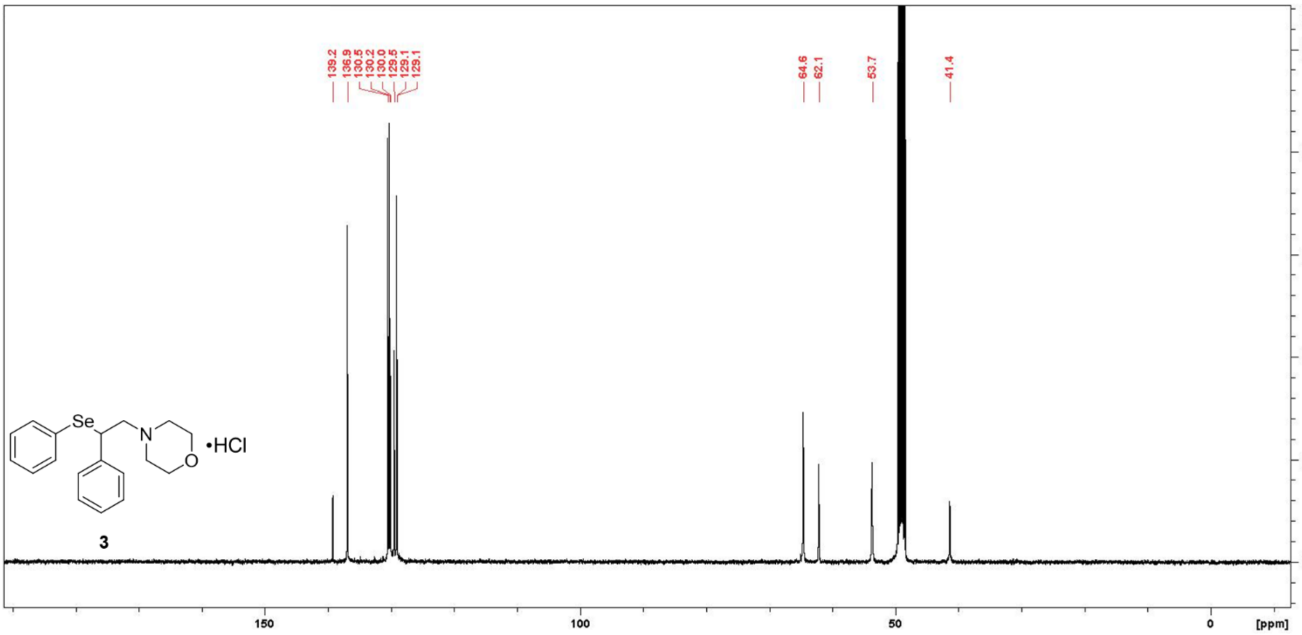

Figure S8. ^{13}C-NMR spectrum of compound **3**.

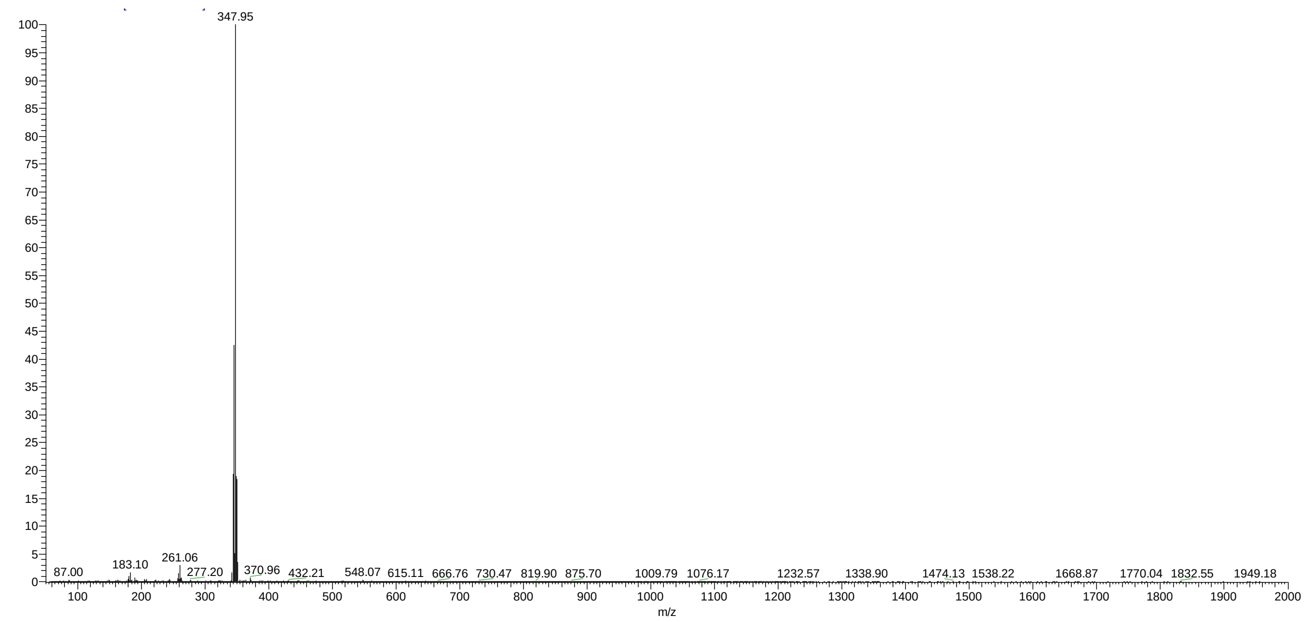

Figure S9. ESI-MS spectrum of compound **3**.

Compound 4

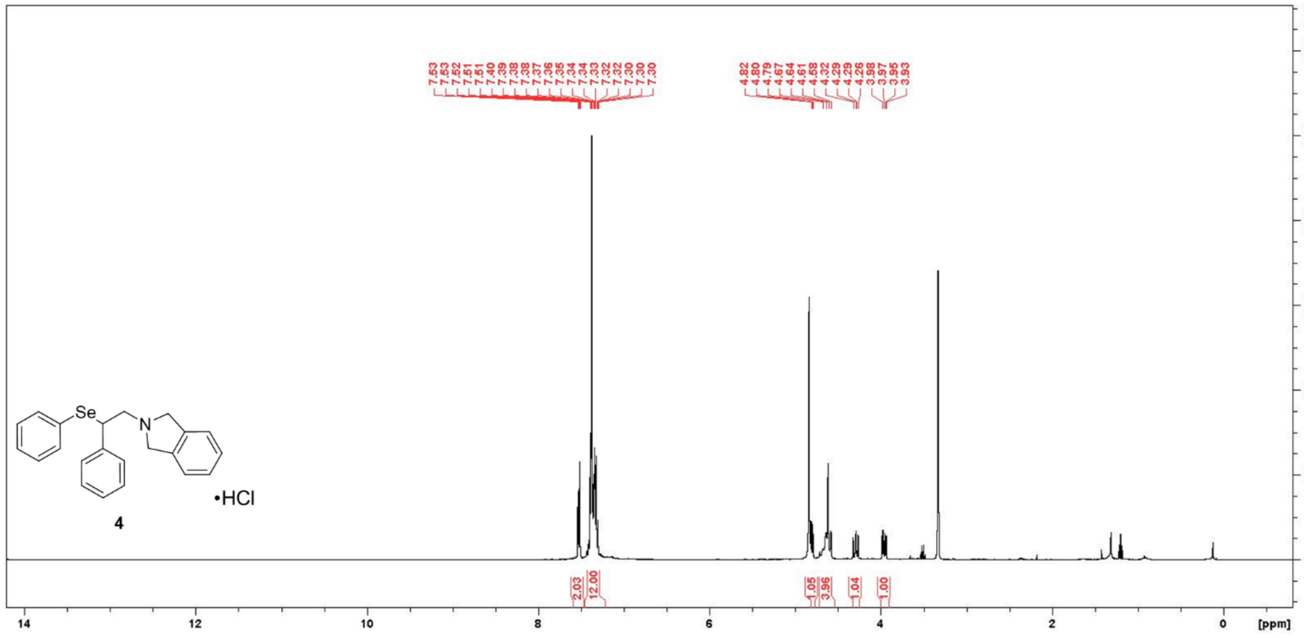

Figure S10. ^{1}H-NMR spectrum of compound **4**.

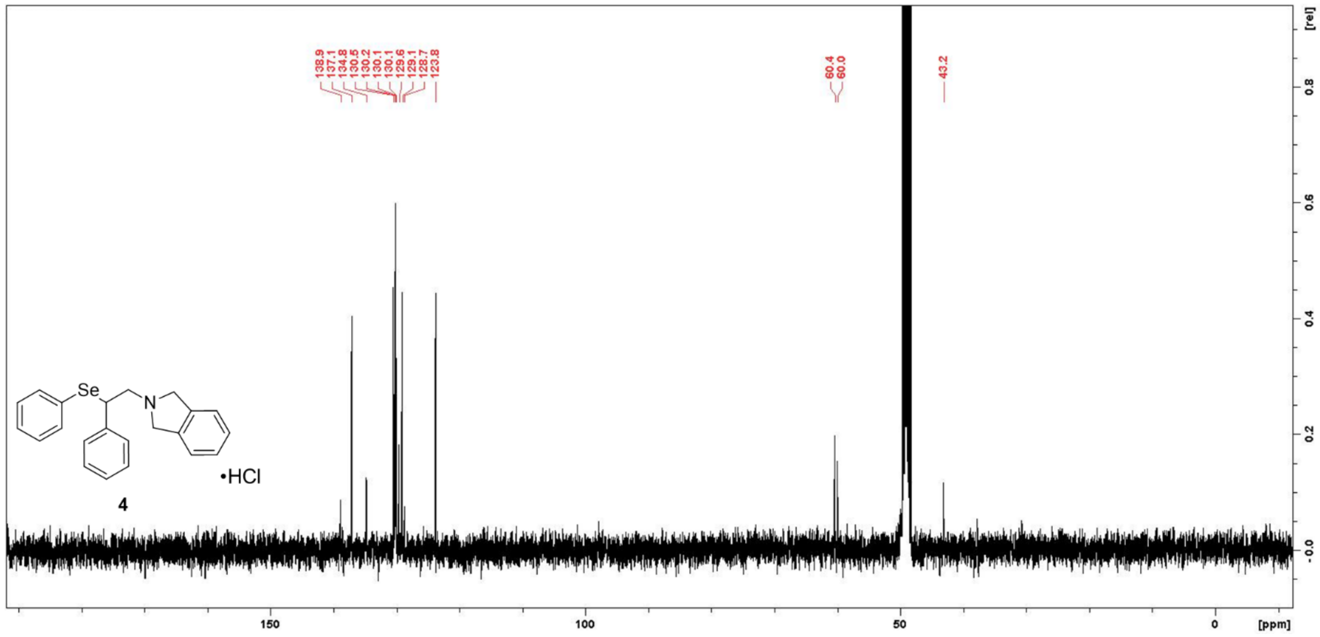

Figure S11. ^{13}C-NMR spectrum of compound **4**.

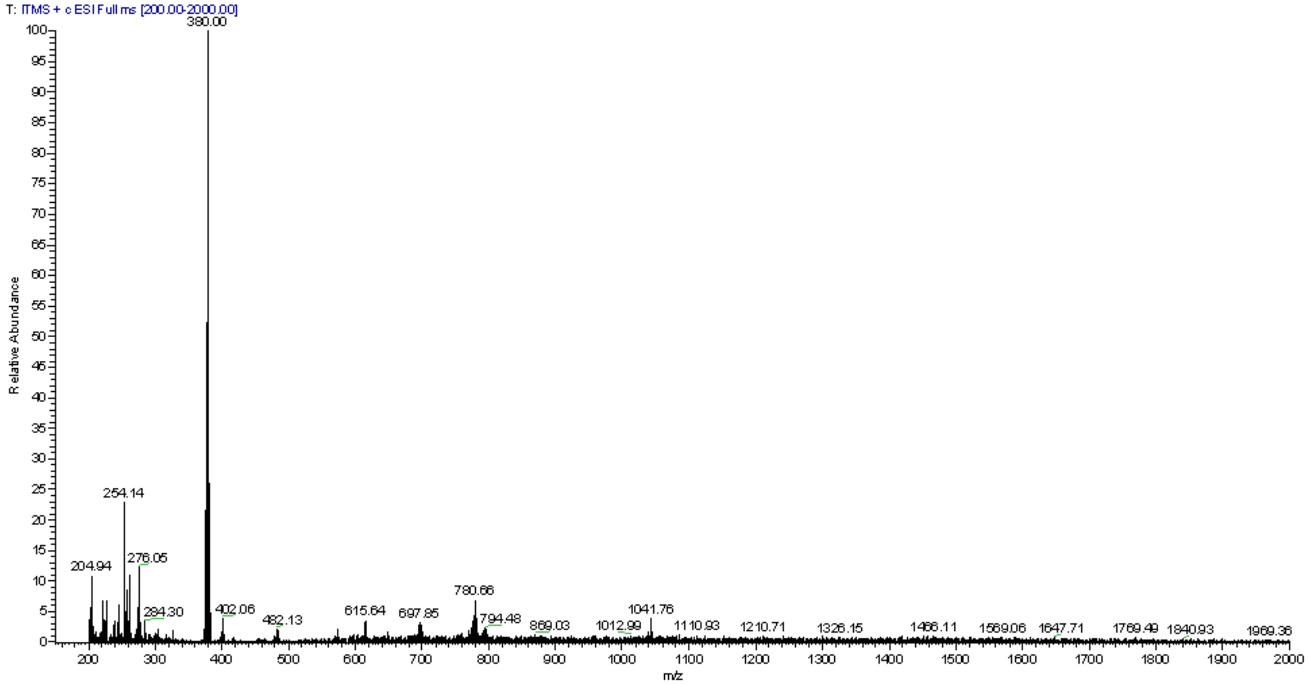

Figure S12. ESI-MS spectrum of compound **4**.

Compound 5

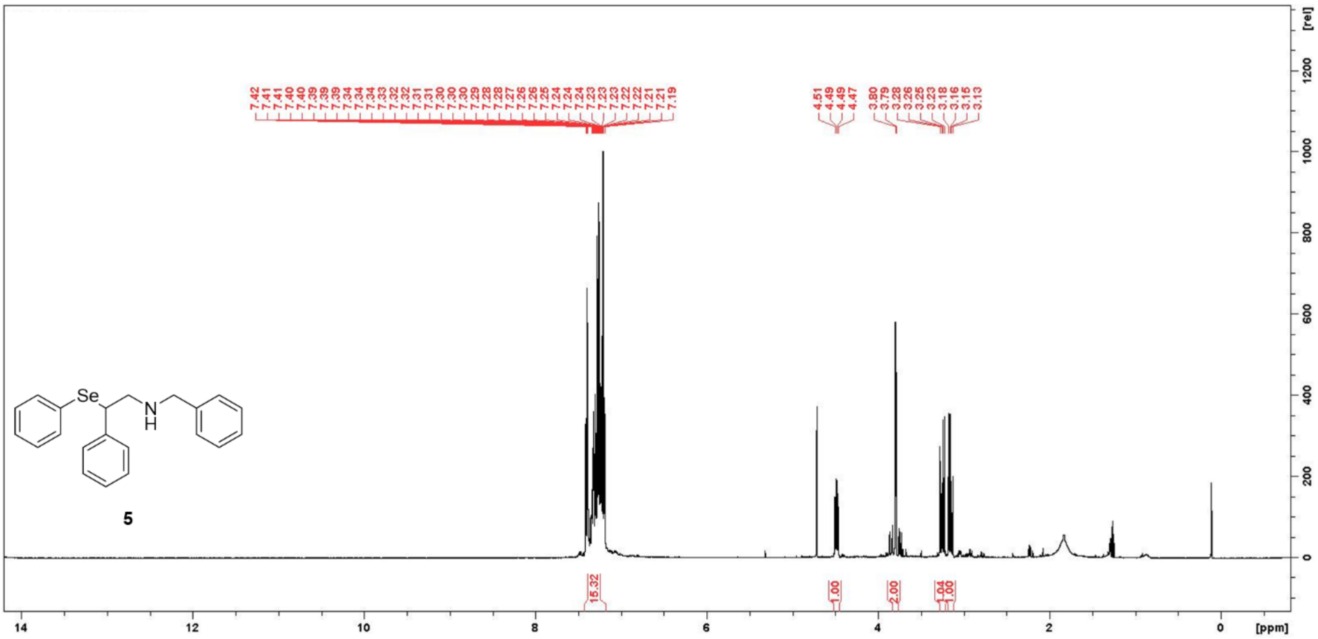

Figure S13. ^{1}H-NMR spectrum of compound **5**.

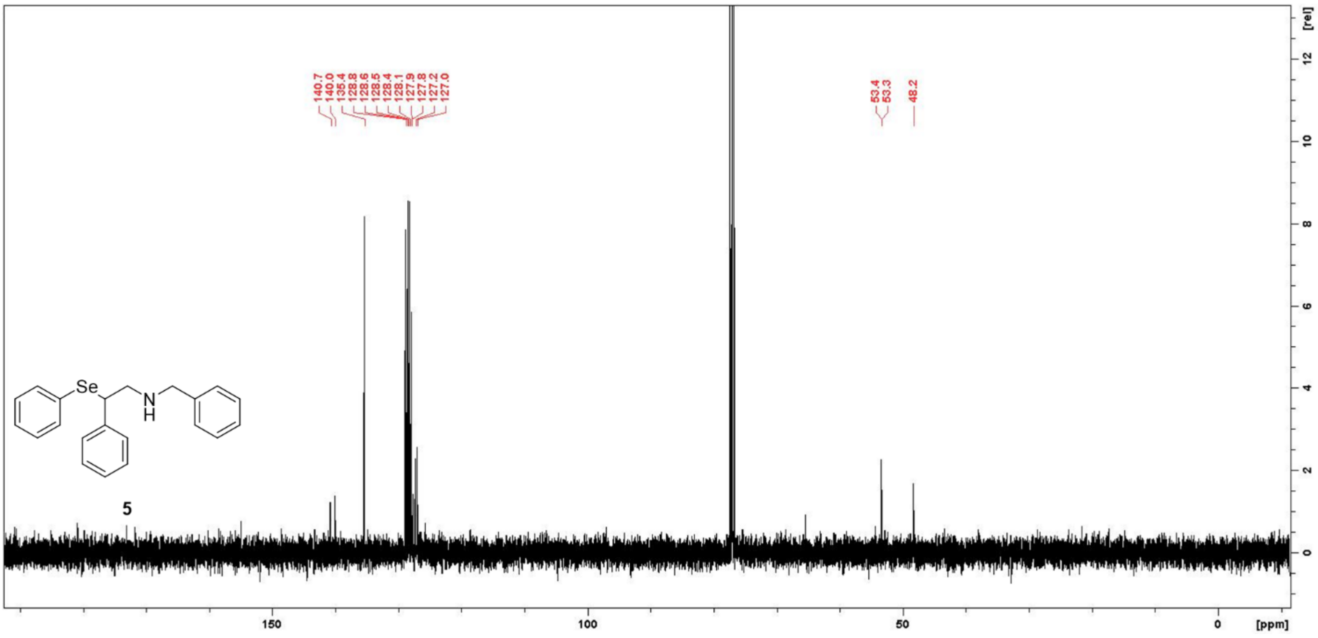

Figure S14. ^{13}C-NMR spectrum of compound **5**.

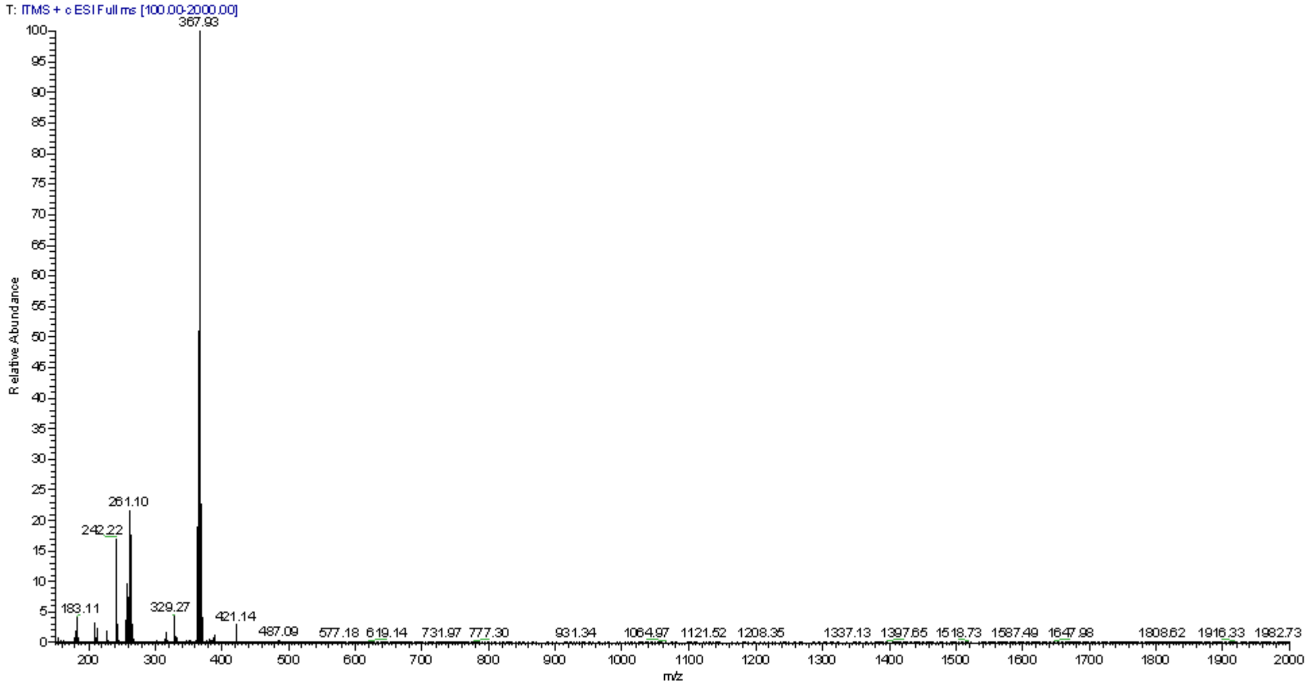

Figure S15. ESI-MS spectrum of compound **5**.

Compound 6

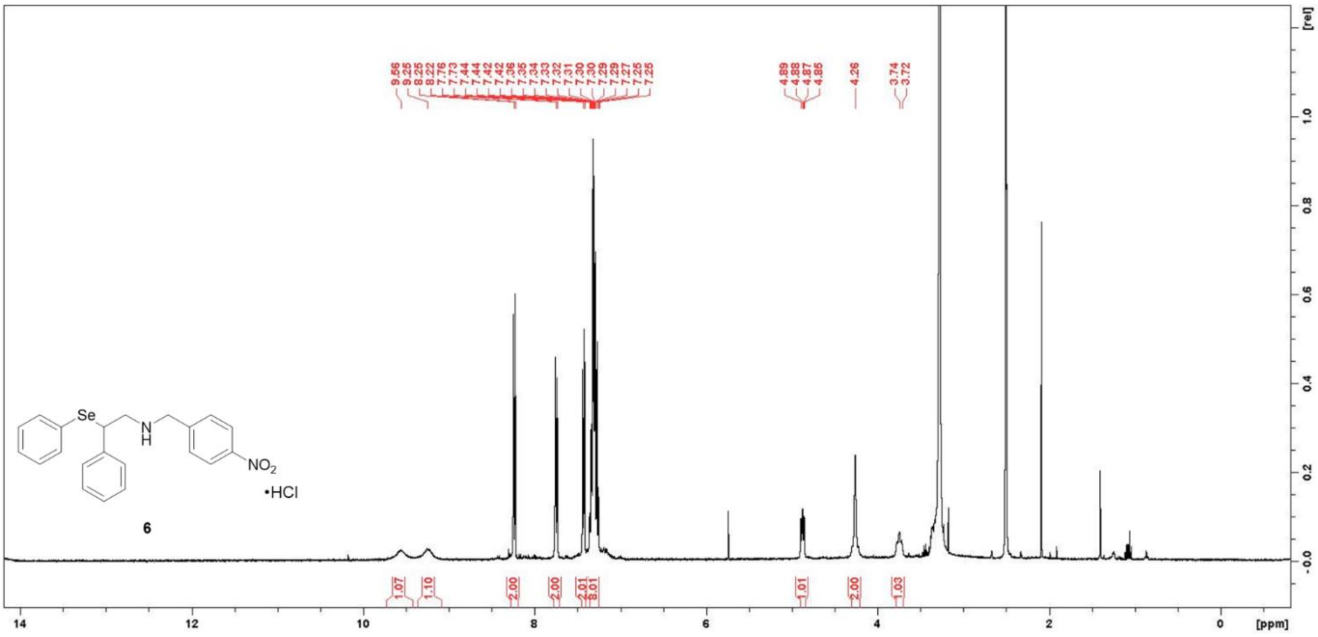

Figure S16. ^{1}H-NMR spectrum of compound **6**.

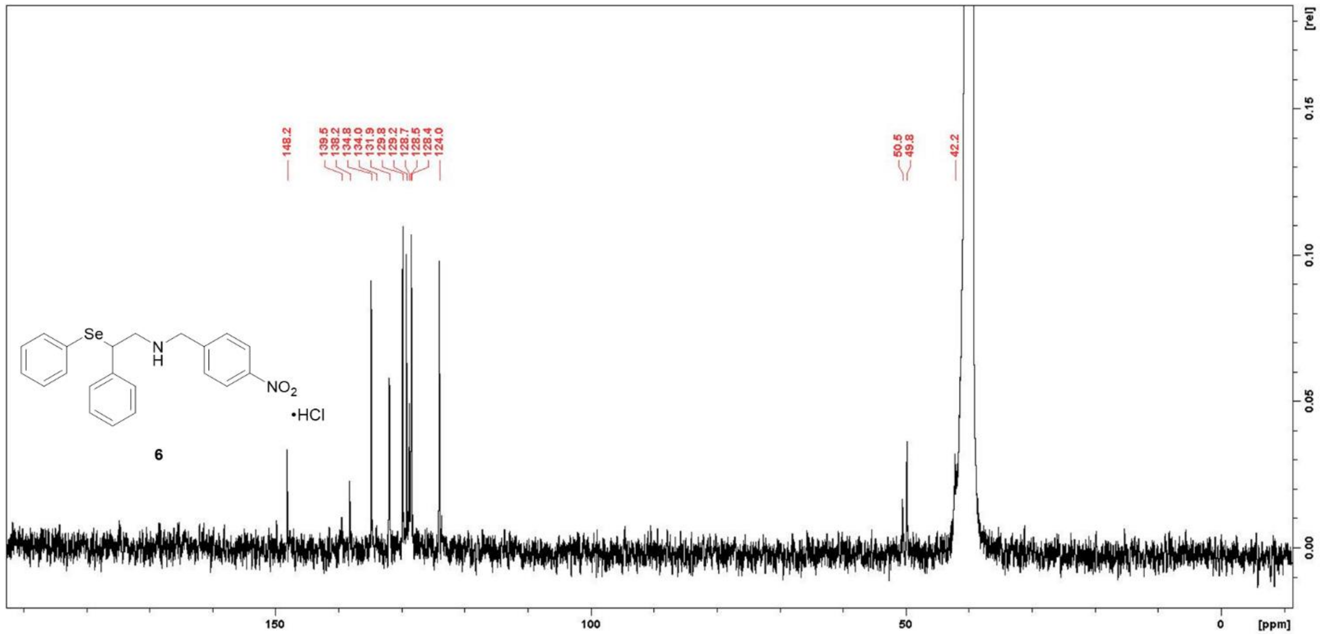

Figure S17. ^{13}C-NMR spectrum of compound **6**.

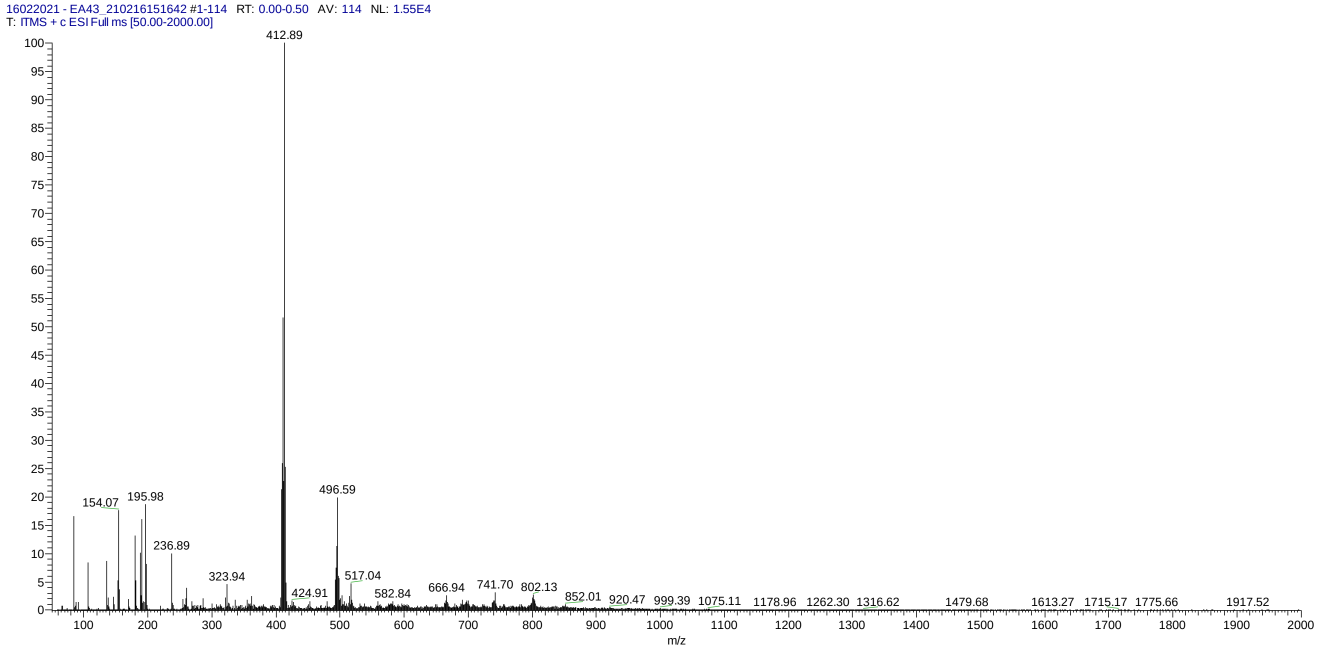

Figure S18. ESI-MS spectrum of compound **6**.

Compound 7

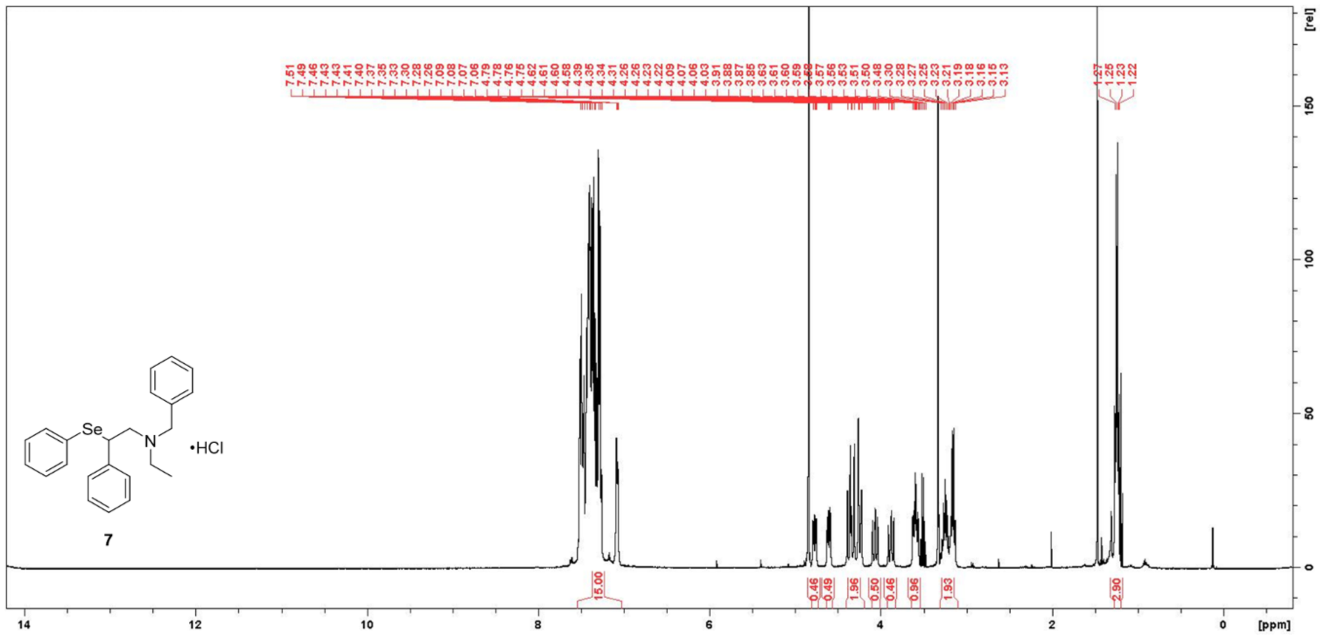

Figure S19. ^{1}H-NMR spectrum of compound **7**.

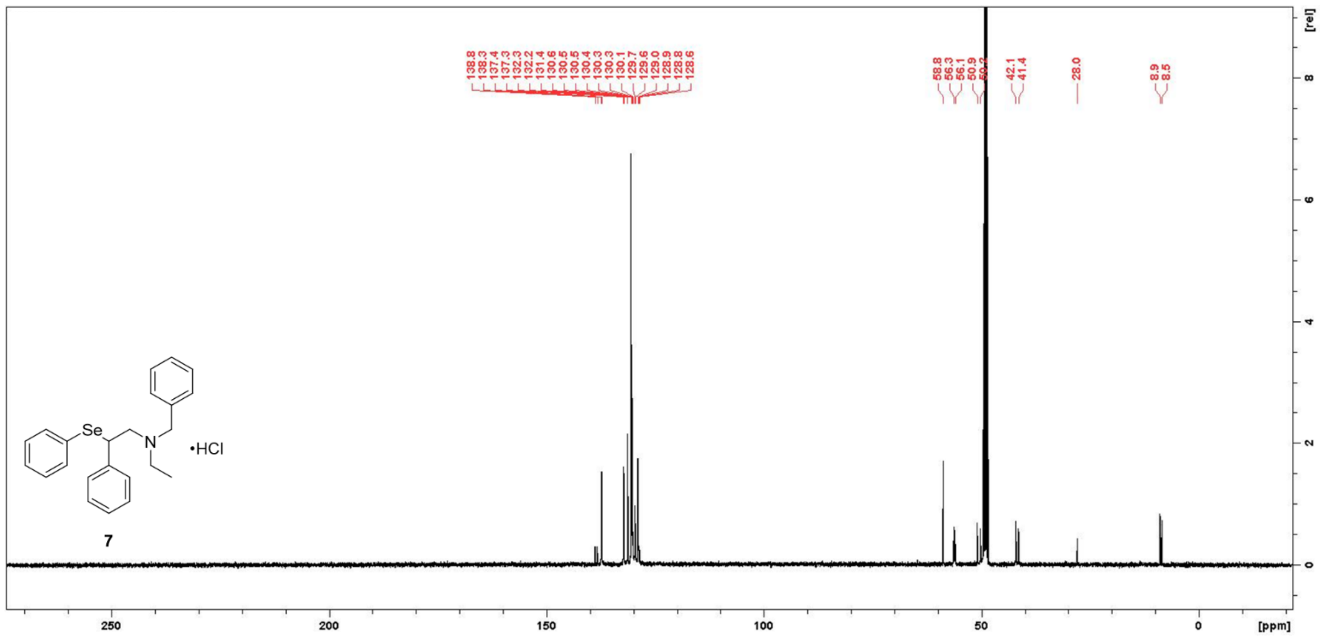

Figure S20. ^{13}C-NMR spectrum of compound **7**.

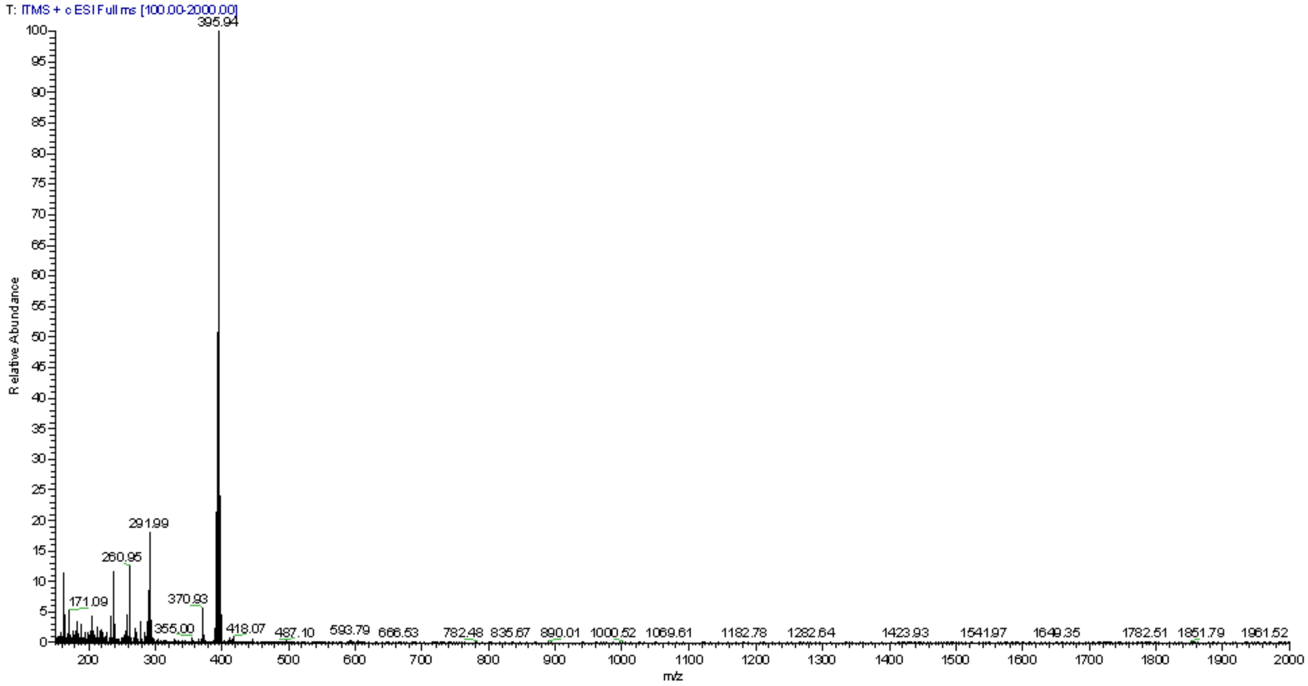

Figure S21. ESI-MS spectrum of compound **7**.

Compound 8

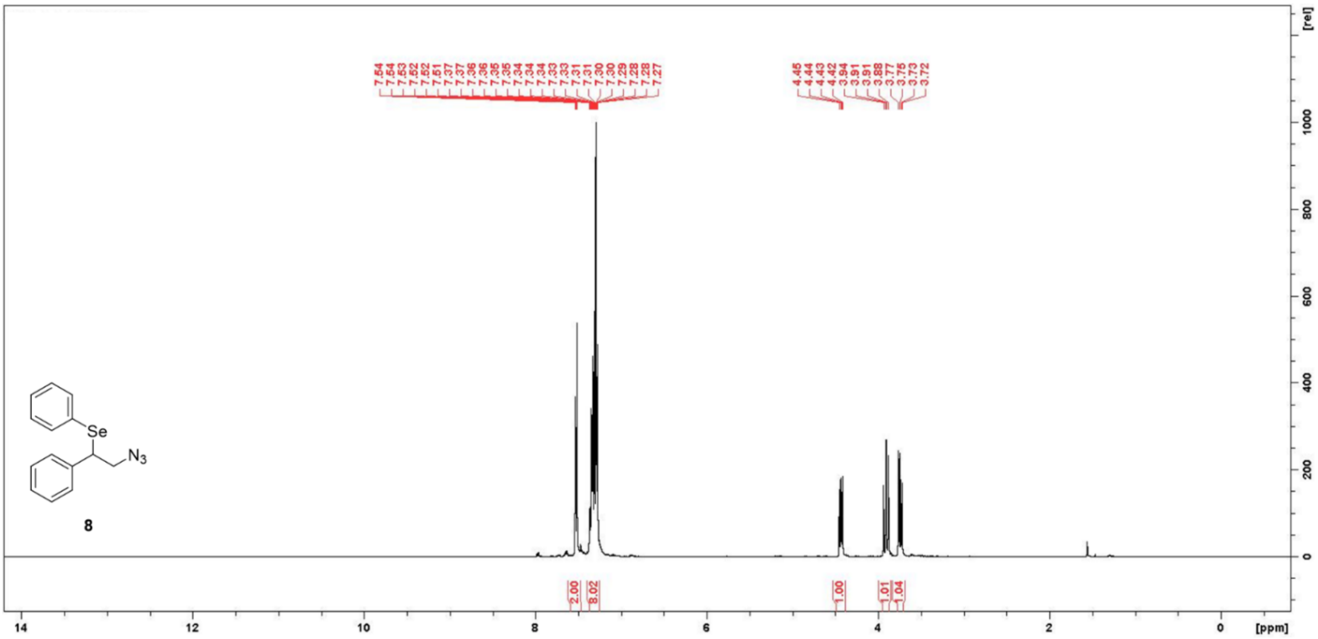

Figure S22. ^{1}H-NMR spectrum of compound **8.**

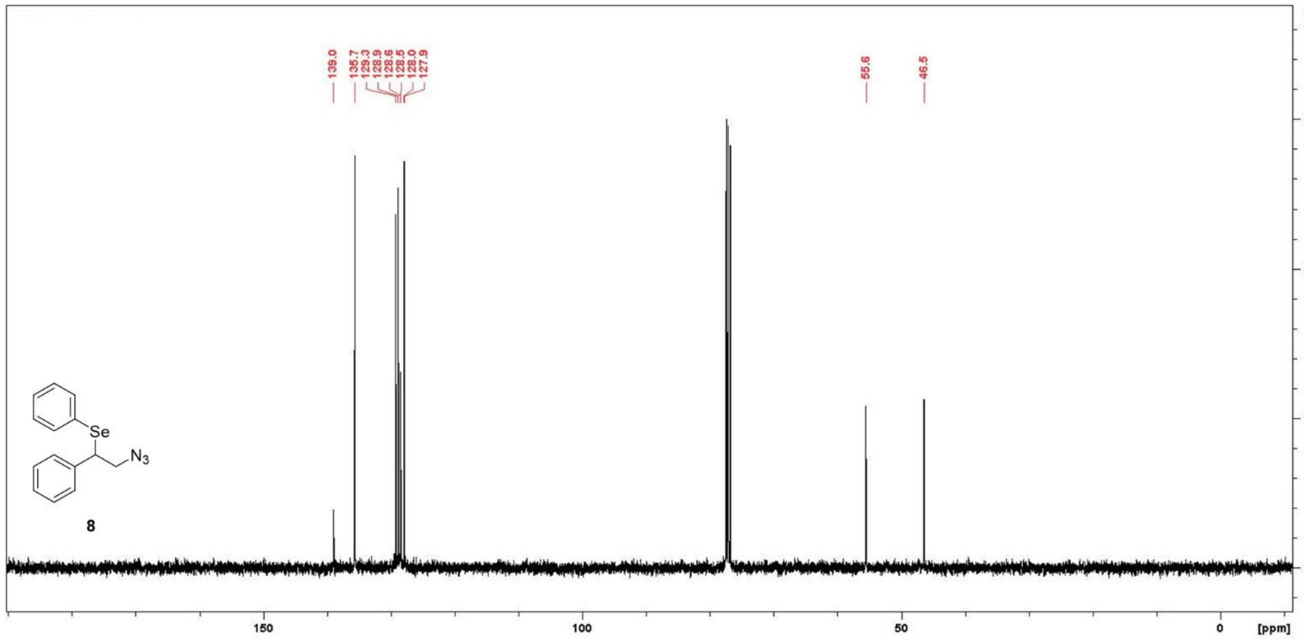

Figure S23. ^{13}C-NMR spectrum of compound **8**.

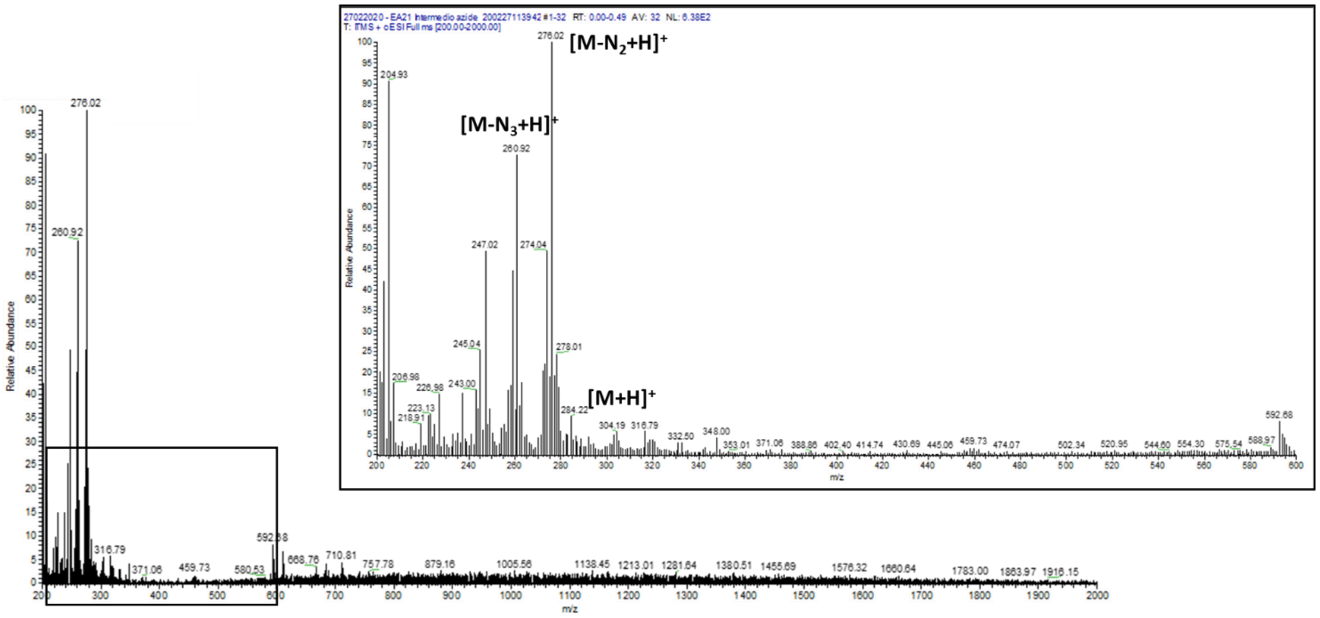

Figure S24. ESI-MS spectrum of compound **8**.

Compound 9

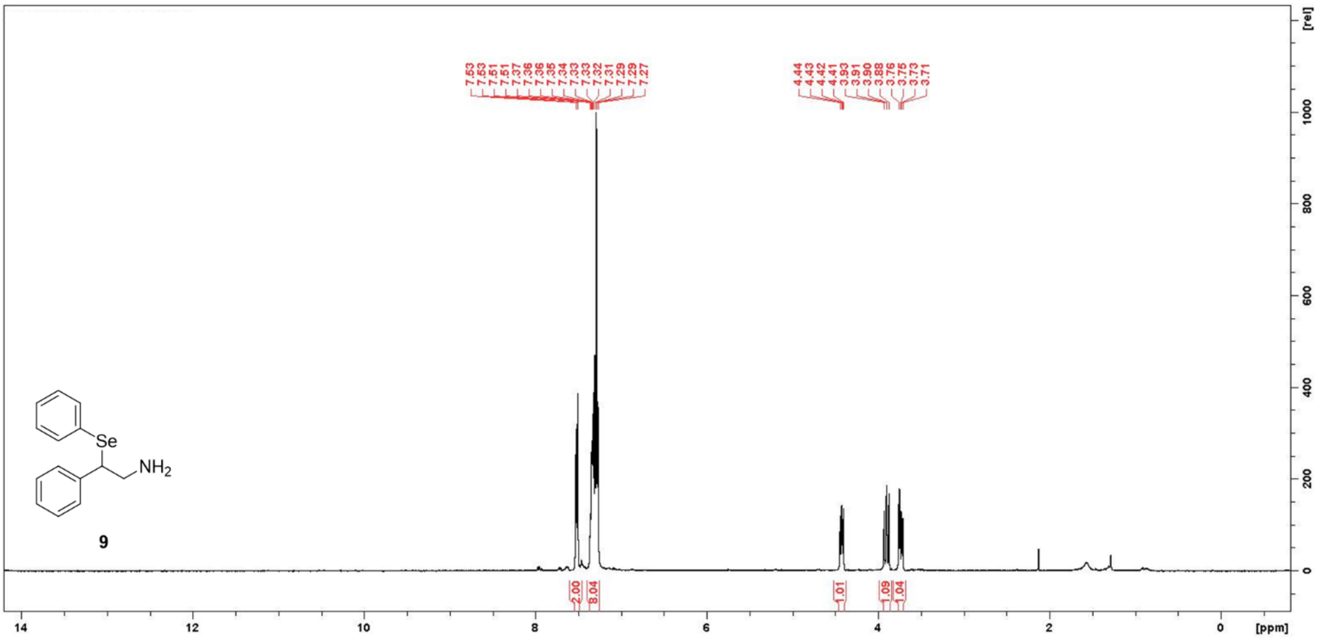

Figure S25. ^{1}H-NMR spectrum of compound **9**.

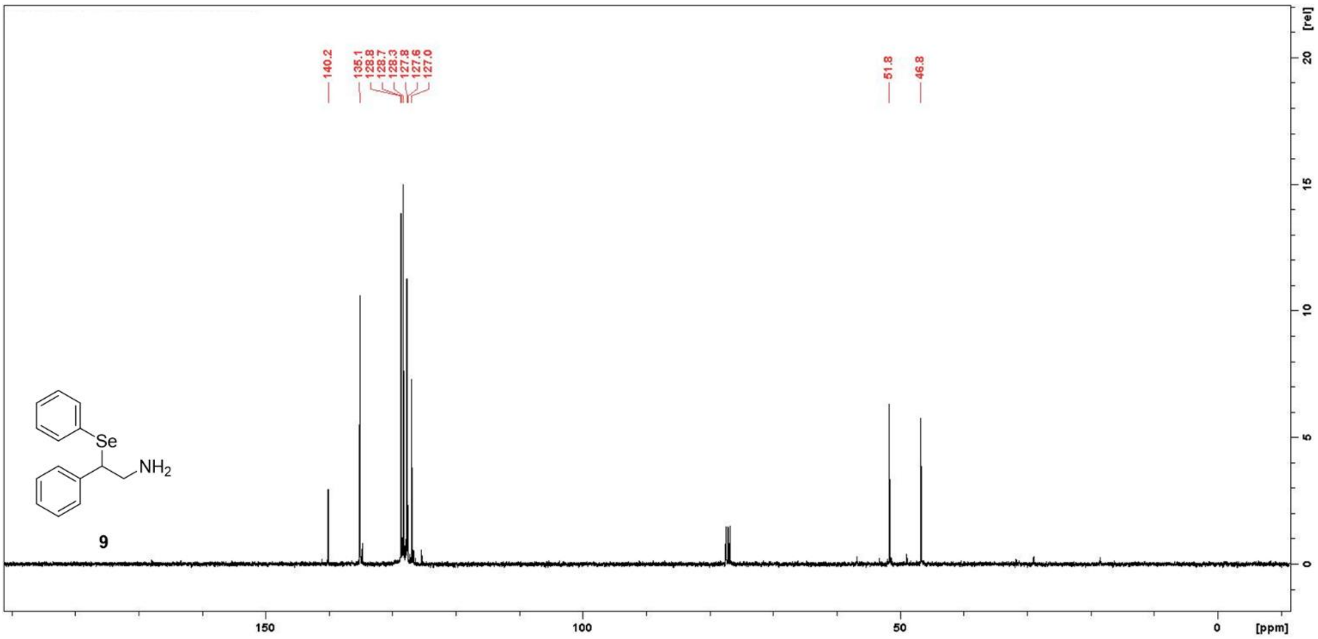

Figure S26. ^{13}C-NMR spectrum of compound **9**.

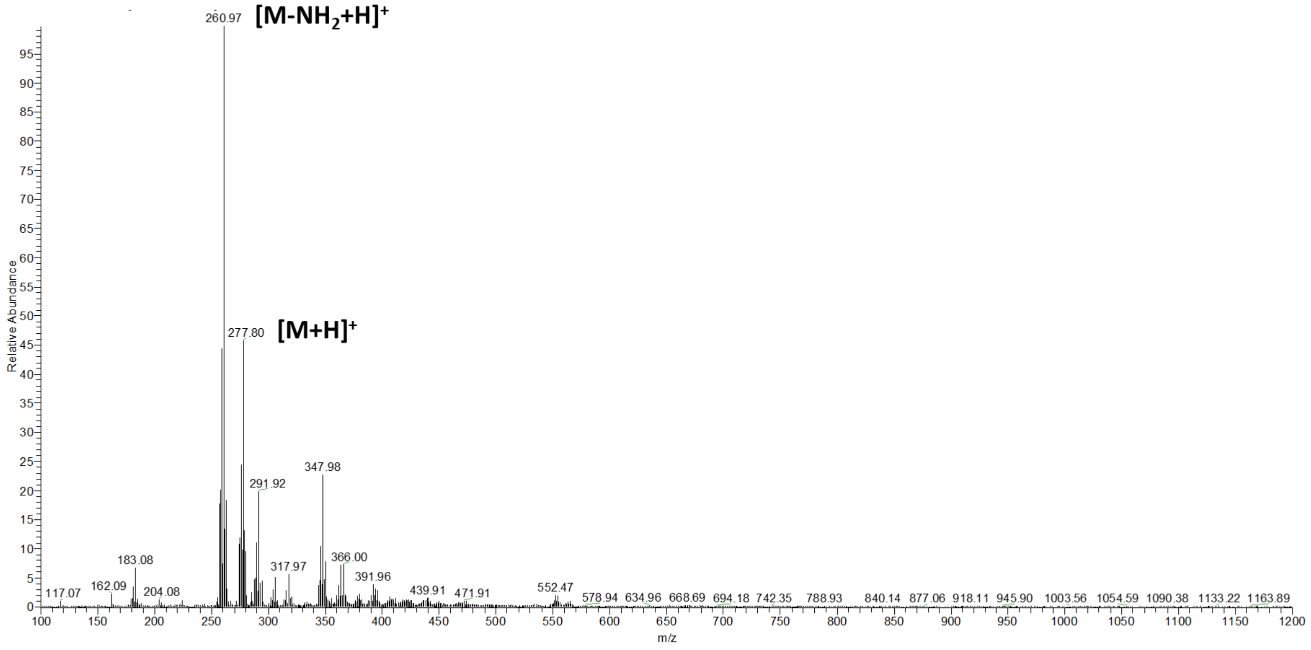

Figure S27. ESI-MS spectrum of compound **9**.

Representative ^{1}H-NMR and ESI-MS spectra of the oxidation study with H_2O_2

Compound 3

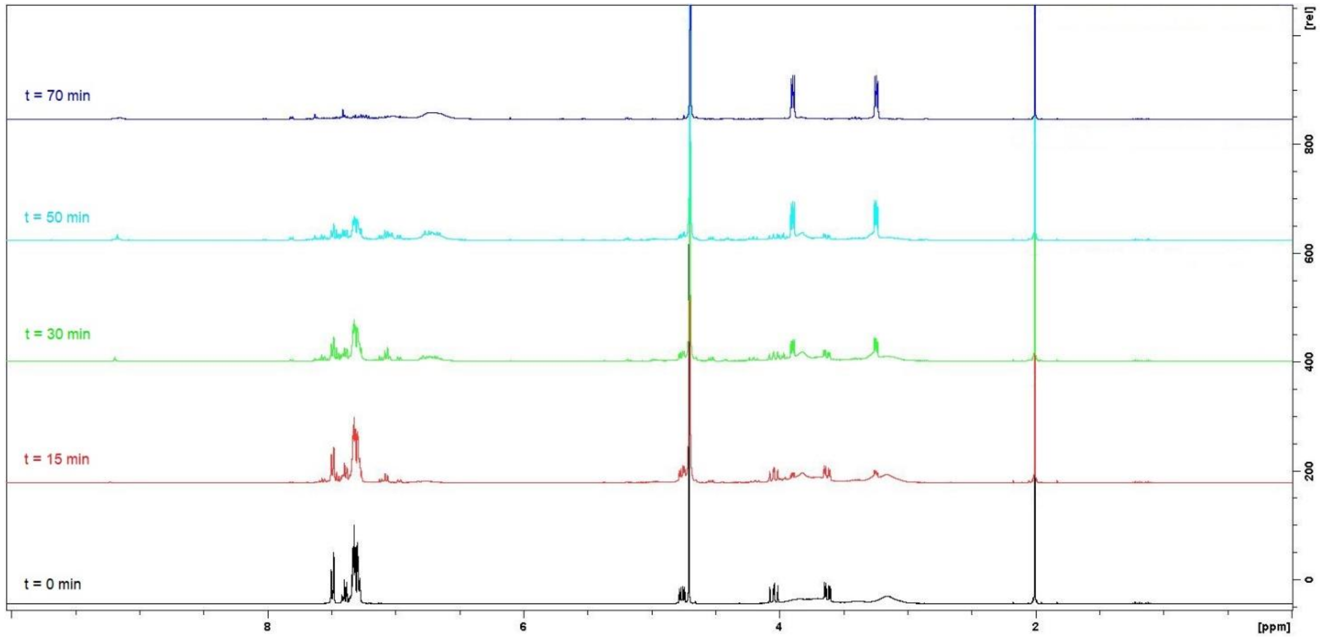

Figure S28. Full ^{1}H-NMR spectra over time of the oxidation of compound **3**.

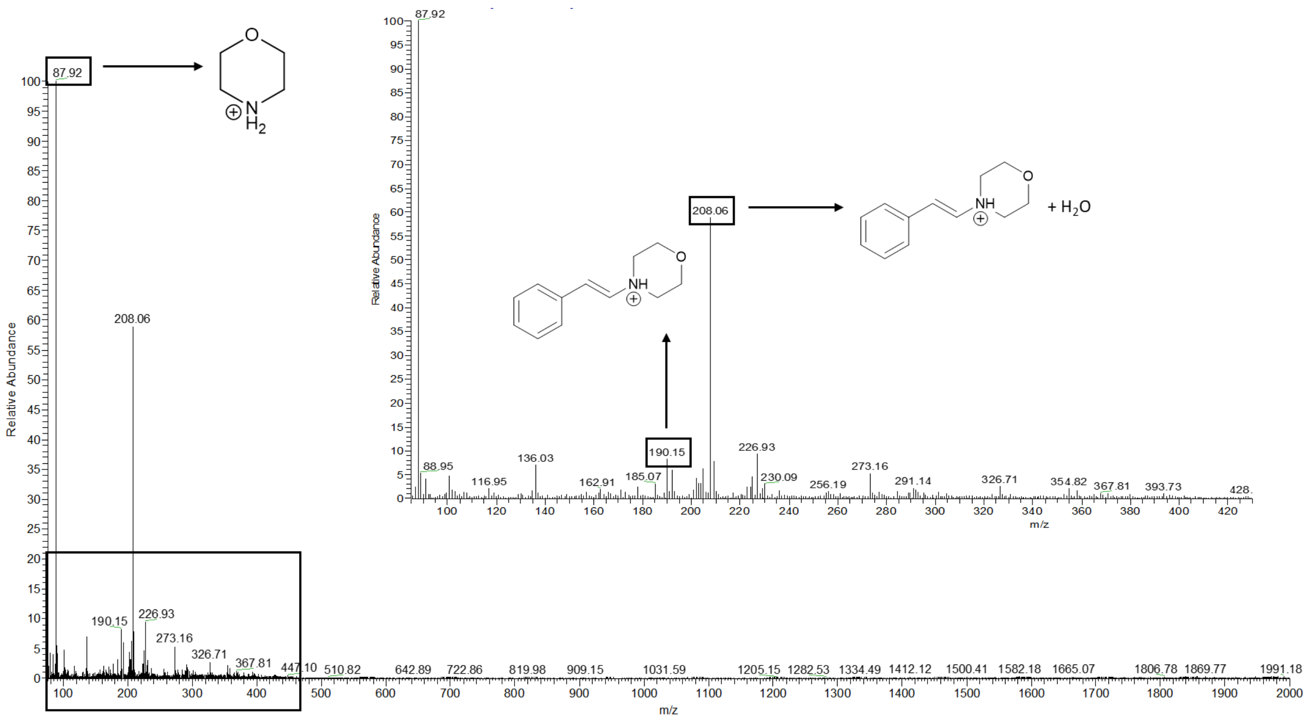

Figure S29. ESI-MS spectrum of the oxidation of compound **3**. The peak at 87.92 m/z corresponds to the final product (*i.e.* morpholine), the peaks at 190.15 and 208.06 m/z correspond respectively to the enamine and its adduct with water.

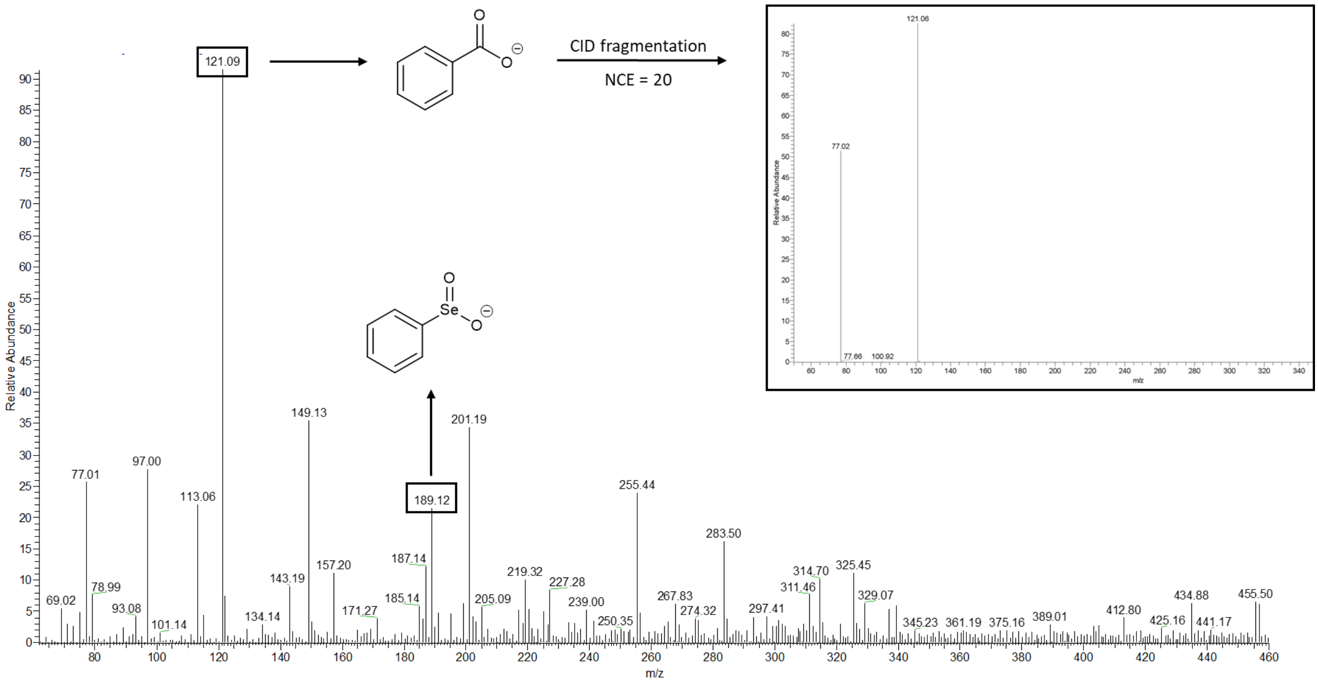

Figure S30. Negative ionization ESI-MS spectra of the oxidation of compound **3**. The peak at 121.09 m/z correspond to benzoic acid (as confirmed by the Collision Induced Dissociation (CID) experiment at Normalized Collision Energy (NCE) = 20), the peak at 189.12 corresponds to the seleninic acid.

Compound 1

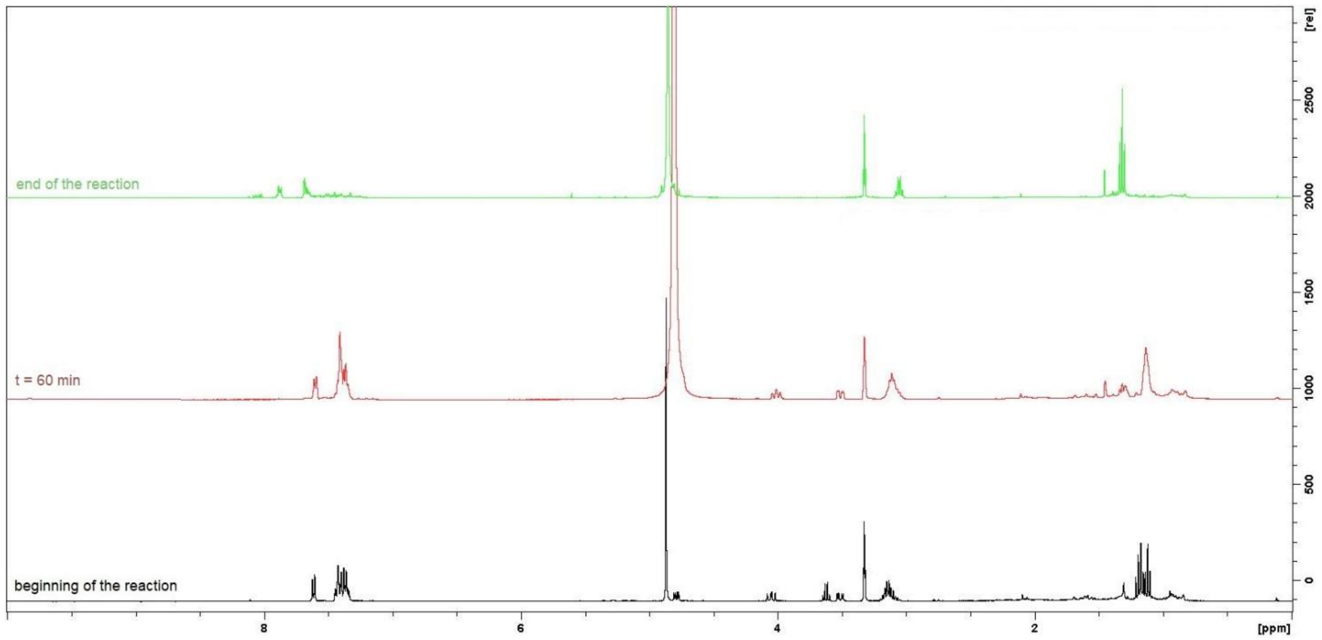

Figure S31. ^{1}H-NMR spectra of compound **1** oxidation over time (• before the addition of H_2O_2, • after 60 minutes from the addition of H_2O_2, • overnight reaction).

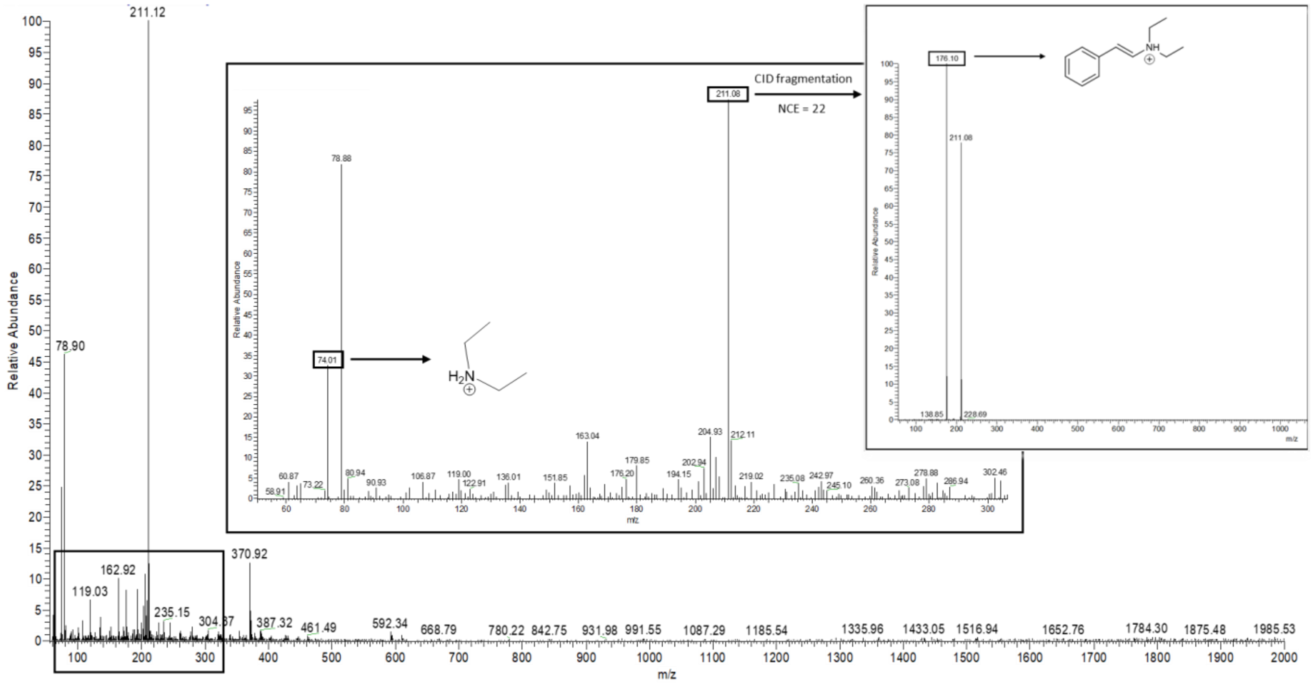

Figure S32. ESI-MS spectra of the oxidation after 60 minutes of compound **1**. Spectrum was acquired after dilution of the NMR sample (MeOD/D_2O, 85/15) in MeOH. The peak at 74.01 m/z corresponds to the final product (*i.e.* diethylamine), the peak at 211.01 m/z corresponds to the deuterated methanol adduct of the enamine as confirmed by the Collision Induced Dissociation (CID) spectrum registered at Normalized Collision Energy (NCE) = 22.

Compound 5

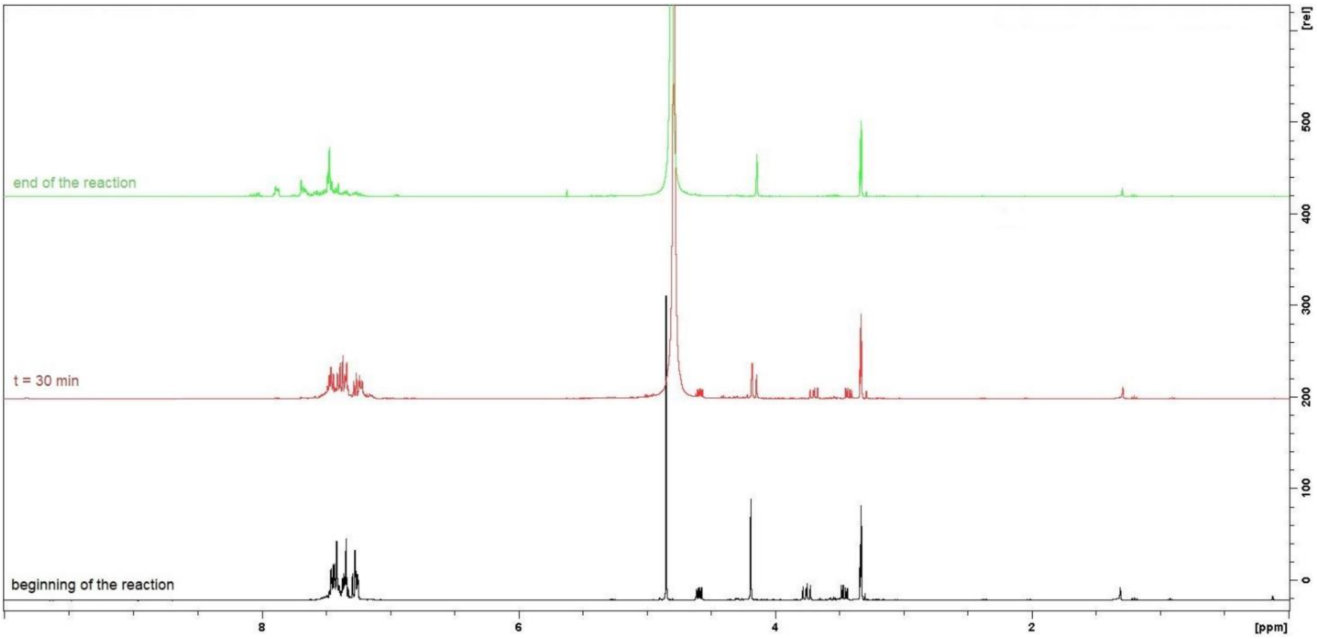

Figure S33. ^{1}H-NMR spectra of compound **5** oxidation over time (• before the addition of H_2O_2, • after 30 minutes from the addition of H_2O_2, • overnight reaction).

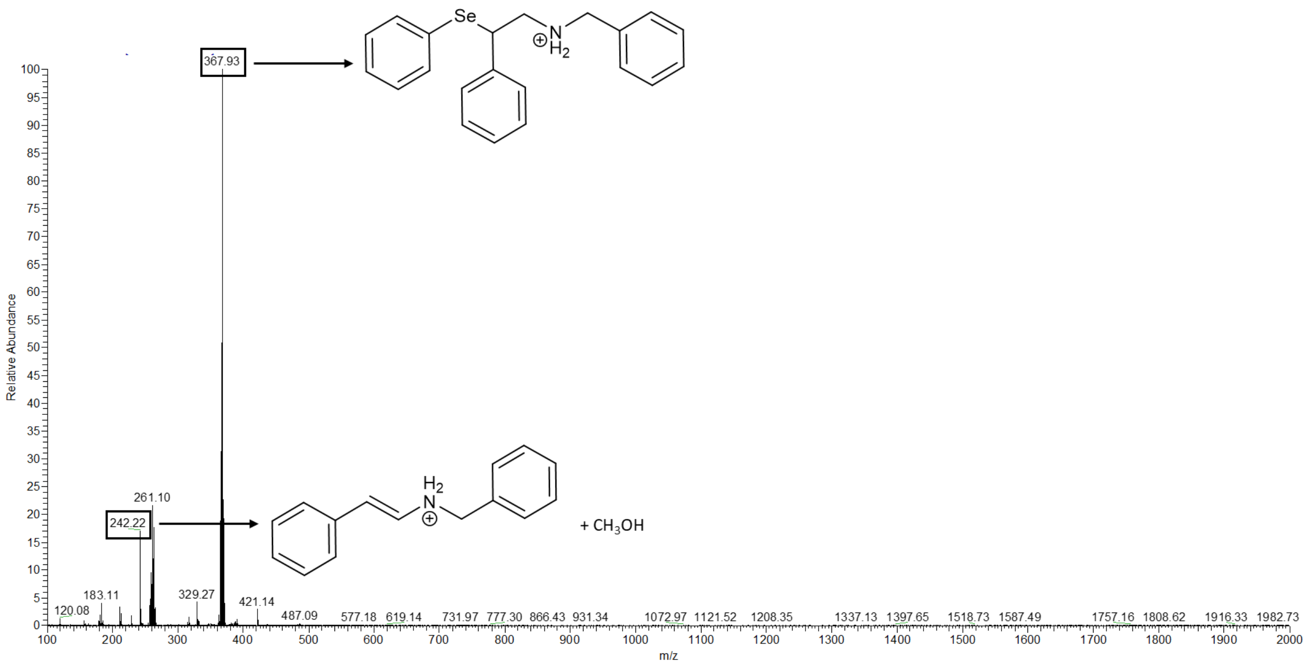

Figure S34. ESI-MS spectra of the oxidation after 30 minutes of compound **5**. Spectrum was acquired after dilution of the NMR sample (MeOD/D_2O, 85/15) in MeOH. The peak at 242.22 m/z corresponds to the methanol adduct of the enamine, the peak at 367.93 corresponds to the starting material.

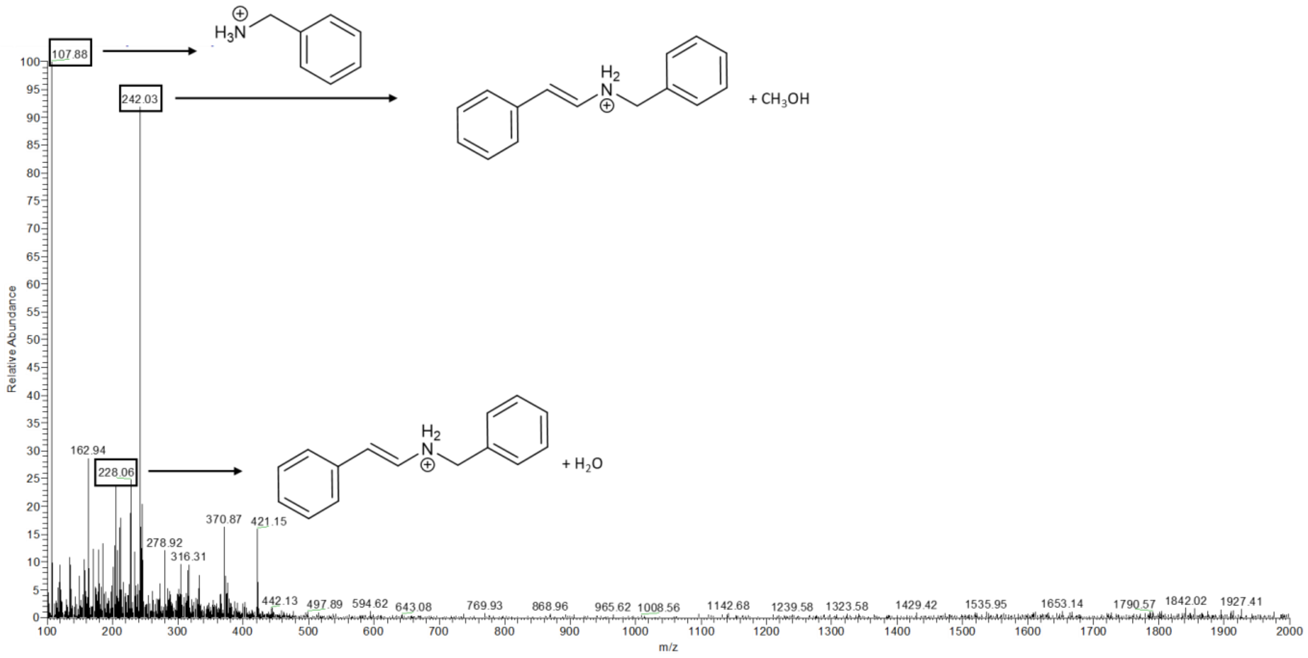

Figure S35. ESI-MS spectra of the oxidation after 60 minutes of compound **5**. Spectrum was acquired after dilution of the NMR sample (MeOD/D_2O, 85/15) in MeOH. The peak at 107.88 m/z corresponds to the final product (*i.e.* benzylamine), the peaks at 228.06 and 242.03 m/z correspond respectively to the methanol and water adduct of the enamine.

Compound 7

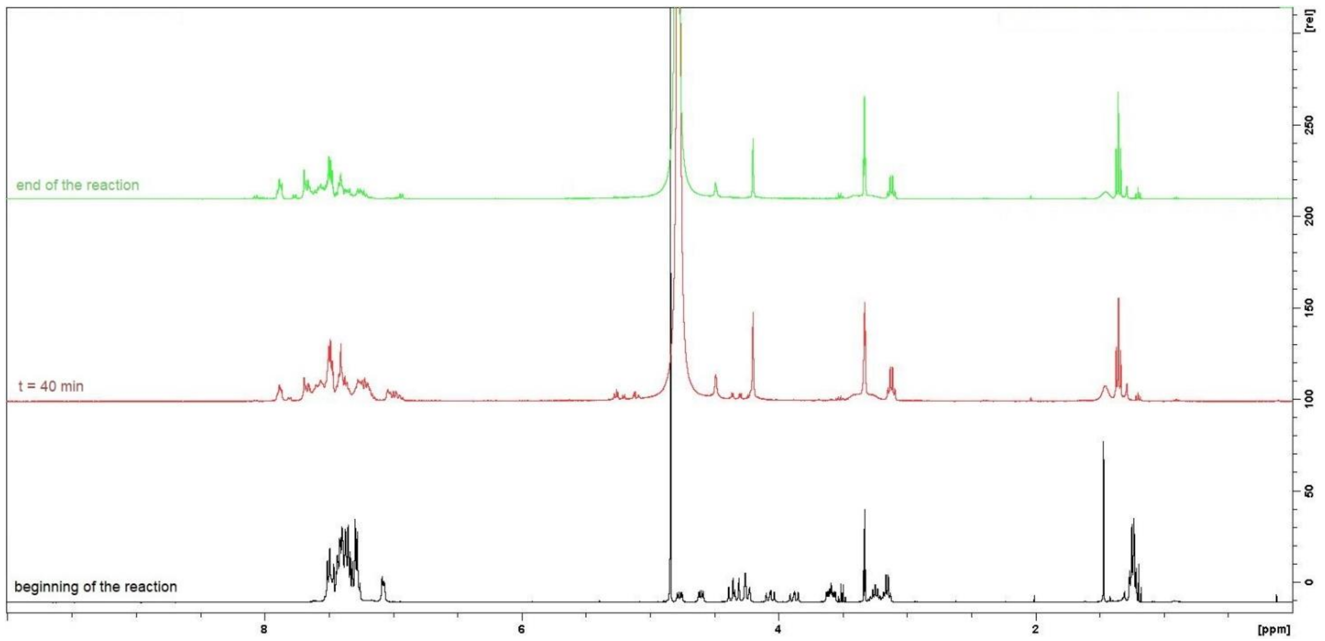

Figure S36. ^{1}H-NMR spectra of compound **7** oxidation over time (• before the addition of H_2O_2, • after 30 minutes from the addition of H_2O_2, • overnight reaction).

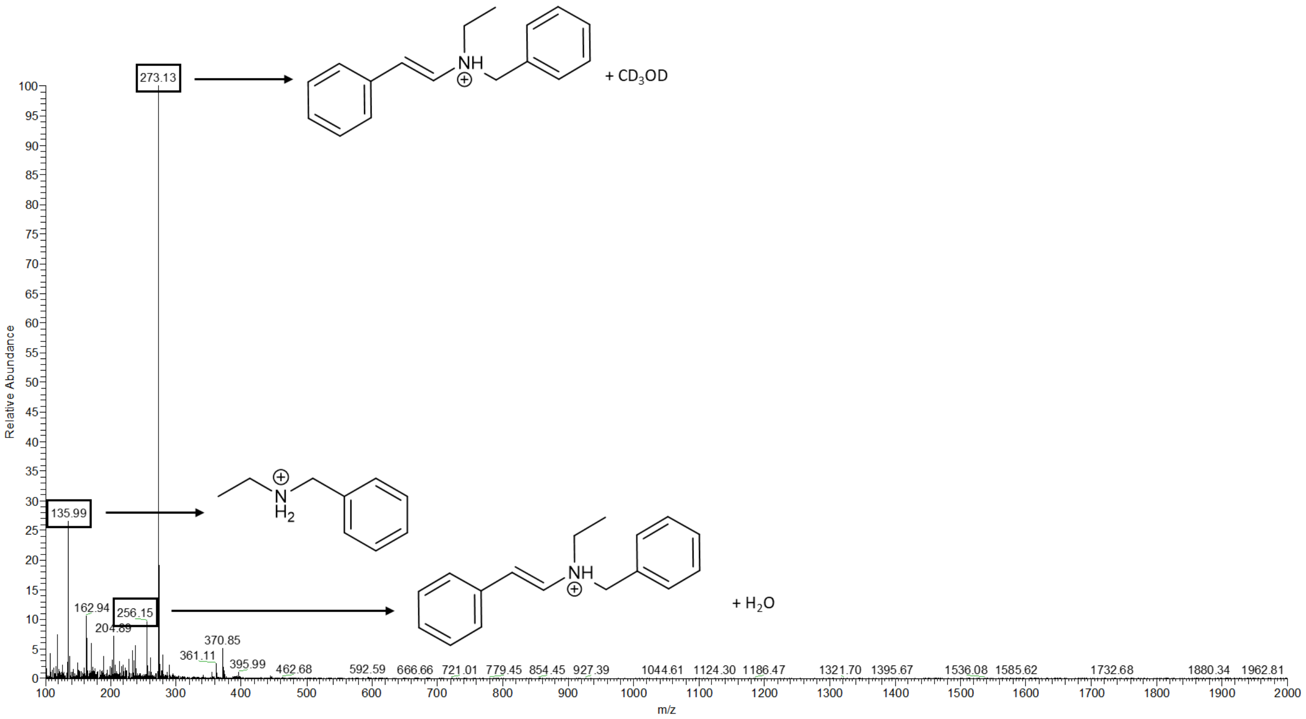

Figure S37. ESI-MS spectra of the oxidation of compound **7**. Spectrum was acquired after dilution of the NMR sample (MeOD/D_2O, 85/15) in MeOH. The peak at 135.99 m/z corresponds to the final product (*i.e.* N-ethylbenzylamine), the peaks at 256.15 and 273.13 m/z correspond respectively to the deuterated methanol and water adduct of the enamine.

^{1}H-NMR, ^{13}C-NMR and ESI-MS spectra of the isolated compounds obtained by oxidation with H_2O_2

Diethylamine•HCl

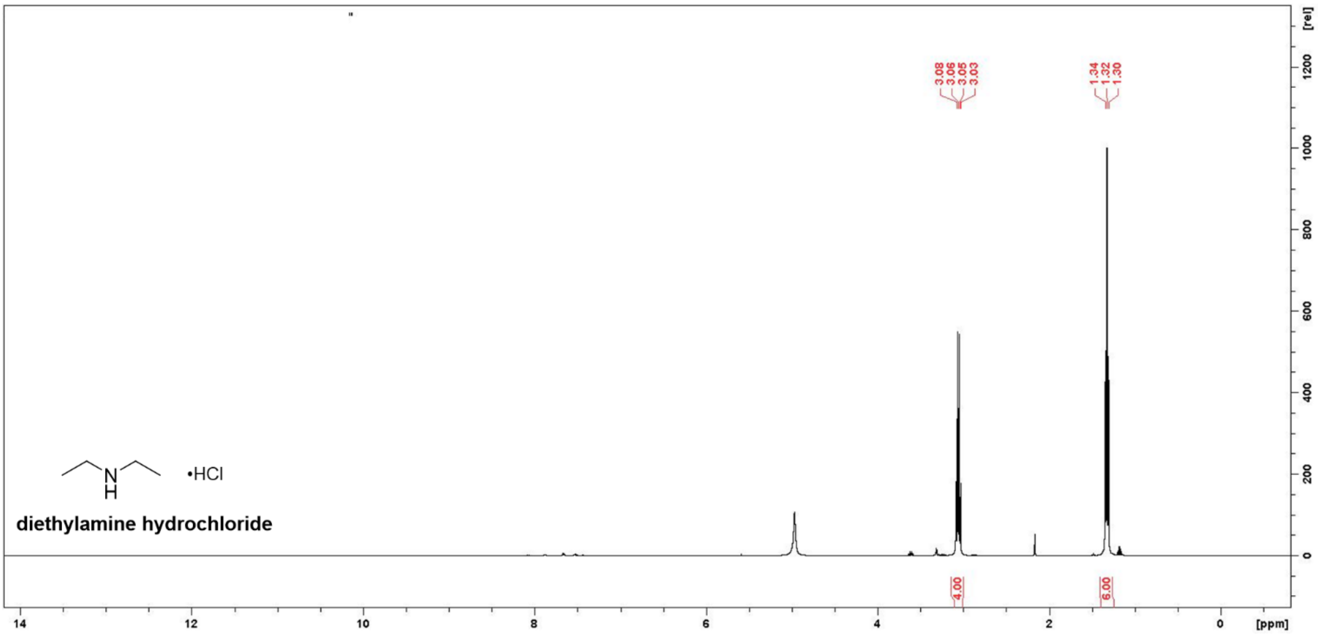

Figure S38. ^{1}H-NMR spectrum of the final product of the oxidation of compound **1**, *i.e.* diethylamine hydrochloride.

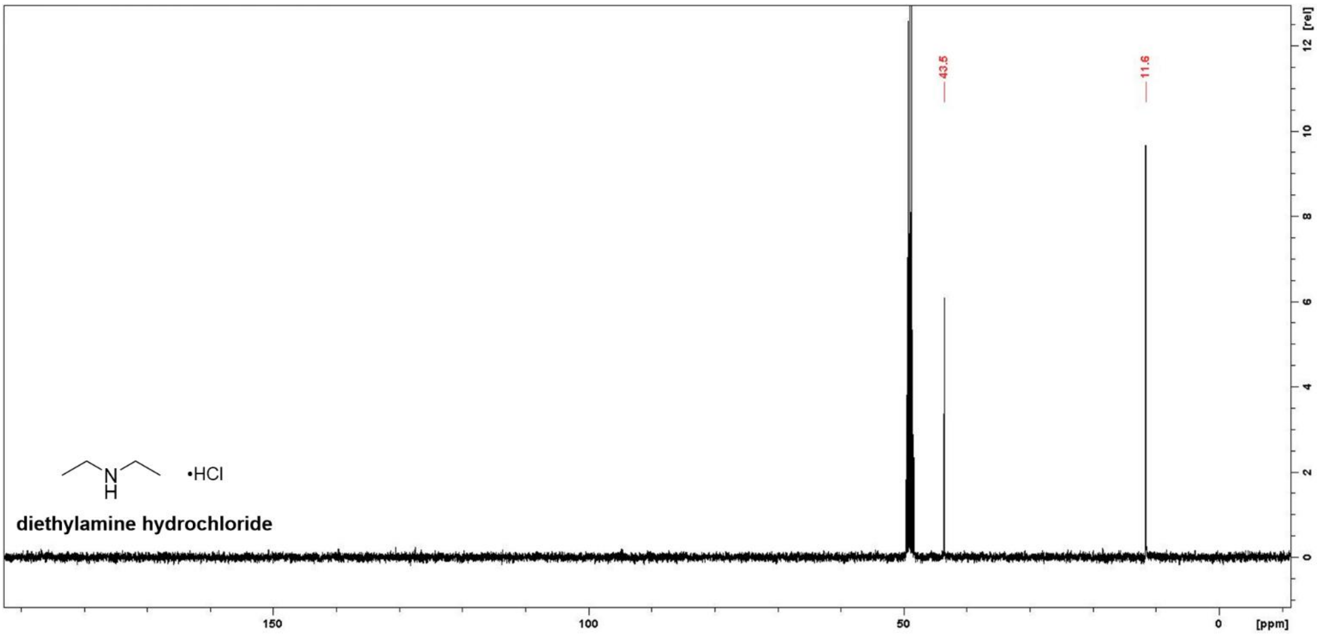

Figure S39. ^{13}C-NMR spectrum of the final product of the oxidation of compound **1**, *i.e.* diethylamine hydrochloride.

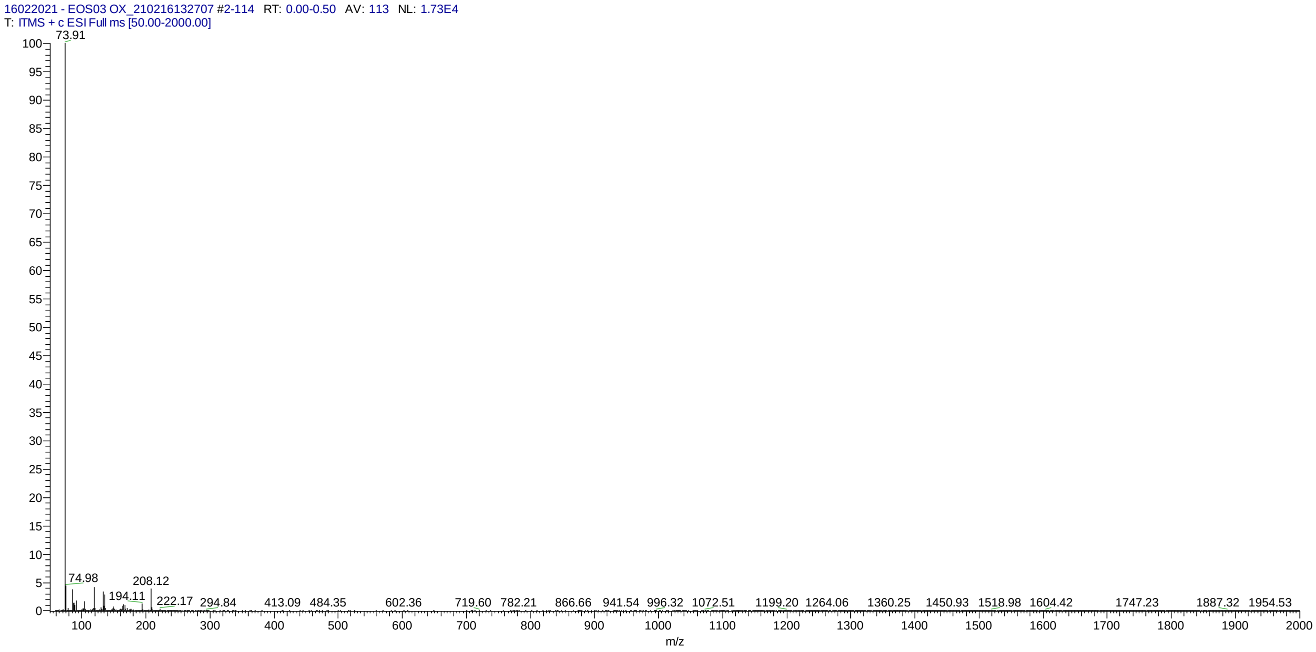

Figure S40. ESI-MS spectrum of the final product of the oxidation of compound **1**, *i.e.* diethylamine hydrochloride.

Dibenzylamine•HCl

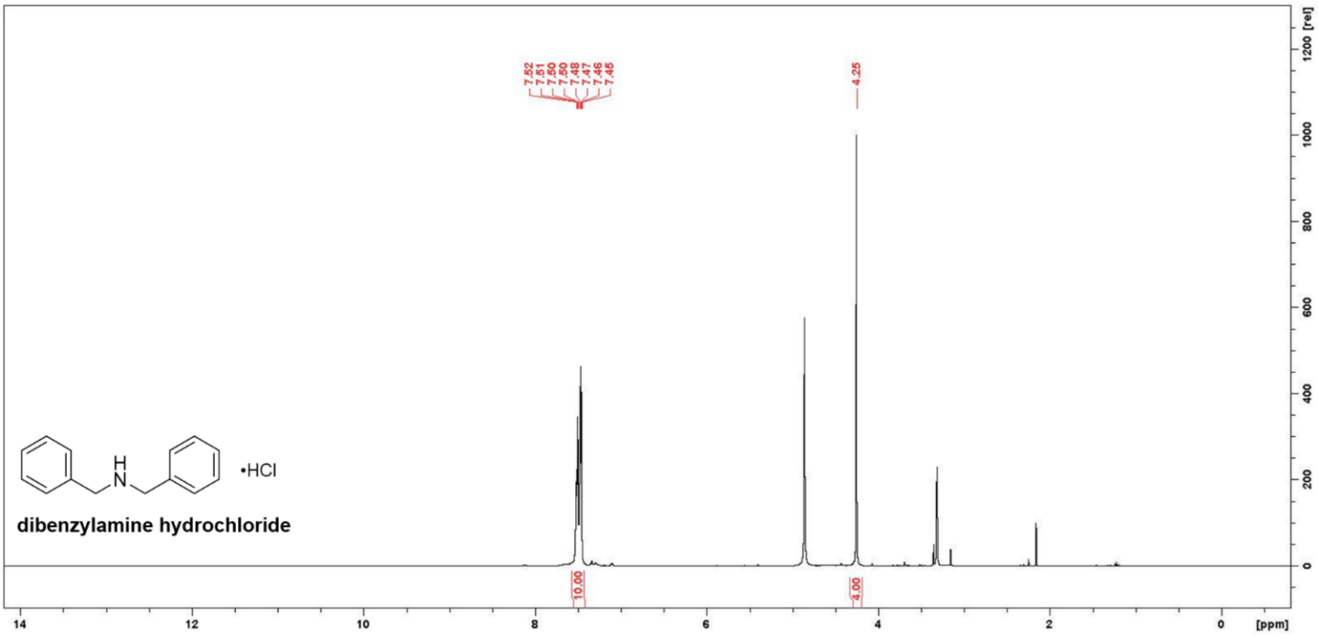

Figure S41. ^{1}H-NMR spectrum of the final product of the oxidation of compound **2**, *i.e.* dibenzylamine hydrochloride.

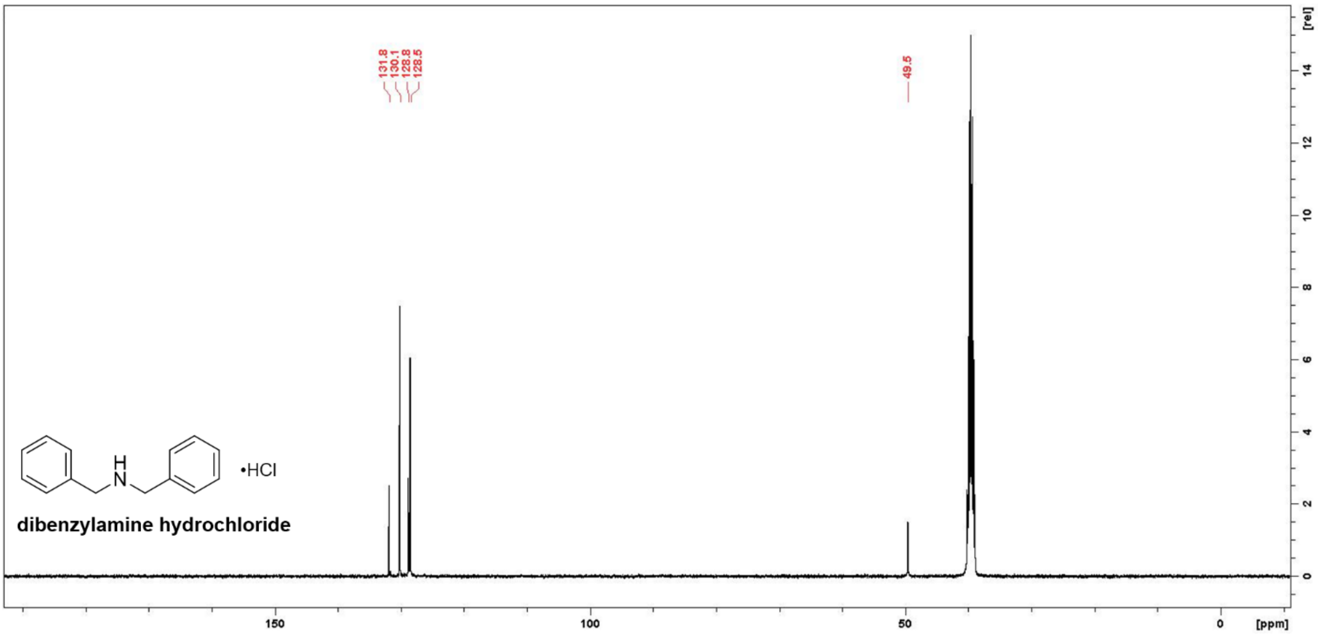

Figure S42. ^{13}C-NMR spectrum of the final product of the oxidation of compound **2**, *i.e.* dibenzylamine hydrochloride.

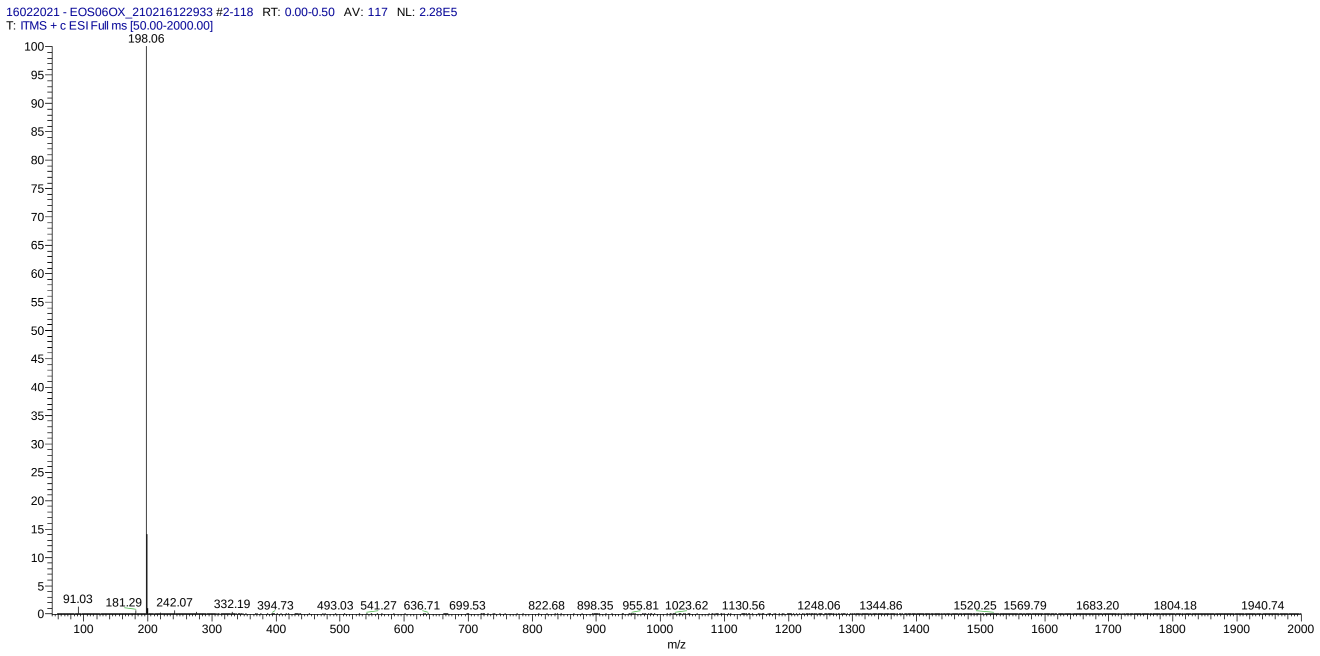

Figure S43. ESI-MS spectrum of the final product of the oxidation of compound **2**, *i.e.* dibenzylamine hydrochloride.

Morpholine•HCl

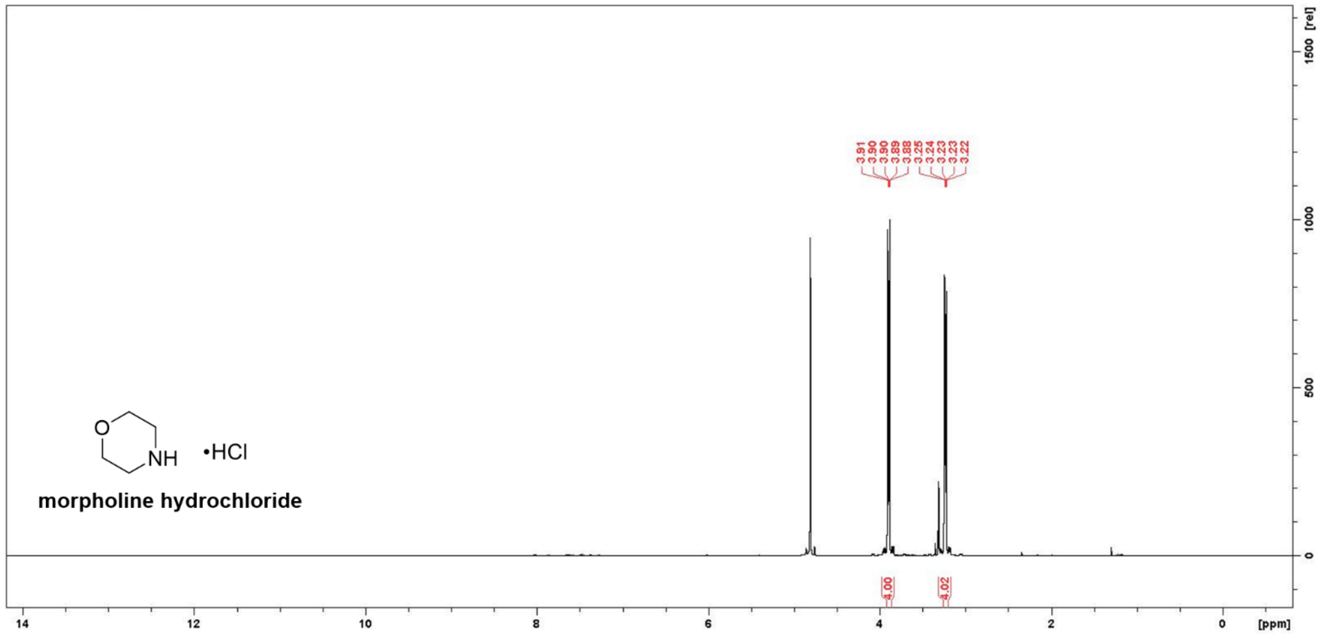

Figure S44. ^{1}H-NMR spectrum of the final product of the oxidation of compound **3,** *i.e.* morpholine hydrochloride.

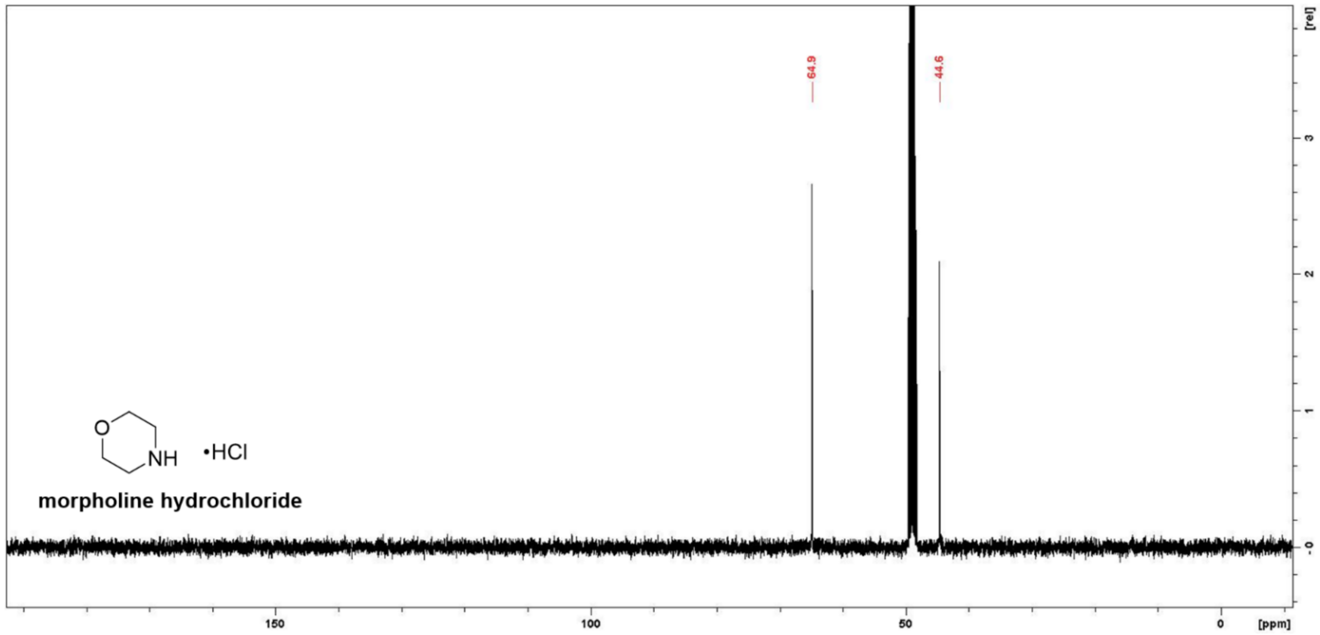

Figure S45. ^{13}C-NMR spectrum of the final product of the oxidation of compound **3**, *i.e.* morpholine hydrochloride.

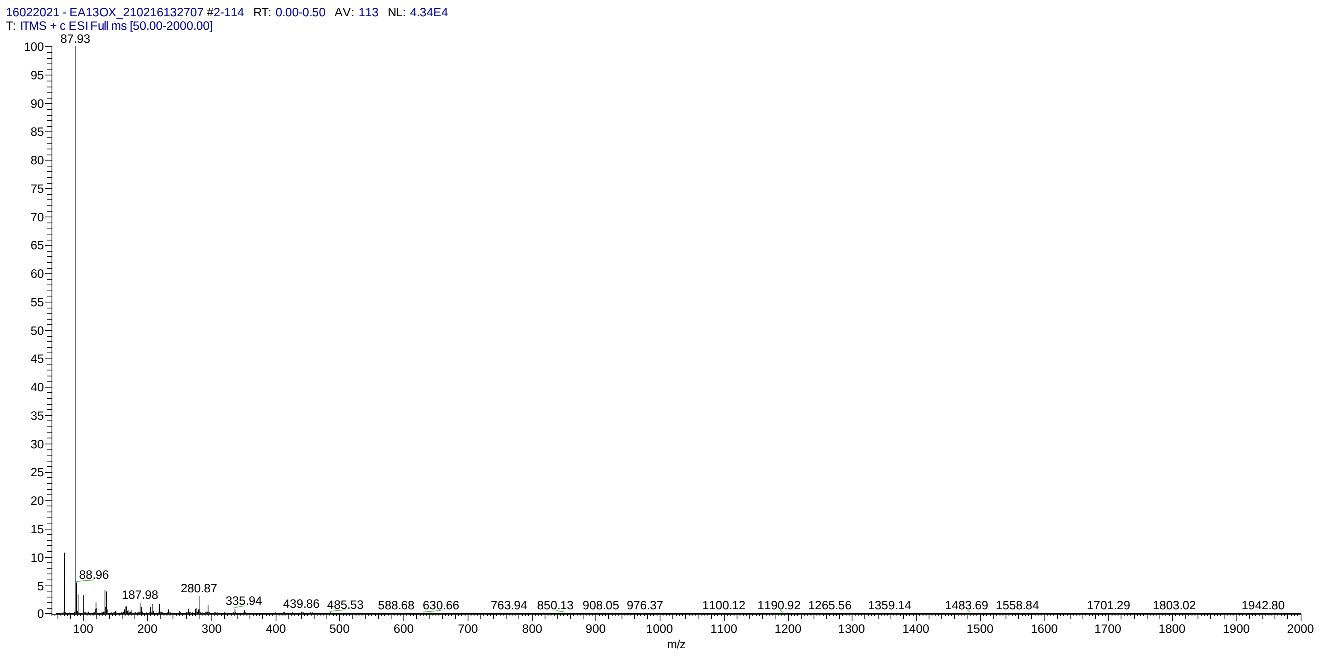

Figure S46. ESI-MS spectrum of the final product of the oxidation of compound **3**, *i.e.* morpholine hydrochloride.

Isoindoline•HCl

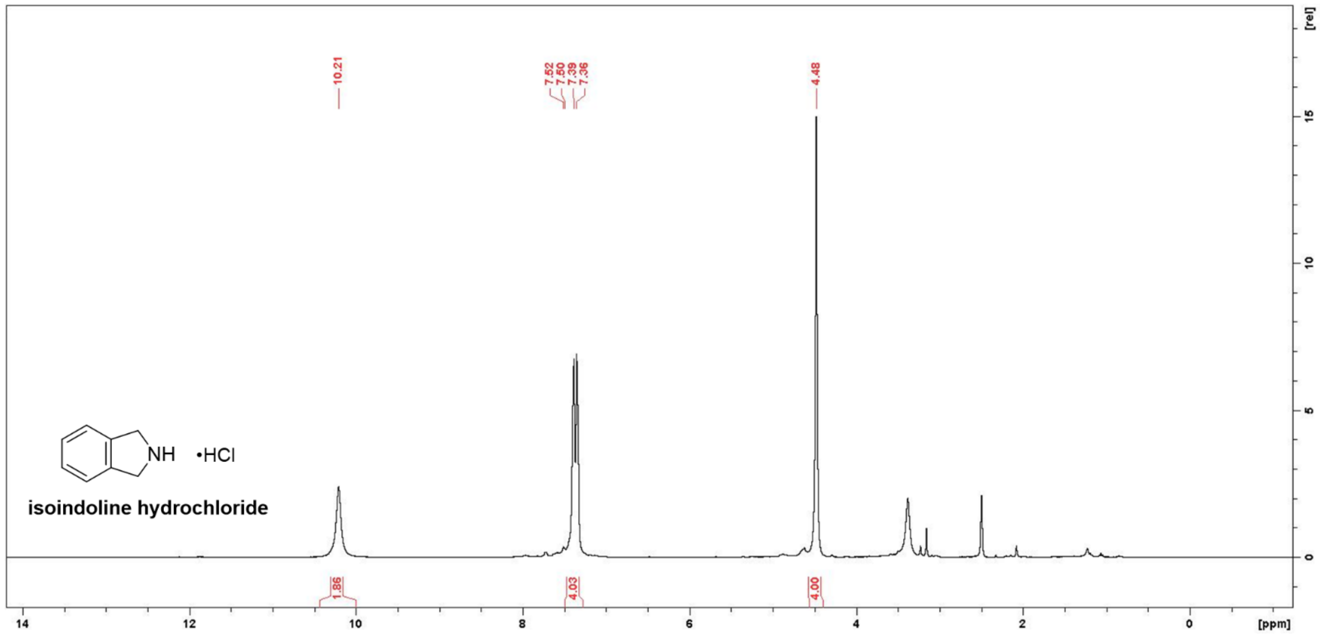

Figure S47. ^{1}H-NMR spectrum of the final product of the oxidation of compound **4**, *i.e.* isoindoline hydrochloride.

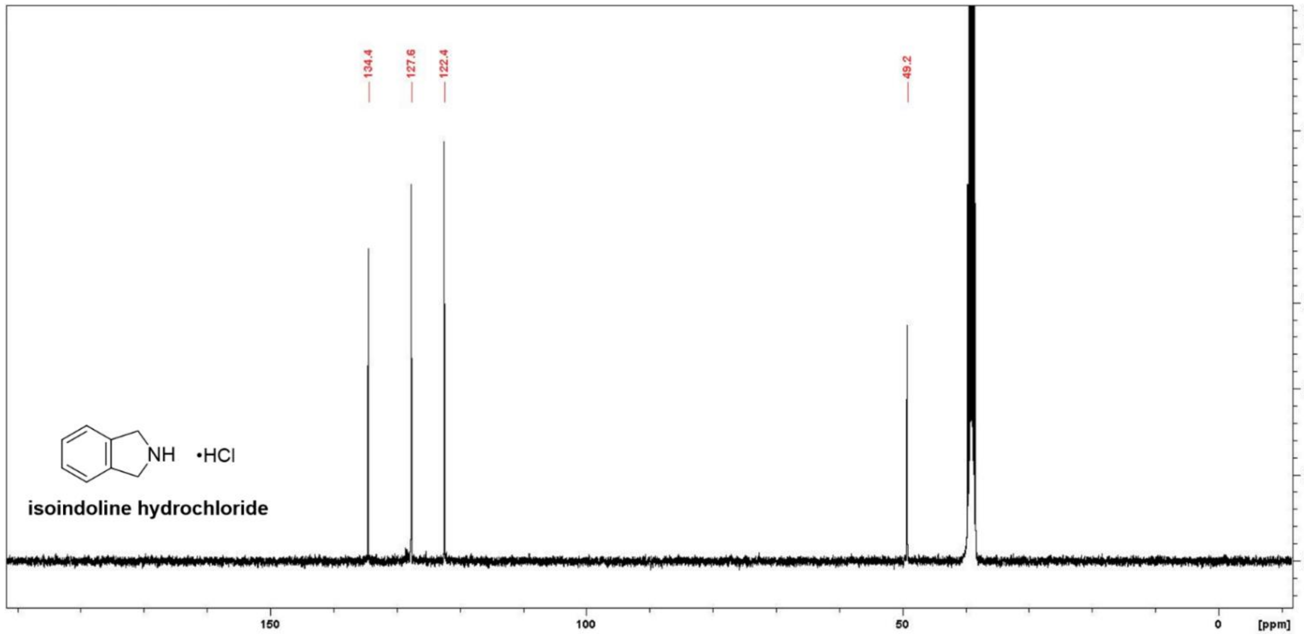

Figure S48. ^{13}C-NMR spectrum of the final product of the oxidation of compound **4**, *i.e.* isoindoline hydrochloride.

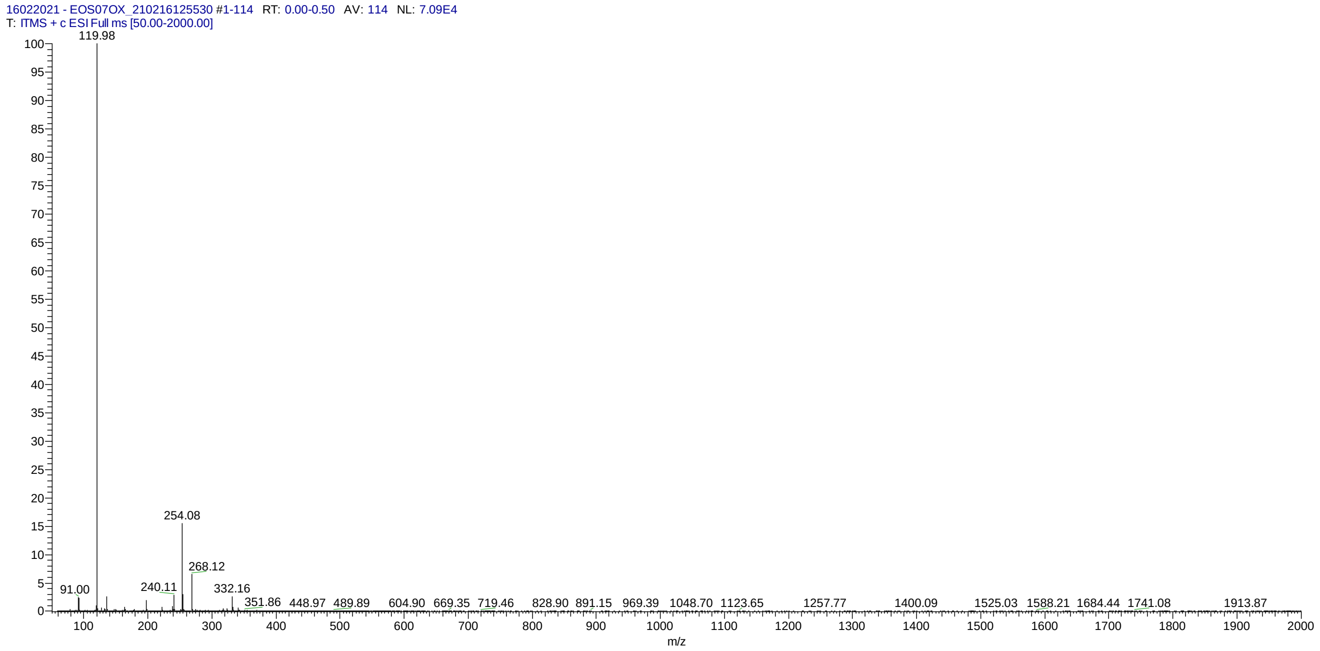

Figure S49. ESI-MS spectrum of the final product of the oxidation of compound **4**, *i.e.* isoindoline hydrochloride.

Benzylamine•HCl

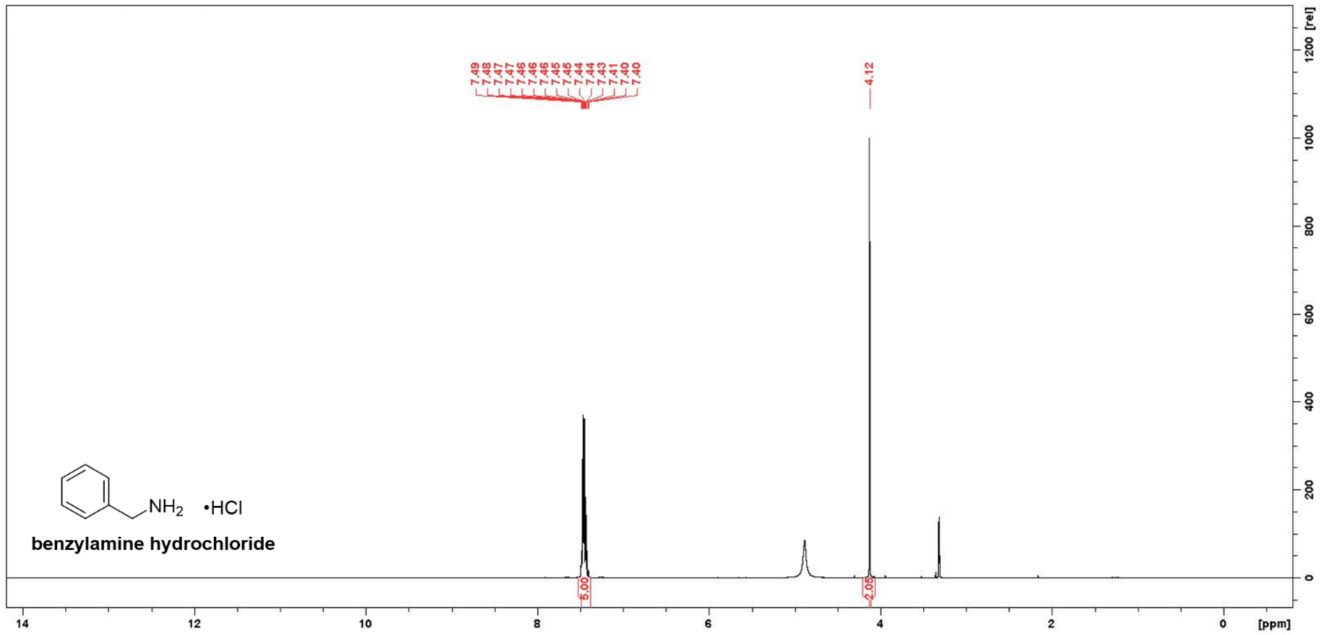

Figure S50. ^{1}H-NMR spectrum of the final product of the oxidation of compound **5**, *i.e.* benzylamine hydrochloride.

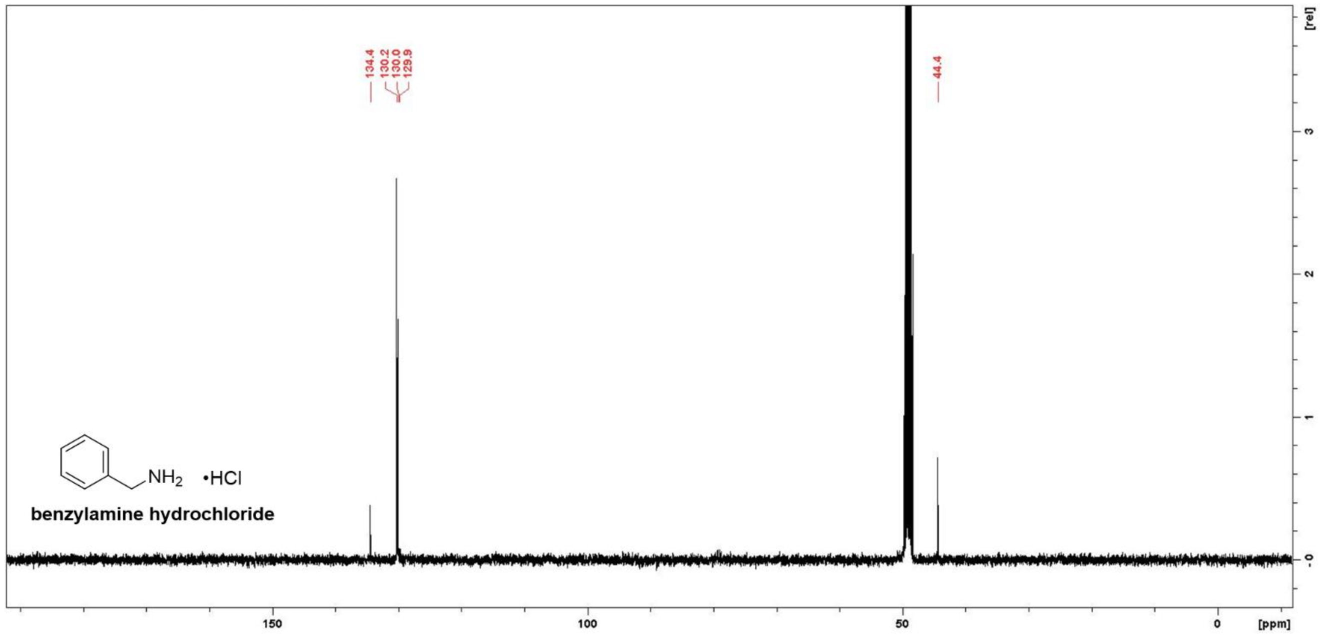

Figure S51. ^{13}C-NMR spectrum of the final product of the oxidation of compound **5**, *i.e.* benzylamine hydrochloride.

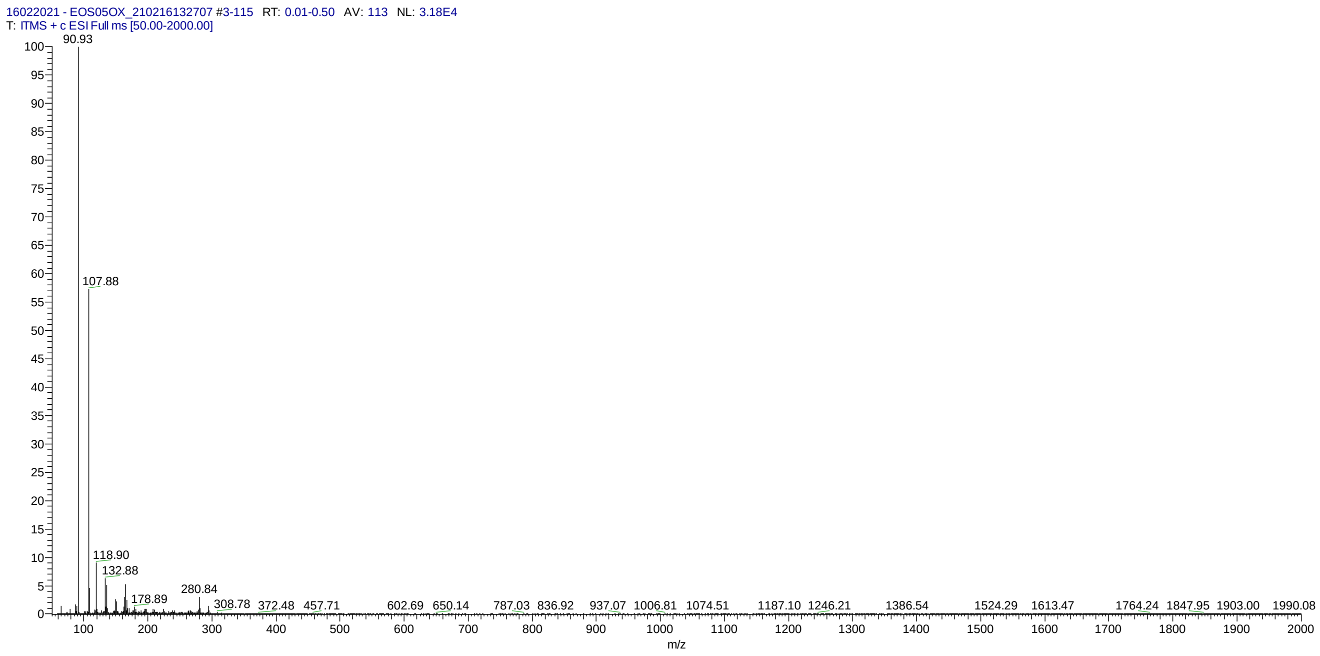

Figure S52. ESI-MS spectrum of the final product of the oxidation of compound **5**, *i.e.* benzylamine hydrochloride.

p-nitrobenzylamine•HCl

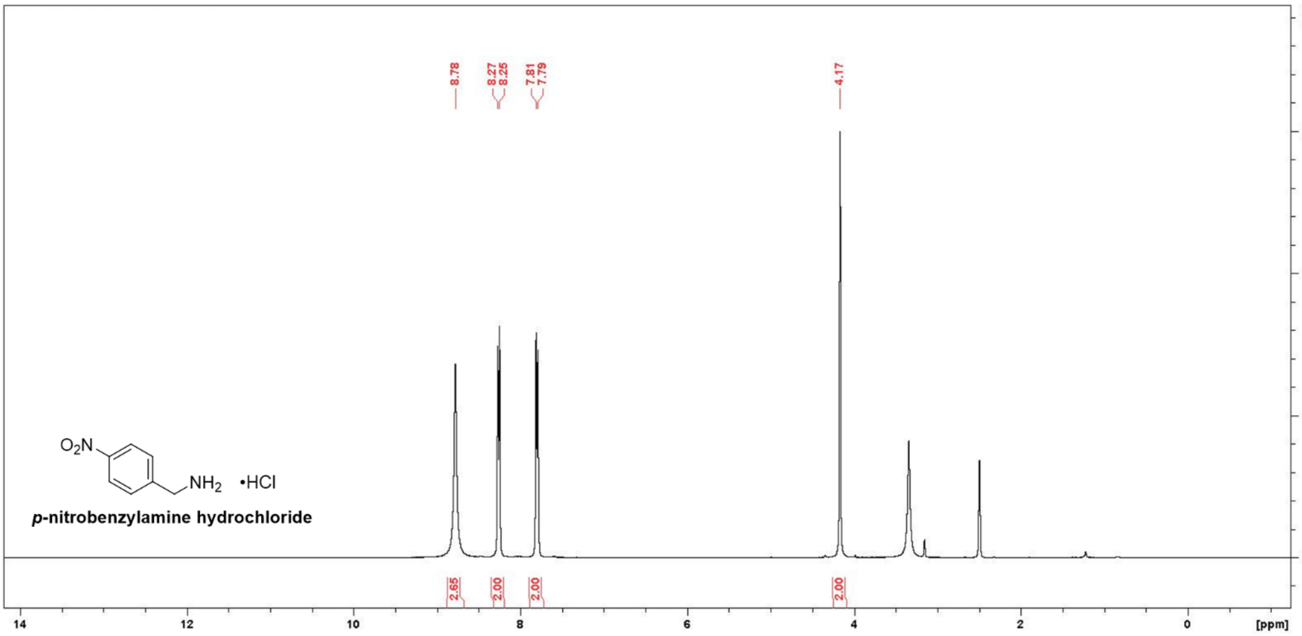

Figure S53. ^{1}H-NMR spectrum of the final product of the oxidation of compound **6**, *i.e. p*-nitrobenzylamine hydrochloride.

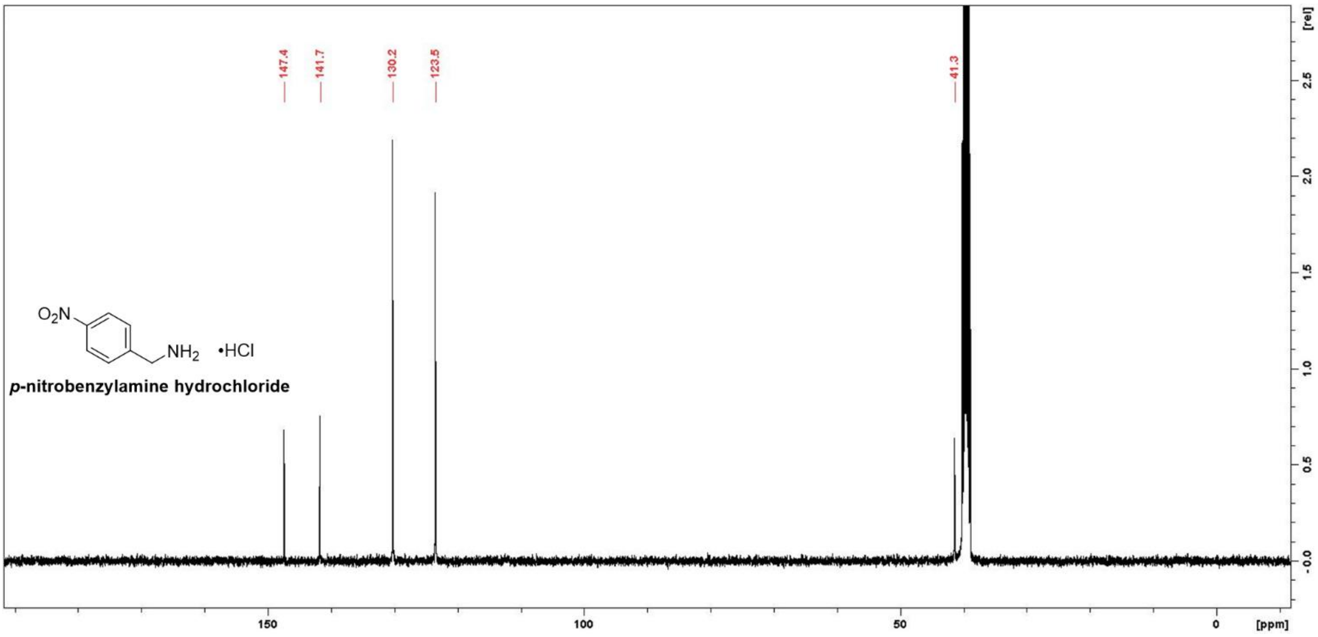

Figure S54. ^{13}C-NMR spectrum of the final product of the oxidation of compound **6**, *i.e. p*-nitrobenzylamine hydrochloride.

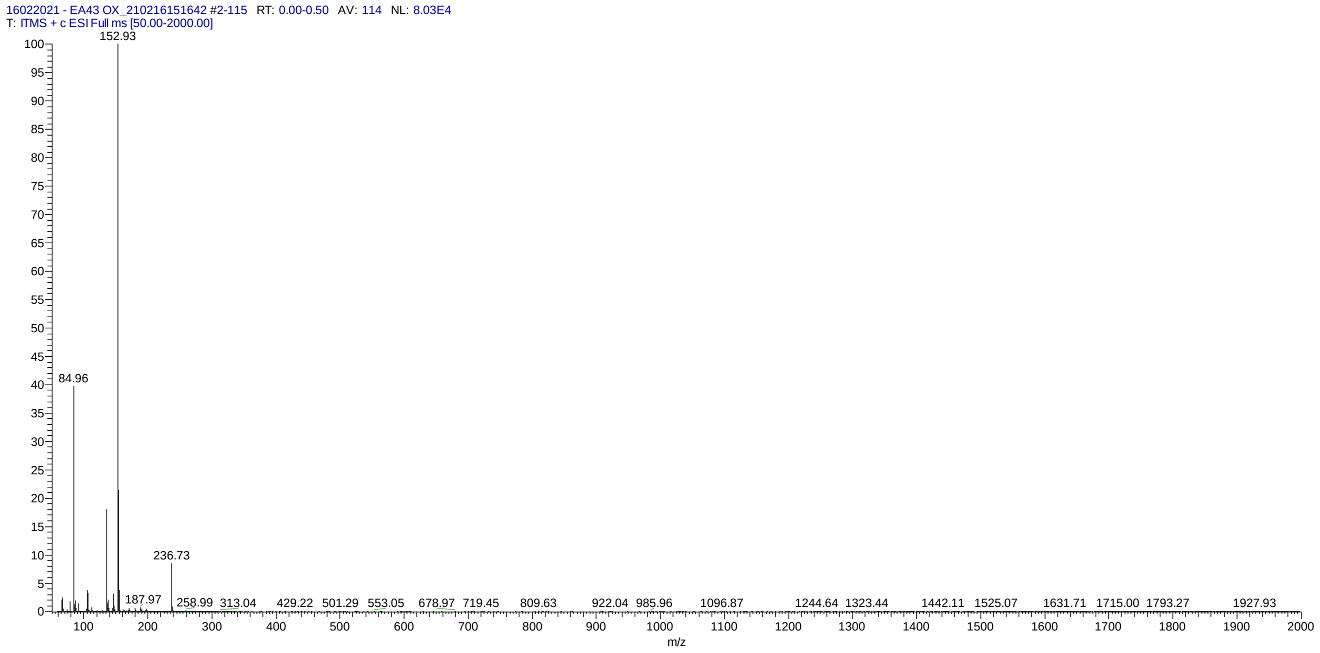

Figure S55. ESI-MS spectrum of the final product of the oxidation of compound **6**, *i.e. p*-nitrobenzylamine hydrochloride.

N-ethylbenzylamine•HCl

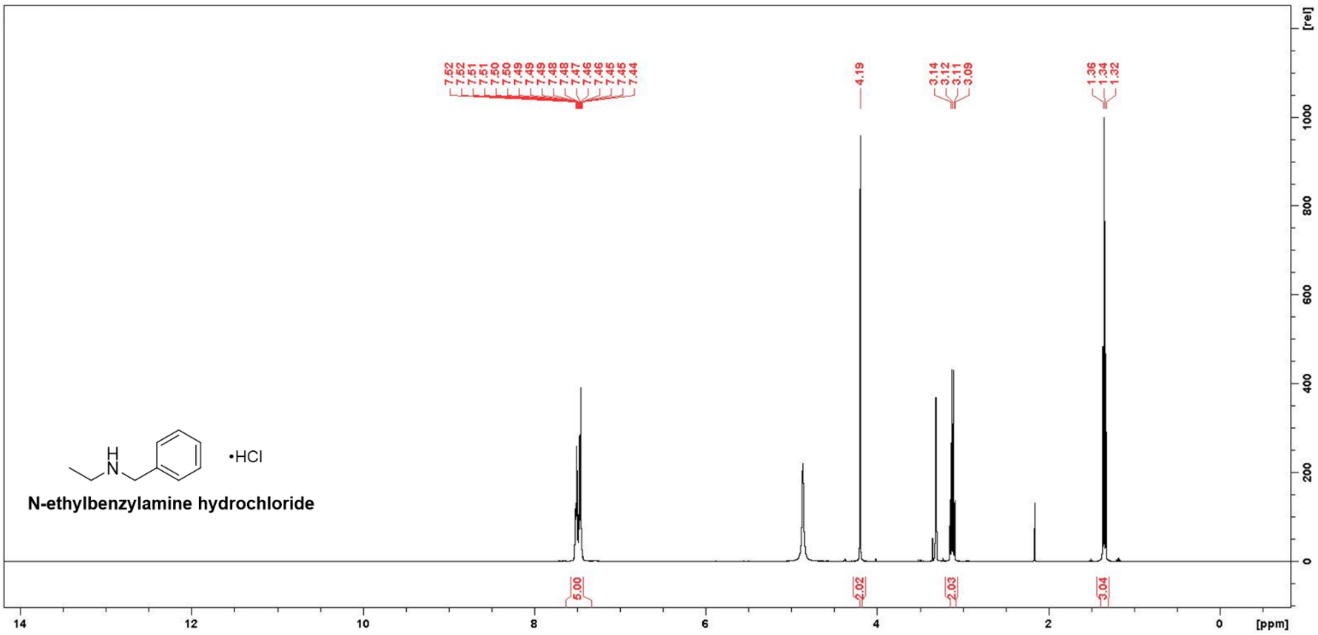

Figure S56. ^{1}H-NMR spectrum of the final product of the oxidation of compound **7**, *i.e.* N-ethylbenzylamine hydrochloride.

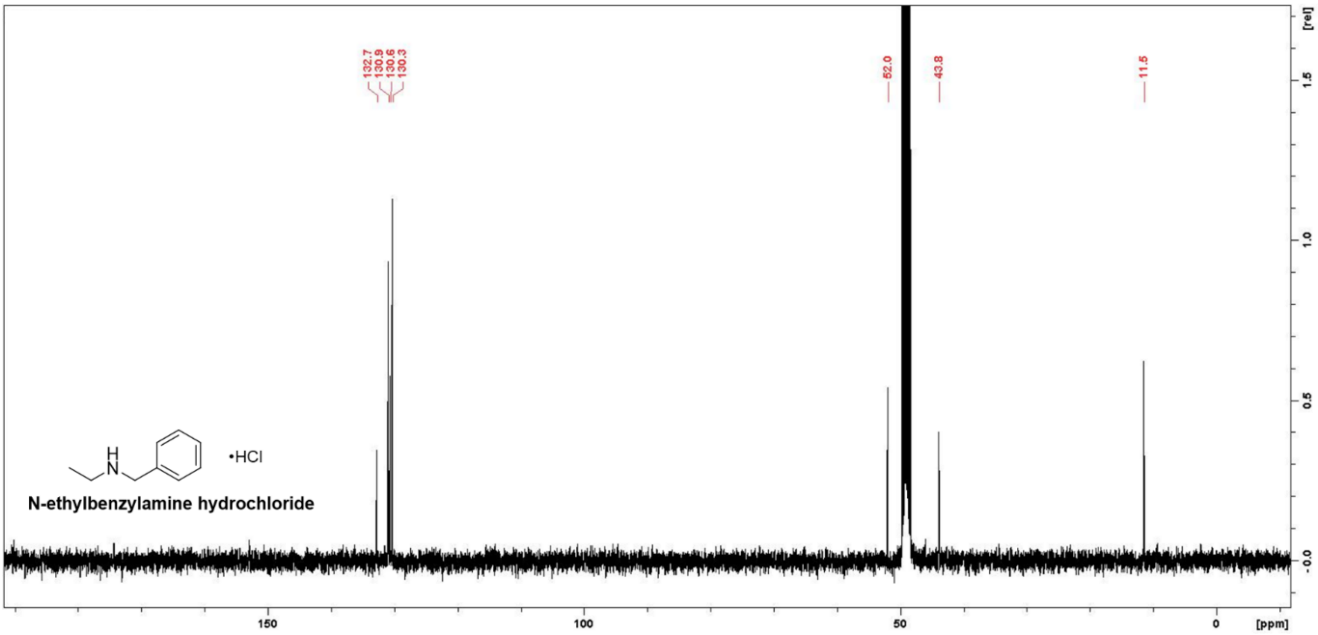

Figure S57. ^{13}C-NMR spectrum of the final product of the oxidation of compound **7**, *i.e.* N-ethylbenzylamine hydrochloride.

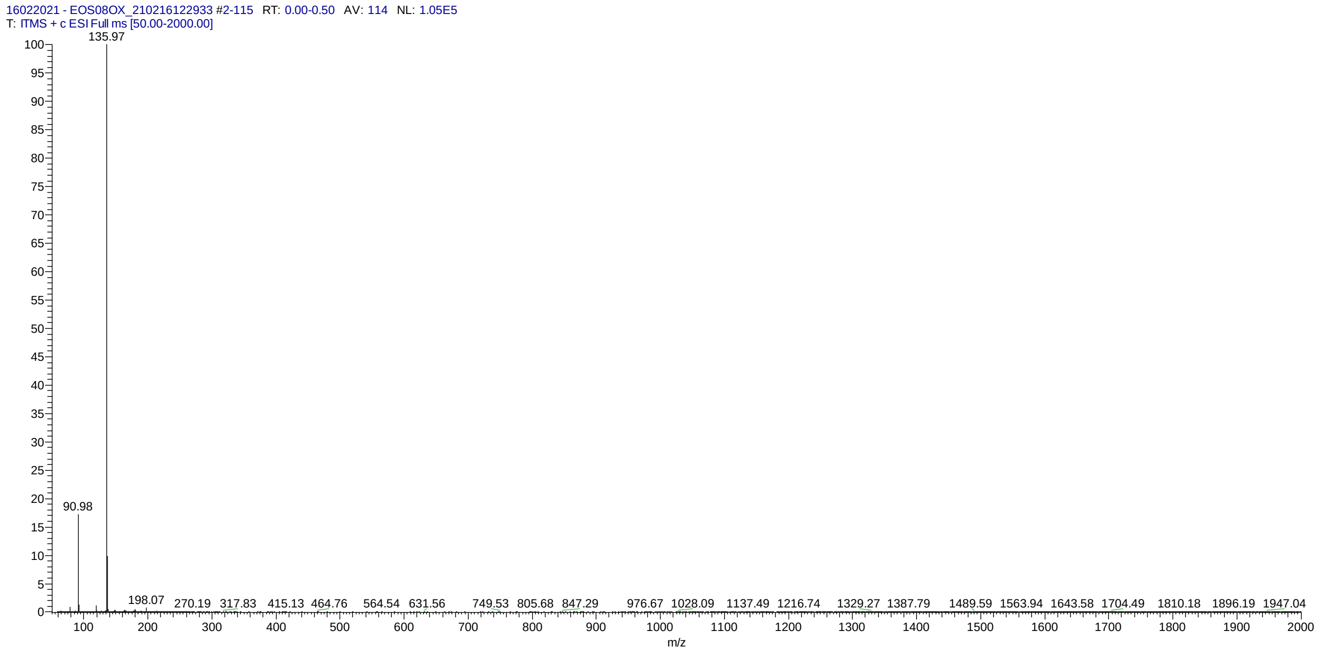

Figure S58. ESI-MS spectrum of the final product of the oxidation of compound **7**, *i.e.* N-ethylbenzylamine hydrochloride.

Table S1. Gibbs free energies relative to free reactants for the selenoxide-triggered amine formation. See Scheme 1 and Figure 1 in the main text for the definition of the structures.

		ΔG (kcal mol^{-1})	
		Gas phase	Water
a)	3 + H_2O_2	0.0	0.0
	TSox	25.0	14.5
	Iox	-34.1	-39.5
	TSelim	-29.3	-31.9
	SeOH + En	-73.5	-76.7
b)	En + H_2O	0.0	0.0
	TS1	50.3	51.8
	I	19.1	25.5
	TS2	-	-
	P	8.8	-6.4

Table S2 Geometries of the optimized structures of the selenoxide-triggered amine formation reaction. Level of theory ZORA-OLYP/TZ2P

H_2O
Energy= -0.51495479 Ha
nimag = 0

O	0.000000000	0.000000000	-0.727963000
H	0.000000000	0.760345000	-0.133368000
H	0.000000000	-0.760345000	-0.133368000

H_2O_2
Energy= -0.65281998Ha
nimag = 0

O	-0.015407000	-0.730641000	-0.690438000
O	0.015407000	0.730641000	-0.690438000
H	0.800760000	0.883499000	-0.140076000
H	-0.800760000	-0.883499000	-0.140076000

3
Energy= -9.0342064 Ha
nimag = 0

C	3.761185000	0.189889000	-0.050204000
C	3.098155000	0.767895000	-1.131872000
C	1.917038000	0.203563000	-1.616144000
C	1.396782000	-0.951374000	-1.022737000
C	2.070711000	-1.537167000	0.055394000
C	3.243908000	-0.962272000	0.543330000
H	3.759653000	-1.423106000	1.382604000
H	1.684025000	-2.446362000	0.506576000
Se	-0.191033000	-1.777482000	-1.753004000
C	-1.555801000	-1.222874000	-0.354902000
C	-1.755219000	0.268484000	-0.309006000
C	-2.273080000	0.992546000	-1.394733000
C	-2.468985000	2.367534000	-1.311732000
C	-2.142982000	3.058192000	-0.142262000
C	-1.616861000	2.356356000	0.939654000
C	-1.424903000	0.977090000	0.854203000
H	-1.017044000	0.441560000	1.707784000
H	-1.352172000	2.880821000	1.854713000
H	-2.292840000	4.133110000	-0.080084000
H	-2.872935000	2.905607000	-2.166156000
H	-2.511747000	0.478921000	-2.321547000
C	-2.804225000	-2.095260000	-0.628536000
N	-3.706661000	-2.185364000	0.514549000
C	-4.778444000	-1.204307000	0.576835000
H	-5.577529000	-1.410324000	-0.162976000
H	-4.388971000	-0.213141000	0.344112000
H	-3.324488000	-1.753522000	-1.538586000
H	-2.446079000	-3.106458000	-0.841595000
H	-1.126828000	-1.562457000	0.588600000
H	1.398262000	0.661451000	-2.452475000
H	3.495778000	1.665193000	-1.600199000
C	-4.219648000	-3.539015000	0.744232000
H	-3.417288000	-4.144439000	1.183901000
H	-4.531561000	-4.036499000	-0.195601000
H	4.679615000	0.632529000	0.327297000
C	-5.439751000	-3.503447000	1.684429000
H	-6.371696000	-3.409200000	1.103830000
H	-5.502891000	-4.437537000	2.250763000
C	-5.351704000	-1.175651000	2.003585000
H	-6.380518000	-0.778143000	1.979326000
H	-4.745334000	-0.527298000	2.644509000
O	-5.338001000	-2.459007000	2.635056000

TSox
Energy= -9.66014791 Ha
nimag = -366.572 cm^{-1}

C	3.783534000	0.351761000	-0.368024000
C	2.911646000	0.786672000	-1.366032000
C	1.757203000	0.062189000	-1.662519000
C	1.484474000	-1.102207000	-0.942811000
C	2.356956000	-1.553827000	0.052195000
C	3.506840000	-0.818528000	0.339155000
H	4.186953000	-1.165706000	1.112954000
H	2.148153000	-2.470083000	0.597560000
Se	-0.056080000	-2.187986000	-1.384606000
C	-1.519200000	-1.416335000	-0.175794000
C	-1.508876000	0.074035000	-0.005816000
C	-1.923027000	0.961247000	-1.011469000
C	-1.936857000	2.334222000	-0.780689000
C	-1.539405000	2.851588000	0.452441000
C	-1.117707000	1.982075000	1.456553000
C	-1.100152000	0.608723000	1.224931000
H	-0.774174000	-0.058653000	2.019360000
H	-0.804080000	2.370486000	2.422335000
H	-1.555699000	3.924429000	0.627751000
H	-2.255920000	3.004031000	-1.575333000
H	-2.207881000	0.591042000	-1.989237000
C	-2.825278000	-2.053011000	-0.698479000
N	-3.909868000	-1.923621000	0.256052000
C	-5.239251000	-1.841170000	-0.327738000
H	-5.583236000	-2.804541000	-0.751673000
H	-5.224017000	-1.126208000	-1.155530000
H	-3.110231000	-1.558890000	-1.627039000
H	-2.630253000	-3.113202000	-0.955229000
H	-1.239288000	-1.885958000	0.769333000
H	1.080613000	0.387178000	-2.445301000
H	3.128359000	1.694589000	-1.923251000
C	-3.856389000	-2.861741000	1.381037000
H	-3.178151000	-2.469825000	2.148708000
H	-3.468831000	-3.852605000	1.072215000
H	4.682359000	0.920968000	-0.144823000
C	-5.251803000	-3.063911000	1.991078000
H	-5.809951000	-3.836578000	1.438433000
H	-5.157666000	-3.408925000	3.025133000
C	-6.231912000	-1.329343000	0.725577000
H	-7.258548000	-1.582974000	0.414086000
H	-6.155274000	-0.240680000	0.818487000
O	-5.979243000	-1.850454000	2.035483000
O	-1.234819000	-0.466695000	-4.931990000
O	-0.795248000	-1.218994000	-3.111485000
H	-0.323023000	-1.631717000	-3.857850000
H	-2.109776000	-0.880408000	-4.974052000

Iox
Energy= -9.22942917 Ha
nimag = 0

C	3.101068000	-0.182863000	-0.027576000
C	2.460392000	0.099444000	-1.234530000
C	1.171378000	-0.377470000	-1.475208000
C	0.534465000	-1.130017000	-0.494377000
C	1.168320000	-1.433866000	0.710159000
C	2.456168000	-0.950175000	0.943891000
H	2.956947000	-1.179393000	1.881469000
H	0.677198000	-2.043789000	1.465757000
Se	-1.243746000	-1.893817000	-0.896896000
C	-2.397926000	-0.563715000	0.246352000
C	-2.221793000	0.843251000	-0.219197000
C	-2.783694000	1.304702000	-1.422324000
C	-2.627755000	2.631717000	-1.816915000
C	-1.911589000	3.527295000	-1.023527000
C	-1.344848000	3.082269000	0.170794000
C	-1.494660000	1.755015000	0.563933000
H	-1.049021000	1.422549000	1.498062000
H	-0.785080000	3.770039000	0.800077000
H	-1.795890000	4.563082000	-1.332857000
H	-3.066844000	2.966036000	-2.753835000
H	-3.329803000	0.621561000	-2.062906000
C	-3.858808000	-1.081340000	0.255233000
N	-4.190159000	-1.885121000	1.425320000
C	-5.579171000	-1.785859000	1.849597000
H	-6.276536000	-2.309028000	1.166790000
H	-5.880447000	-0.734012000	1.854076000
H	-4.098250000	-1.602200000	-0.690955000
H	-4.500634000	-0.197184000	0.279473000
H	-1.973510000	-0.694102000	1.244399000
H	0.658873000	-0.178618000	-2.412369000
H	2.965331000	0.691782000	-1.993980000
O	-1.527826000	-1.496124000	-2.478877000
C	-3.758030000	-3.285269000	1.359648000
H	-2.690273000	-3.342442000	1.600506000
H	-3.887629000	-3.711178000	0.346264000
H	4.106507000	0.188246000	0.154517000
C	-4.557017000	-4.152297000	2.348180000
H	-5.479098000	-4.528365000	1.876802000
H	-3.964946000	-5.023606000	2.643426000
C	-5.714284000	-2.329062000	3.281386000
H	-6.763991000	-2.610243000	3.469803000
H	-5.425403000	-1.561176000	4.007071000
O	-4.855977000	-3.444097000	3.537567000

TSelim
Energy= -9.2134102 Ha
nimag = -607.658 cm^{-1}

C	3.035131000	0.667873000	-3.239411000
C	2.071530000	-0.157099000	-3.821433000
C	1.232306000	-0.935519000	-3.025257000
C	1.361637000	-0.881337000	-1.637088000
C	2.325616000	-0.059951000	-1.044928000
C	3.160157000	0.713332000	-1.850199000
H	3.908216000	1.353989000	-1.389367000
H	2.431959000	-0.016411000	0.037004000
Se	0.263333000	-1.989259000	-0.482988000
C	-2.008227000	-0.362765000	-0.130901000
C	-1.551895000	0.992526000	-0.382050000
C	-1.618238000	1.623945000	-1.643260000
C	-1.211805000	2.942658000	-1.806561000
C	-0.731156000	3.678796000	-0.721912000
C	-0.654208000	3.074101000	0.534238000
C	-1.051453000	1.753304000	0.698721000
H	-0.986312000	1.293498000	1.682258000
H	-0.283905000	3.636005000	1.388330000
H	-0.420868000	4.712011000	-0.854203000
H	-1.272910000	3.403394000	-2.789543000
H	-1.992219000	1.080334000	-2.504272000
C	-2.782456000	-1.198616000	-0.996851000
N	-3.759339000	-2.044357000	-0.350641000
C	-4.919348000	-1.356069000	0.203623000
H	-5.606274000	-0.992100000	-0.585642000
H	-4.591191000	-0.473564000	0.758297000
H	-3.139175000	-0.720237000	-1.924373000
H	-1.916764000	-1.992534000	-1.426421000
H	-1.988269000	-0.670228000	0.911552000
H	0.487807000	-1.588007000	-3.471134000
H	1.973690000	-0.199937000	-4.903840000

O	-0.792214000	-2.784696000	-1.567850000
C	-4.169702000	-3.192201000	-1.167955000
H	-3.377865000	-3.945984000	-1.124167000
H	-4.301839000	-2.914582000	-2.232194000
H	3.687946000	1.270815000	-3.865243000
C	-5.501108000	-3.773091000	-0.659617000
H	-6.355806000	-3.285136000	-1.155714000
H	-5.555954000	-4.840324000	-0.893885000
C	-5.653693000	-2.299172000	1.169885000
H	-6.699461000	-1.965845000	1.284529000
H	-5.175932000	-2.281836000	2.155687000
O	-5.617353000	-3.664312000	0.749556000

SeOH
Energy= -3.0044793 Ha
nimag = 0

Se	-0.229018000	1.753446000	0.064112000
C	-1.666618000	0.470224000	0.042457000
C	-2.974497000	0.948980000	-0.100560000
C	-4.038711000	0.051509000	-0.198858000
C	-3.808511000	-1.320855000	-0.116205000
C	-2.505574000	-1.796860000	0.047901000
C	-1.434347000	-0.908971000	0.108841000
H	-3.162713000	2.018903000	-0.122957000
H	-5.051255000	0.430170000	-0.316024000
H	-4.639913000	-2.018545000	-0.175312000
H	-2.320356000	-2.866534000	0.111672000
H	-0.422480000	-1.290547000	0.208833000
O	0.664358000	1.259933000	1.594612000
H	1.391704000	0.698247000	1.292068000

En
Energy= -6.17730343 Ha
nimag = 0

C	-1.278426000	-0.047094000	0.699708000
C	-1.118337000	1.278360000	-0.045748000
C	-0.062136000	1.467118000	-0.943876000
C	0.080396000	2.682196000	-1.610835000
C	-0.833985000	3.713048000	-1.393040000
C	-1.891620000	3.528130000	-0.503291000
C	-2.038761000	2.313462000	0.163709000
H	-2.860811000	2.188353000	0.864882000
H	-2.601123000	4.330995000	-0.323373000
H	-0.718620000	4.660907000	-1.911299000
H	0.912950000	2.825904000	-2.293892000
H	0.665530000	0.676277000	-1.110193000
C	-2.292252000	-0.854000000	-0.020954000
N	-3.418962000	-1.323774000	0.408576000
C	-3.911331000	-1.286986000	1.793732000
H	-4.902211000	-0.825369000	1.759991000
H	-3.271294000	-0.671782000	2.420979000
H	-2.091311000	-1.040658000	-1.075424000
H	-1.515427000	0.112233000	1.751696000
C	-4.341102000	-2.059447000	-0.499573000
H	-3.744058000	-2.468594000	-1.315648000
H	-5.060412000	-1.340097000	-0.908584000
C	-5.054456000	-3.178552000	0.290282000
H	-6.001055000	-2.822568000	0.721032000
H	-5.285637000	-4.004015000	-0.386982000
C	-3.986347000	-2.740475000	2.331560000
H	-4.795570000	-2.790056000	3.075176000
H	-3.046888000	-3.021010000	2.815549000
O	-4.185701000	-3.681583000	1.287291000
H	-0.333754000	-0.604725000	0.655401000

TS1
Energy= -6.630218 Ha
nimag = -1248.841

C	-2.410395000	0.597507000	0.869768000
C	-1.608853000	1.673063000	0.160966000
C	-0.222448000	1.756280000	0.334986000
C	0.514478000	2.740417000	-0.323398000
C	-0.125860000	3.651437000	-1.161947000
C	-1.507995000	3.580581000	-1.335004000
C	-2.245343000	2.598342000	-0.676267000
H	-3.326089000	2.572264000	-0.803031000
H	-2.015853000	4.298325000	-1.973368000
H	0.447863000	4.421853000	-1.669571000
H	1.588850000	2.799561000	-0.172123000
H	0.288012000	1.062056000	0.998400000
C	-2.715613000	-0.581383000	-0.024680000
N	-3.457000000	-1.754237000	0.435142000
C	-3.667643000	-1.987365000	1.883565000
H	-4.506106000	-1.378064000	2.238456000
H	-2.779320000	-1.693219000	2.443014000
H	-3.080343000	-0.273961000	-1.005348000
H	-3.383971000	0.997038000	1.183341000
C	-4.685535000	-2.069869000	-0.352592000
H	-4.374959000	-2.315020000	-1.372327000
H	-5.347798000	-1.195242000	-0.389070000
C	-5.414051000	-3.265118000	0.298396000
H	-6.180495000	-2.918524000	1.006661000
H	-5.916106000	-3.853664000	-0.473133000
C	-3.929237000	-3.497992000	2.092336000
H	-4.596512000	-3.626108000	2.957506000
H	-2.995207000	-4.029430000	2.295596000
O	-4.477331000	-4.114093000	0.932455000
O	-1.408095000	-1.412507000	-0.301985000
H	-1.089316000	-1.350323000	-1.221734000
H	-2.161721000	-2.228564000	-0.073446000
H	-1.896585000	0.265953000	1.774772000

I
Energy= -7.21586 Ha
nimag = 0

C	-1.689773560	-0.176055253	0.274698973
C	-1.180583954	1.176479459	-0.184466198
C	-0.712008357	1.360703468	-1.490331173
C	-0.250388622	2.608541965	-1.907067418
C	-0.252506077	3.688421011	-1.024974942
C	-0.713723660	3.514091730	0.279425234
C	-1.173343778	2.265825510	0.696121812
H	-1.509014845	2.141263962	1.724103570
H	-0.704224229	4.346314430	0.978170216
H	0.114723481	4.658570290	-1.348466635
H	0.119661421	2.733820677	-2.921199083
H	-0.698783278	0.525618374	-2.185680151
C	-3.206757784	-0.300694525	0.076308928
N	-3.755938768	-1.513489246	0.958182156
C	-5.244554043	-1.429640889	1.191154242
H	-5.718072891	-1.207659483	0.233513102
H	-5.408598423	-0.592162728	1.872608066
H	-3.718969584	0.559404075	0.522573948
H	-1.445473909	-0.307814062	1.335989833
C	-3.396333218	-2.884305477	0.458377719
H	-2.337805748	-2.904736757	0.202419400
H	-3.970327854	-3.045510769	-0.453078449
C	-3.719660521	-3.908942461	1.570142150

H	-4.061223507	-4.845477104	1.106461644
H	-2.834641933	-4.126077175	2.174508810
C	-5.750415802	-2.761210442	1.788969636
H	-6.164986134	-3.413929701	1.008143544
H	-6.539491653	-2.563762188	2.517989397
O	-4.691576481	-3.403698444	2.481308460
O	-3.556439400	-0.514937639	-1.227605581
H	-4.285437584	0.084510349	-1.500755310
H	-3.316471815	-1.410571933	1.875450134
H	-1.189132690	-0.970779896	-0.284725159
O	-5.622590065	1.209880590	-1.987871528
H	-6.194293976	0.949854434	-2.722903490
H	-5.375879288	2.123489857	-2.187005281

Phenylacetaldehyde
Energy= -3.81886467 Ha
nimag = 0

C	-0.919900000	2.350100000	0.979500000
C	-0.152300000	3.023500000	-0.146400000
C	0.468900000	2.245000000	-1.131500000
C	1.167100000	2.849200000	-2.175500000
C	1.245400000	4.239400000	-2.253800000
C	0.625200000	5.022100000	-1.280800000
C	-0.070600000	4.417900000	-0.234400000
H	-0.536900000	5.039800000	0.526300000
H	0.689600000	6.105800000	-1.329400000
H	1.792800000	4.710400000	-3.065800000
H	1.654500000	2.232400000	-2.926100000
H	0.421200000	1.159800000	-1.076300000
C	-2.313300000	2.001200000	0.535700000
H	-2.905300000	2.846700000	0.124300000
H	-1.021000000	3.047100000	1.823200000
O	-2.799300000	0.879800000	0.584200000
H	-0.420000000	1.447300000	1.340000000

Morpholine
Energy= -2.85890334 Ha
nimag = 0

N	-3.771121000	-2.184624000	0.602577000
C	-3.434790000	-3.044042000	1.796643000
H	-3.483803000	-2.391797000	2.670587000
H	-2.409051000	-3.400768000	1.681545000
C	-5.140021000	-1.543740000	0.727921000
H	-5.416932000	-1.202733000	-0.271917000
H	-5.037411000	-0.678066000	1.386294000
C	-6.110988000	-2.612231000	1.284658000
H	-6.277452000	-2.471621000	2.360846000
H	-7.074552000	-2.541474000	0.776293000
C	-4.465368000	-4.200918000	1.837224000
H	-4.771474000	-4.381194000	2.876485000
H	-4.036485000	-5.121469000	1.434384000
O	-5.586439000	-3.904311000	1.010776000
H	-3.774704000	-2.779152000	-0.230980000
H	-3.057621000	-1.466617000	0.460865000
